# Westernized diet related arachidonic acid metabolism expands DNA error-prone growth factor-dependent colonic quiescent stem cells in adolescent mice

**DOI:** 10.1038/s41419-026-09240-9

**Published:** 2026-09-14

**Authors:** Lea Christiansen, Mohab Ragab, Laura Nickel, Annika Sünderhauf, Lina Jegodzinski, Maren Hicken, Heidi Schlichting, Rudrik Weikamp, Franziska Schmelter, Daniel Margis, Stefan Mertel, Ines Richerzhagen, Henrike Wenk, Luise Pötter, Sebastian Tornow, Nele Buchmann, Daniel Vondran, Clarissa Gottschild, Annika Raschdorf, Larissa Nogueira de Almeida, OUTLIVE-CRC consortium, Nikolas von Bubnoff, Alexander Katalinic, Martina Oberländer, Ruth Deck, Holger Sültmann, Tobias Hutzenlaub, Peter Jülg, Hauke Busch, Timo Gemoll, Christian Sina, Stefanie Derer, Axel Künstner, Hauke Busch, Timo Gemoll, Christian D. Sadik, Darko Castven, Jens U. Marquardt, Senad Divanovic, Walter Raasch, Christian Sina, Stefanie Derer

**Affiliations:** 1 University of Luebeck, Institute of Nutritional Medicine, University Hospital Schleswig-Holstein, Campus Luebeck, Luebeck, Germany; 2 Department of Internal Medicine II, Klinikum rechts der Isar, TUM School of Medicine and Health, Technical University of Munich, Munich, Germany; 3 University of Luebeck, Institute of Experimental and Clinical Pharmacology and Toxicology, Luebeck, Germany; 4 2nd Department of Medicine, Sana Clinics Luebeck, Luebeck, Germany; 5 University of Luebeck, Department of Medicine I, University Hospital Schleswig-Holstein, Luebeck, Germany; 6 University of Luebeck, Medical Systems Biology Group, Luebeck Institute of Experimental Dermatology, Luebeck, Germany; 7 University Cancer Center Schleswig-Holstein, University Hospital Schleswig-Holstein, Campus Luebeck, Luebeck, Germany; 8 University of Luebeck, Section for Translational Surgical Oncology & Biobanking, Department of Surgery, University Medical Center Schleswig-Holstein, Campus Luebeck, Luebeck, Germany; 9 University of Luebeck, Department of Dermatology, Allergy, and Venereology, Luebeck, Germany; 10 University of Luebeck, Center for Research on Inflammation of the Skin (CRIS), Luebeck, Germany; 11 Department of Pediatrics, University of Cincinnati College of Medicine, Cincinnati, OH USA; 12 Division of Immunobiology, Cincinnati Children’s Hospital Medical Center, Cincinnati, OH USA; 13 DZHK (German Centre for Cardiovascular Research), Partner Site Hamburg/Kiel/Luebeck, Luebeck, Germany; 14 University of Luebeck, CBBM (Centre of Brain, Behaviour and Metabolism), Luebeck, Germany; 15 University of Luebeck, Department of Hematology and Oncology, University Hospital of Schleswig-Holstein, Campus Luebeck, Luebeck, Germany; 16 University of Luebeck, Institute for Social Medicine and Epidemiology, Luebeck, Germany; 17 University of Luebeck, Interdisciplinary Center for Biobanking-Luebeck (ICB-L), Luebeck, Germany; 18 Division of Cancer Genome Research, German Cancer Research Center (DKFZ), German Cancer Consortium (DKTK), National Center for Tumor Diseases (NCT), Heidelberg, Germany; 19 German Center for Lung Research (DZL), TLRC Heidelberg, Heidelberg, Germany; 20 Hahn-Schickard Society, Freiburg, Germany; 21 Laboratory for MEMS Applications, IMTEK-Department of Microsystems Engineering, University of Freiburg, Freiburg, Germany

**Keywords:** Intestinal stem cells, Colorectal cancer, DNA mismatch repair

## Abstract

Given the association between early-onset colorectal cancer (eoCRC) and insulin resistance (IR), we investigated the effects of westernized diet (WD)-induced IR on intestinal stem cells (ISCs) by in-depth phenotyping three adolescent mouse models of escalating WD-driven IR. WD-related hyperlipidaemia and hyperglycaemia enhanced ω6-polyunsaturated fatty acid (PUFA) metabolism, while inhibiting glucose metabolism, resulting in colonic epithelial cell (EC) energy deficiency, hypoproliferation, and quiescent ISCs (qISC) expansion. WD-induced systemic IR provoked colonic EC IR, reduced ω6-PUFA metabolism, which was compensated by aberrated triglyceride metabolism and growth factor receptor signalling, causing hyperproliferation of expanded qISCs. Combined with single-cell transcriptomic data and colonic 3D-organoid analysis we revealed that the ω6-PUFA arachidonic acid inhibited glucose uptake, favouring colonic EC IR driven expansion of the lipid-dependent inherently DNA-mismatch prone qISC compartment in expense of the insulin-dependent LGR5^+^ ISCs. Overall, our findings may open new alleys for targeted nutritional interventions in eoCRC prevention.

## Introduction

Colorectal cancer (CRC) is the third most common cancer and estimated to reach 3.2 million new global cases in 2040 [1]. Recent epidemiological evidence confirms that the incidence of early-onset colorectal cancer (eoCRC) is increasing globally, particularly in individuals under 50 years of age, with the rise either exclusive to early-onset disease or faster than the increase in older adults [2]. This trend is consistently reported across multiple high-income countries [2–5]. Emerging data further indicate that early-life exposures, particularly dietary patterns during adolescence and young adulthood, may contribute to eoCRC risk later in life [3]. Consumption of ultra-processed, high-fat foods are associated with a tenfold increased risk of developing CRC, and high-fat diets increase the risk of eoCRC by almost twofold [6, 7].

Early-life exposure to lifestyle-associated metabolic dysregulation may represent a major contributor to eoCRC, as eoCRC incidence closely parallels global trends in the prevalence of metabolic disorders, including obesity and type 2 diabetes [8]. In particular, overweight and obesity have been identified as disease-onset-specific risk factors, especially in men [9].

Fatty acids (FAs) are primary constituents of dietary fat and play a critical role as signalling molecules, acting as secondary messengers in physiological regulation and modulating various cellular signalling pathways [10]. Fat-enriched diets include a mixture of FAs from diverse food sources, processed foods, and cultural differences. However, some trends in the types of commonly found FA composition include: monounsaturated FAs (MUFAs; e.g., oleic acid, C18:1), saturated FAs (e.g., palmitic acid, C16:0, and stearic acid, C18:0) and polyunsaturated FAs (PUFAs), commonly ω6 FAs such as linoleic acid (LA, C18:2 ω6), arachidonic acid (AA, C20:4 ω6), as well as ω3 fatty acids (e.g. alpha-linolenic acid, C18:3 ω3). Until a century ago, the dietary ω6 to ω3 ratio was 4:1 or lower. In contrast, the modern Western-style Diet (WD) now features a ratio of approximately 20:1, heavily skewed towards ω6 fatty acids [11, 12]. While ω3 PUFAs give rise to anti-inflammatory mediators such as prostaglandin E3 (PGE3), leukotriene B5 (LTB5), and docosahexaenoic acid (DHA), ω6-PUFAs are converted into the pro-inflammatory prostaglandin E2 (PGE2) and leukotriene A4 (LTA4). This imbalance promotes inflammation, as ω6 and ω3 PUFAs compete for the same enzymes, including Δ6/Δ5 desaturases, elongases, cyclooxygenases (COX), and lipoxygenases (ALOX) [13]. Consequently, an ω6-skewed ratio fosters persistent low-grade inflammation [14, 15]. Eicosanoid production occurs in distinct phases of tissue repair in a temporally regulated manner. Therefore, if WD-induced damage to colonic tissue combined with excessive intake of ω6-PUFAs leads to a prolonged inflammatory phase and impaired resolution of inflammation, this may result in chronic inflammation and increased tumorigenesis risk [14, 16, 17]. Notably, elevated levels of AA have been identified in colon cancer tissues, suggesting a pro-proliferative and pro-inflammatory role for AA within the intestinal environment [18].

Intestinal stem cells (ISC) reside at the bottom of the colonic crypt and actively divide to give rise to progenitor cells, which can be categorized into three groups: secretory, absorptive, and stem cell-like progenitors. The secretory and absorptive progenitors proliferate and migrate upward along the crypt, progressively differentiating until they reach the upper crypt region, where they remain before being shed into the lumen [19]. This renewal process takes place every three to five days, is regulated by a variety of differentiation signals and is accompanied by a metabolic switch from glycolysis in ISC to oxidative phosphorylation (OXPHOS) in differentiated cells [20]. The stem cell-like progenitor cell, also called quiescent intestinal stem cell (qISC), remains usually in the +4-position of the colonic crypt and is activated when the active ISC is threatened with damage [21, 22]. Recent mechanistic studies indicate that FA metabolism is a central regulator of intestinal stem-cell homeostasis. Dietary lipid exposure and different metabolic states alter ISC self-renewal, differentiation, and tumour susceptibility through pathways involving fatty acid oxidation, PPAR signalling, inflammatory lipid mediators, and nutrient sensing. Emerging evidence further suggests that AA metabolism contributes to epithelial stress signalling, mitochondrial dynamics, and stem-cell plasticity [23–26]. Further, it has been shown that long-term exposure to a WD markedly reduced the population of active stem cells in the murine colon, while qISC emerged as the principal precursors giving rise to all other colonic cell types [22]. Hence, metabolic environmental changes directly affect the colonic crypt homeostasis, increasing the risk for CRC tumorigenesis [27].

Here, we address the impact of diet-induced colonic epithelial cell (EC) insulin resistance during early adolescence on the colonic crypt homeostasis in mice and its implications on spontaneous colon tumour development.

## Results

### Constant HFD consumption during adolescence is accompanied by systemic and colonic EC insulin resistance-like stage in mice

**Fig. 1:**
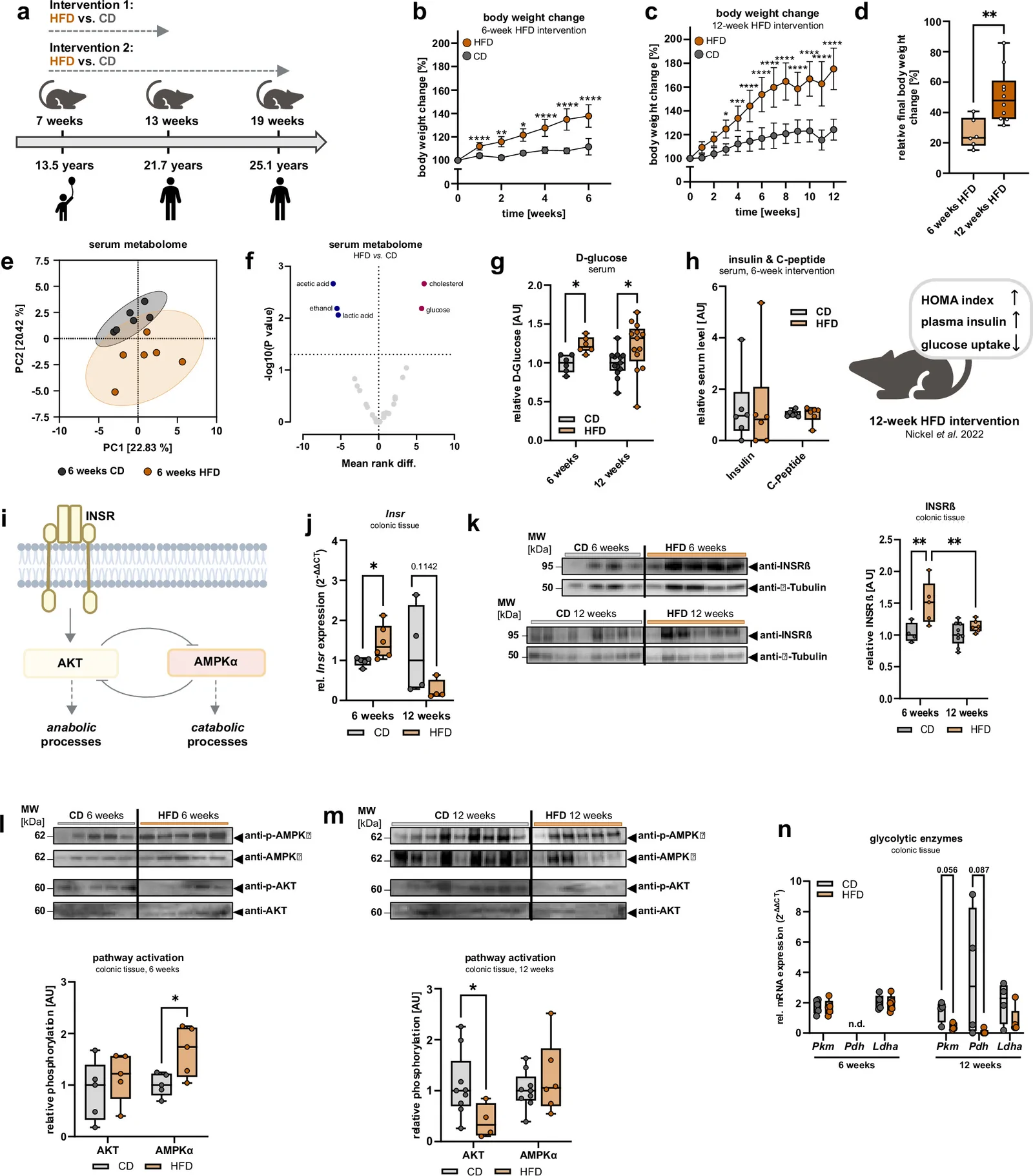
Insulin resistance is associated with the onset of colonic EC insulin resistance in mice. **a** Schematic overview of two independent HFD-mouse models (6 weeks *n *= 6 mice per group; 12 weeks *n *= 13 mice per group) with equivalent human age calculation based on Wang et al. [28]. **b**, **c** Time-dependent change of body weight [%] of mice fed for 6 weeks (*n *= 6 mice per group) or 12 weeks (*n *= 13 mice per group). **d** Comparison of final body weight increase [%] under HDF-feeding relative to CD between mice on HFD for 6 weeks (*n *= 6) or 12 weeks (*n *= 10). **e** Principal component analysis (PCA) of metabolites measured in serum samples of HFD-fed (brown, *n *= 6) and CD-fed (grey, *n *= 6) mice after 6 weeks intervention, using 1H nuclear magnetic resonance (NMR) spectroscopy in-vitro Diagnostic Research (IVDr) metabolomics. **f** Volcano plot of differentially abundant metabolites in serum (*n *= 6 mice per group) of mice after 6 weeks (red: significantly increased, blue: significantly decreased) identified by NMR IVDr metabolomics, displaying unadjusted *P*-values. **g** Relative D-glucose concentration in serum of mice after 6 weeks (CD *n *= 6; HFD *n *= 6) and 12 weeks (CD *n *= 13; HFD *n *= 13), showing unadjusted *P*-values. **h** Left: Relative insulin and C-peptide concentration in serum of mice after 6-week intervention (HFD *n *= 6, CD *n *= 6). Right: Schematic summary of insulin status in mice after 12-week intervention [29]. **i** Scheme depicting the main insulin receptor downstream signalling pathway. **j** Relative expression of *Insr* in murine colonic tissue of HFD-fed mice compared to CD-fed littermates (6 weeks: HFD *n *= 6, CD *n *= 5; 12 weeks: HFD *n *= 4, CD *n *= 4), displaying adjusted *P*-values. **k** Left: INSRβ detection in murine colonic tissue after 6 weeks (HFD *n *= 5, CD *n *= 4) and after 12 weeks (HFD *n *= 6, CD *n *= 9) via Western blotting. Right: INSRβ quantification in colonic tissue of mice after 6- or 12-week HFD-feeding (6 weeks *n *= 5; 12 weeks *n *= 6) relative to CD-fed controls (6 weeks *n *= 4; 12 weeks *n *= 9) *via* Western blotting. **l** Top: Phosphorylation status of AKT and AMPKα in murine colonic tissue after 6 weeks (*n *= 5 per group) via Western blot analysis. Bottom: Relative quantification of phosphorylation status (of AKT, and AMPKα in murine colonic tissue after 6 weeks (*n *= 5 mice per group). **m** Top: Western blot analysis of phosphorylation status of AKT, and AMPKα in 12-week dietary intervention (HFD *n *= 6, CD *n *= 9). Bottom: Quantification of relative phosphorylation status after 12-week dietary intervention (HFD *n *= 4 (AKT) and *n *= 6 (AMPKα) mice, CD each AKT and AMPKα *n *= 9 mice). **n** Relative expression of glycolytic enzymes in murine colonic tissue after 6-week (HFD *n *= 5-6; CD *n *= 5) and 12-week (HFD *n *= 5, CD *n *= 4) intervention measured by RT-qPCR, displaying unadjusted *P*-values. **b**, **c**, **h** Two-way ANOVA with Šidák multiple comparisons test or **k**–**n** Fisher’s least significant difference (LSD) test. **d**, **f**, **g**, **j** Two-tailed (Multiple) Mann-Whitney *U* test. **b**, **c** Data shown as mean ±S.D. and **d**, **g**, **h**, **j**–**n** box plots depicting the median and IQR with whiskers indicating the range from at least three different mice. Relative values determined by normalisation to the median of the respective control group (set to 1). *P ≤ 0.05, **P ≤ 0.01, ***P ≤ 0.001, ****P ≤ 0.0001. CD control diet, Insr insulin receptor, HFD high-fat diet, IQR interquartile range, IVDr in vitro diagnostic research, Ldha lactate dehydrogenase A, Pdh pyruvate dehydrogenase, Pkm pyruvate kinase isozyme M, S.D. standard deviation.

To determine obesity-related colonic phenotypes after high-fat diet (HFD, Supplementary Table 1) consumption during adolescence on the colonic EC compartment, two independent cohorts of 7-week-old mice were fed a high-caloric HFD or a corresponding control diet (CD, Supplementary Table 1) for either 6 or 12 weeks. To frame the findings within the context of eoCRC in humans, we calculated the corresponding equivalent human age of the mice using a formula from Wang et al. [28] (Fig. 1a). At the start of the experiment, mice were in early adolescence, corresponding to a human age of 13-14 years. After 6 weeks, they reached late adolescence, equivalent to 21-22 years in humans, and after 12 weeks of intervention, they reached young adulthood, equivalent to a human age of 25 years (Fig. 1a). As anticipated, HFD-fed mice exhibited significantly greater weight gain compared to their CD littermates in both experiments (Fig. 1b, c). Furthermore, the 12-week HFD-fed mice displayed significantly greater final weight gain than the 6-week group, resulting in a more pronounced overweight phenotype (Fig. 1d). After 6 weeks of dietary intervention, a HFD-induced metabolic phenotype was evident in serum 1H-Nuclear Magnetic Resonance (NMR) profiles, as reflected by the distinct clustering of CD- and HFD-derived metabolites in the principal component analysis (Fig. 1e, a full list of analysed metabolites provided in Supplementary Table 2). In detail, acetic acid (*p*-value = 0.0022, mean-rank diff. = −6.00), lactic acid (*p*-value = 0.0087, mean-rank diff. = −5.33), and ethanol (*p*-value = 0.0065, mean-rank diff. = −5.50) were significantly downregulated (Fig. 1f, Supplementary Table 2). HFD-induced metabolites comprised increased levels of cholesterol (*p*-value = 0.0022, mean-rank diff. = 6.00) and glucose (*p*-value = 0.0065, mean-rank diff. = 5.67) (Fig. 1f, Supplementary Table 2). No differences were detected between both groups regarding analysed amino acids (Fig. 1f, Supplementary Table 2). After both independent HFD-interventions, mice displayed a significant hyperglycaemic phenotype (median serum D-glucose increase 1.2-fold, 6 weeks; 1.3-fold, 12 weeks), compared to the corresponding CD-fed animals (Fig. 1g). However, while serum levels of insulin and C-peptide were not altered after 6 weeks of HFD feeding (Fig. 1h, left panel), mice exhibited elevated insulin plasma levels as well as systemic insulin resistance after 12-week HFD consumption [29] (Fig. 1h, right panel). The insulin receptor (INSR) signalling cascade primarily involves the canonical PI3 kinase/AKT pathway, which promotes anabolic processes, such as glucose uptake, protein synthesis, glycogen synthesis, lipid synthesis, and proliferation [30] (Fig. 1i). Consistent with the hyperglycaemia observed in HFD-fed mice, colonic *Insr* expression was significantly upregulated after 6 weeks of HFD, whereas a trend towards downregulation was detected after 12 weeks compared to CD controls (Fig. 1j). Corroborating our results on transcriptional expression after 6 weeks, INSRß levels were significantly elevated, but not in 12-week HFD-fed mice (Fig. 1k). Of particular note, relative serum glucose concentrations remain comparable between 6 and 12 weeks of HFD, in contrast, INSRβ abundance in colonic tissue of HFD-fed mice is markedly reduced at 12 weeks relative to 6 weeks (Fig. 1g, k). To functionally study the impact of altered INSR abundance we examined the phosphorylation status of the key downstream mediators AKT and AMPKα to assess alterations in INSR signalling pathway activity. While AKT phosphorylation was not affected, phosphorylation of AMPKα was significantly enhanced in the colonic tissue of 6-week HFD-fed mice (Fig. 1l), indicating the occurrence of colonic energy deficiency [31]. After 12 weeks of HFD feeding, colonic INSR level were associated with significantly reduced AKT phosphorylation, but no alterations of AMPKα activation (Fig. 1m). A similar, however more time-dependent decline in AMPKα phosphorylation has previously been reported in a mouse model of HFD-induced insulin resistance, reflecting the opposing roles of AMPKα in energy conservation and AKT in energy-demanding anabolic growth (Fig. 1i) [32]. In particular, the downregulation of AKT phosphorylation in conjunction with elevated systemic insulin concentrations supports the hypothesis of the development of an insulin-resistant-like phenotype in colonic ECs. In addition to INSR downstream mediators, we assessed expression of key glycolytic enzymes. After 6 weeks of HFD feeding, colonic pyruvate kinase isozymes M (*Pkm*), catalysing the last step of glycolysis by dephosphorylating phosphoenolpyruvate to pyruvate and lactate dehydrogenase A (*Ldha*) were not regulated. The pyruvate dehydrogenase (*Pdh*), catalysing the conversion of pyruvate into acetyl-CoA was not detected in the samples (Fig. 1n). Notably, decreased serum lactate levels together with hyperglycemia (Fig. 1f, g) following the 6-week intervention indicate high systemic glucose availability, while intracellular glucose flux might be already limited. Despite the absence of insulin resistance, glucose uptake could be suppressed. Interestingly, Boden et al. identified elevated FA availability resulting from HFD as key driver of glucose uptake inhibition [33]. At 12 weeks, the observed trend towards reduced *Pkm* and *Pdh* expression, together with unchanged *Ldha* levels (Fig. 1n), suggesting a shift in glycolytic capacity at the transcriptional level. In combination with the above-described alterations in colonic epithelial insulin receptor signalling, these findings are consistent with reduced glycolytic flux of colonic ECs and align with impaired insulin signalling. Collectively, our results reveal that the 6-week HFD exposure in mice during adolescence disrupts glucose uptake prior to the onset of insulin resistance, whereas 12-week HFD consumption induced both systemic insulin resistance and impaired insulin sensitivity in colonic ECs, leading to reduced glycolytic flux in these cells and a phenotype consistent with a colonic EC-specific insulin resistance-like phenotype.

### Young adult mice with a colonic EC insulin resistance-like phenotype display an inflamed and hyperproliferative colonic epithelium

**Fig. 2:**
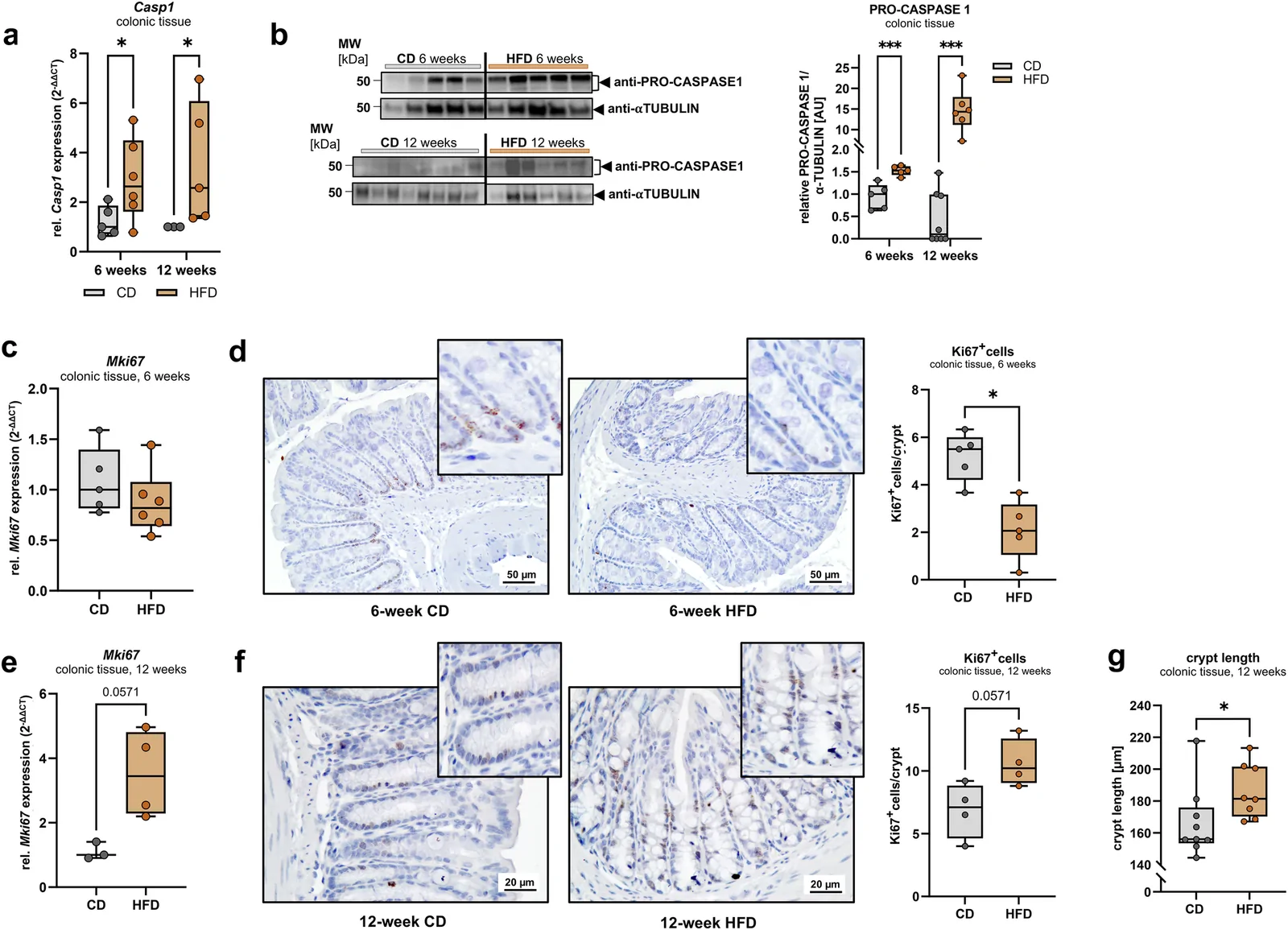
Colonic epithelial insulin resistance in young adult mice is associated with inflammation and hyperproliferation. **a** Relative expression of caspase-1 mRNA (*Casp1)* in murine colonic tissue after 6-week (HFD *n *= 6, CD *n *= 5) and 12-week (HFD *n *= 5, CD *n *= 3) intervention measured by RT-qPCR. **b** Left: Detection of colonic CASP1 level in mice after 6-week (HFD *n *= 5, CD *n *= 5) and 12-week (HFD *n *= 6, CD *n *= 8) dietary intervention using Western blotting. Right: Quantification of colonic CASP1 in HFD-fed mice (6-week *n *= 5; 12-week *n *= 6) and CD-fed mice (6-week *n *= 5, 12-week *n *= 8). **c** Relative expression of *Mki67* in murine colonic tissue after 6 weeks of dietary intervention (HFD *n *= 6, CD *n *= 5) measured by RT-qPCR. **d** Left: Representative images of IHC staining of murine colonic Ki67 after 6-week dietary intervention. Right: Total epithelial Ki67-staining in the colonic tissue of mice after 6-week dietary intervention quantified and normalised to crypt count (HFD *n *= 5, CD *n *= 5). **e** Relative expression of *Mki67* in murine colonic tissue after 12-week dietary intervention (HFD *n *= 4, CD *n *= 3) measured by RT-qPCR. **f** Left: Representative images of IHC staining of colonic Ki67 after 12 weeks dietary intervention. Right: Total epithelial Ki67 staining in colonic tissue quantified and normalised to crypt count (HFD *n *= 4, CD *n *= 4). **g** Colonic crypt length in mice measured from H&E-stained sections. **a**, **b** Two-way ANOVA with Šidák’s multiple comparisons test; **c**–**g** Mann-Whitney *U* test. Box plots depict the median and IQR with whiskers indicating the range from at least three different mice. All relative values were determined by normalisation to the median of the respective control group (set to 1). *P ≤ 0.05, ***P ≤ 0.001. IQR, interquartile range.

Earlier work by Dupaul-Chicoine et al. demonstrated that the inflammatory caspase-1 (CASP1) is essential for colonic EC proliferation and tissue repair under inflammatory conditions, thereby playing a pivotal role in preserving intestinal homeostasis [34]. To study the capacity of HFD consumption to trigger intestinal inflammation, caspase-1 was quantified on mRNA and protein levels in colonic tissue. In response to HFD, a significant upregulation of *Casp1* was observed in both interventions (Fig. 2a). Alongside, a marked increase in CASP1 protein levels was evident after 6 weeks (median 1.53-fold increase), while the same phenotype became more pronounced after 12 weeks of HFD intervention (median 14.75-fold increase) (Fig. 2b). Remarkably, as indicated by previous findings [29], no macroscopically visible inflammation was detected after 12 weeks of HFD feeding. Complementarily, we recently published that *CASP1* expression positively correlated with *KI67* expression in human CRC tissues [35]. After 6 weeks of HFD feeding, increased CASP1 abundance did not affect *Mki67* expression (Fig. 2c), while in line with the observed colonic energy deficiency after 6 weeks of HFD, quantification of immunohistochemical staining (IHC) of colonic crypts revealed a significantly decreased abundance of total Ki67^+^ epithelial cells (Fig. 2d). In contrast, mice displayed a strongly increased *Mki67* expression after 12 weeks of HFD (Fig. 2e) which was validated by higher numbers of total Ki67^+^ epithelial cells in IHC staining’s (Fig. 2f). Hyperproliferation often leads to an elongation of the crypts, as more cells are produced and migrate along the crypt axis [36]. Therefore, colonic crypt length analysis constitutes a standardized marker for the determination of intestinal proliferative activity. Colonic hyperproliferation was accompanied by significantly elongated crypt length in mice after 12 weeks of HFD (HFD median length: 181.000 µm *vs*. CD median length: 156.008 µm, *p* = 0.0360) (Fig. 2g). This further supports the hypothesis that increased colonic inflammation, in concert with a colonic EC insulin resistance-like state, favours CASP1-mediated proliferation of intestinal ECs.

### Excessive colonic ω6-PUFA metabolism signalling promotes triglyceride metabolism in WD-related insulin-resistant mice

**Fig. 3:**
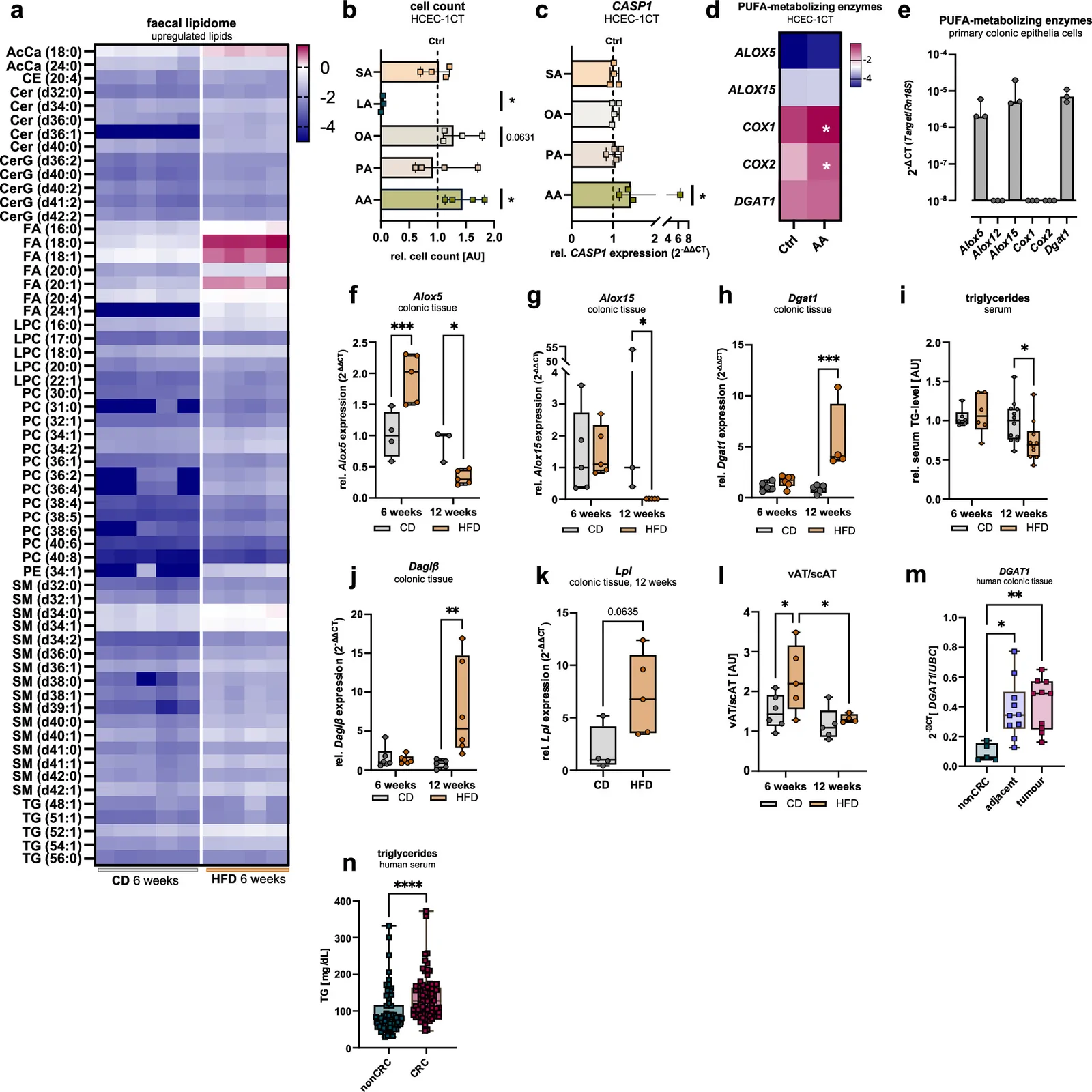
Insulin-resistant colonic epithelium redirects ω6-PUFA metabolism toward *Dgat1*- and *Daglβ*-dependent triglyceride turnover. **a** Significantly upregulated lipids in faecal samples of mice after 6-week HFD (*n *= 4 mice) in comparison to CD (*n *= 5 mice). **b** Relative cell count of the human non-cancerous cell line HCEC-1CT stimulated with 10 µM SA, OA, PA, AA (each group *n *= 4) or LA (*n *= 3), and compared to the respective control conditions set to 1 (Ctrl; ≤0.1% (v/v) EtOH, H₂O, or DMSO; each *n *= 4). **c** Relative *CASP1* expression of FA-treated HCEC-1CT (each group *n *= 4) in comparison to the respective control treatment (Ctrl). **d** Expression of PUFA-metabolizing enzymes in Ctrl-treated (*n *= 5) and AA-treated (*n *= 5) HCEC-1CT quantified using RT-qPCR and depicted as log-transformed 2^-∆CT^. **e** PUFA-metabolising enzyme expression in primary colonic epithelial cells (pCECs) from mice (*n *= 3 mice). Relative colonic expression of **f** *Alox5* (6 weeks: HFD *n *= 5 mice, CD *n *= 4 mice; 12 weeks: HFD *n *= 5 mice, CD *n *= 3 mice), **g** *Alox15* (6 weeks: HFD *n *= 5 mice, CD *n *= 5 mice; 12 weeks: HFD *n *= 5 mice, CD *n *= 3 mice), and **h** *Dgat1* (6 weeks: HFD *n *= 6 mice, CD *n *= 5 mice; 12 weeks: HFD *n *= 4 mice, CD *n *= 5 mice) in mice after 6- and 12-week HFD intervention. **i** Relative serum triglyceride concentration in murine serum after 6-week (HFD *n *= 6 mice; CD *n *= 6 mice) and 12-week (HFD *n *= 10 mice; CD *n *= 12 mice) intervention. **j** Relative colonic expression of *Daglβ* after 6-week (HFD *n *= 6 mice, CD *n *= 6 mice) or 12-week (HFD *n *= 6 mice, CD *n *= 5 mice) intervention period. **k** Relative *Lpl* expression after 12-week intervention (HFD *n *= 5 mice, CD *n *= 4 mice). **l** Visceral fat (vAT) to subcutaneous fat (scAT) ratio after 6-week (HFD *n *= 5 mice, CD *n *= 6 mice) and 12-week (HFD *n *= 4 mice, CD *n *= 5 mice) nutritional intervention. **m** Expression pattern of *DGAT1* in age-matched human colonic biopsies from hospitalized normal patients (nonCRC, *n *= 5 different patients) and non-cancerous tissue adjacent to tumour side (adjacent, *n *= 10 different patients) and paired tumour tissue (tumour, *n *= 10 different patients) from Origene. **n** Quantification of triglyceride level in human serum of patients with CRC (CRC, *n *= 79 different patients) compared to nonCRC (*n *= 61 different patients), determined using NMR *IVDr* metabolomics. **a**–**c**, **f**–**j**, **l** Two-way ANOVA with Fisher’s least significant difference (LSD) test. **d**, **k**, **n** (Multiple) Mann-Whitney U test. **m** Kruskal-Wallis test with Dunn’s multiple comparisons test. Heatmap values in **a** shown as log-transformed individual data point [AU] and **d** values shown as median. **b**, **c**, **e** Bar graphs shown as median with error bars indicating the range. **f**–**n** Box plots depict the median and IQR with whiskers indicating the range. **e**, **b**–**d** Data from independent biological replicates and **a**, **e**, **f**–**l** from at least three different mice. **b**, **c** Relative values were calculated by normalising to the paired control condition (set to 1). **f**–**k** Relative values were determined by normalisation to the median of the respective control group (set to 1). *P ≤ 0.05, **P ≤ 0.01, ***P ≤ 0.001, ****P ≤ 0.0001. AA arachidonic acid, CD control diet, Ctrl control, CRC colorectal cancer, FA fatty acid, HFD high-fat diet, IQR interquartile range, LA linoleic acid, OA oleic acid, PA palmitic acids, SA stearic acid.

Elevated levels of FAs, resulting from diet or derived from bacteria, can activate the NLRP3 inflammasome, resulting in CASP1 activation, and therefore elicit an inflammatory response [34, 37, 38]. PUFA metabolism in particular has been associated with intestinal inflammation, as excessive dietary intake of PUFAs triggers endoplasmic reticulum stress and activates TLR2 signalling [14]. Further, diet-related luminal FAs have been demonstrated to directly interact with colonic ECs [39]. Hence, we performed a Liquid Chromatography-Mass Spectrometry- (LC/MS) based lipidomic analysis to study the impact of 6-week HFD consumption on faecal lipidome profiles. While 58% of the faecal lipid molecules were significantly upregulated, only 9% lipid molecules were significantly downregulated in HFD-fed mice (Fig. 3a, Supplementary Fig. 1a, Supplementary Table 3). Among the significantly upregulated lipid metabolites, the following candidates displayed high abundances: acylcarnitine (AcCA 18:9; AcCA 24:0), ceramide (Cer d34:0; Cer d34:0; Cer d36:0; Cer d40:0; Cer d36:1), glycosphingolipid (CerG d36:2; CerG d40:0; CerG d40:1; CerG d40:2; CerG 42:2), lysophosphatidylcholine (LPC 16:0; LPC 17:0; LPC 18:0; LPC 20:0; LPC 22:1), phosphatidylcholine (PC 30:0; PC 31:0; PC 32:1; PC 34:1: PC 36:1, PC 36:2; PC 36:4; PC 38:4; PC 38:5; PC 38:6, PC 40:6; PC 40:8), sphingomyelin (SM d32:0; SM d34:0; SM d36:0; SM d36:1; SM d38:0; SM d38:1; SM d39:1; SM d40:0; SM d40:1; SM d41:0; SM d42:0; SM d42:1), and triacylglycerols encompassing mostly saturated or MUFAs (TAG 48:1; TAG 51:1; TAG 52:1; TAG 54:1; TAG 56:0) (Fig. 3a). Notably, among these upregulated lipid metabolites, free FAs exhibited the greatest upregulation: palmitic acid (PA; C16:0), stearic acid (SA; C18:0), oleic acid (OA; C18:1), arachidic acid (C20:0) and eicosenoic acid (C20:1) (Fig. 3a). Additionally, these findings indicate that the colonic epithelium is exposed to excessive FA load, further supporting the hypothesis that glucose uptake is potentially inhibited by FA overload. Most outstanding, the essential ω6-PUFA AA (C20:4) was strongly upregulated in faecal samples from 6-week HFD-fed mice compared to CD-fed mice (Fig. 3a). Interestingly, AA was recently unravelled to promote intestinal inflammation and inflammation-driven CRC development [14, 40, 41]. To assess the capacity of FAs to trigger hyperproliferation in the human context, as observed in 12-week HFD-fed mice, the non-cancerous human colonic epithelial cell line HCEC-1CT was cultivated in the presence or absence of HFD-related FAs, including AA, PA, LA, OA, or SA, for five days. Only OA and AA significantly increased HCEC-1CT cell proliferation, with AA stimulation demonstrating the strongest effect (Fig. 3b). Of note, due to its high content in the applied HFD, LA (C18:2) was also tested in vitro, although it was not upregulated in faecal samples collected from HFD-fed mice. However, LA inhibited cell proliferation in HCEC-1CT cells (Fig. 3b). Furthermore, AA exclusively induced an upregulation of *CASP1* expression (Fig. 3c) but did not affect *INSR* expression (Supplementary Fig. 1b). The absence of direct transcriptional regulation of INSR suggests that AA-mediated epithelial reprogramming in this model might not occur through altered receptor abundance itself, but rather through downstream modulation of insulin signalling pathways, or post-translational mechanisms affecting insulin responsiveness. Furthermore, it should be noted that experiments using the HCEC-1CT cell line were conducted over a comparatively short treatment period relative to the in vivo models. Therefore, potential effects on insulin receptor expression itself may not yet be detectable at these early stages of stimulation. Given that AA uniquely mimics most of the key features of the colonic EC phenotype observed in mice after 12-week HFD-intervention, its mode of action in colonic ECs was further investigated. AA can be metabolised via two primary pathways: the cyclooxygenase pathway and the lipoxygenase pathway. COX are the key enzymes in the cyclooxygenase pathway, yielding pro-inflammatory prostaglandins as downstream products, whereas the lipoxygenase pathway is driven by ALOX enzymes, with ALOX5 as the rate-limiting enzyme, leading to leukotriene production [42, 43]. Stimulation of HCEC-1CT cells with AA in vitro significantly upregulated *COX1* and *COX2*, while *ALOX5* tended to be upregulated (*p* = 0.095), however, the expression of *ALOX15* was unaffected (Fig. 3d, Supplementary Fig. 1c). Stimulation with PA, SA, or OA did not result in significant changes in the expression of *COX* or *ALOX* enzymes compared to control stimulation (Supplementary Fig. 1d). The absence of significant *COX* or *ALOX* induction following PA, SA, or OA stimulation further suggests that activation of inflammatory lipid signalling pathways in colonic ECs is not a general consequence of FA overload but may instead depend specifically on ω6-PUFA-derived signalling mechanisms mediated by AA. However, expression analysis in primary murine colonic epithelial cells (pCECs) revealed *Alox5*, *Alox15*, and diacylglycerol O-acyltransferase 1 (*Dgat1*), a key enzyme in triglyceride (TAG) synthesis and energy storage, to be highly expressed (Fig. 3e). Whereas *Alox12*, *Cox1*, and *Cox2* were almost undetectable (Fig. 3e). Based on this basal expression profile, we focused subsequent analyses on the enzymes that were predominantly expressed. After 6-week HFD consumption, only *Alox5* expression was significantly upregulated, while *Alox15*, *Cox1*, and *Cox2*, remained unchanged compared to the CD-fed mice (Fig. 3f, g, Supplementary Fig. 1e, f). The absence of *Cox1*/*2* induction suggests that early HFD-induced epithelial adaptation is not primarily mediated through classical prostaglandin-driven inflammatory responses. Similarly, unchanged Alox15 expression may indicate selective activation of distinct ω6-PUFA metabolic branches, favouring leukotriene-associated signalling rather than broad activation of lipid inflammatory pathways. In contrast, after 12 weeks of HFD consumption, colonic *Alox5* was significantly downregulated, while *Alox15* expression tended to be downregulated compared to CD-fed mice (Fig. 3f, g), indicative of a switch away from ALOX signalling. On the other hand, expression of enzymes of the COX pathway remained unchanged (Supplementary Fig. 1e, f). It is known that an excessive amount of exogenous FAs induces DGAT1, which exhibits enhanced TAG biosynthetic activity under HFD conditions [44, 45]. Interestingly, stimulation of HCEC-1CT cells with the HFD-related FAs AA, PA, LA, OA, or SA did not alter the expression of *DGAT1* (Fig. 3d, Supplementary Fig. 1d). This suggests that induction of epithelial lipid-buffering programmes may require chronic metabolic stress, combinatorial nutrient exposure, or systemic factors present in vivo rather than acute exposure to single FA species alone. In the 6-week HFD mouse model, *Dgat1* expression remained unchanged compared to CD-fed controls, which corresponded to unchanged serum TAG levels at this time point (Fig. 3h, i). In contrast, after 12-week HFD feeding *Dgat1* was significantly upregulated (Fig. 3h). Intriguingly, serum TAG levels were reduced in HFD compared to CD mice (Fig. 3i). One possible explanation could be enhanced intracellular diacylglycerol lipase beta (DAGLβ) activity, reflected by the significantly increased colonic *Daglβ* expression (Fig. 3j), which has recently been reported to exhibit PUFA-specific TAG lipase activity [46]. Additionally, colonic Lipoprotein lipase (*Lpl)* expression was strongly increased in colonic insulin resistant-like ECs of mice (Fig. 3k), thereby reflecting accelerated TAG hydrolysis and faster clearance of circulating TAGs, leading to increased FA uptake by colonic ECs. Notably, the high proportion of absorbed FAs may, in part, reflect the hyperproliferative state detected in the insulin resistant mice, which increases a demand for lipids for membrane biosynthesis. In parallel, reduced serum TAG levels and high FA load suggest enhanced intracellular lipid metabolism, which could be reflected by the decreased visceral-to-subcutaneous adipose tissue (vAT/scAT) ratio (Fig. 3l), as fewer lipids are stored in vAT depots. Increased lipid flux is known to favour mitochondrial FA oxidation [47], suggesting a metabolic switch towards β-oxidation as cellular energy source in the insulin resistant colonic ECs. In the context of CRC progression, a comparison of age-matched healthy colon tissue with paired tumour-adjacent tissue and CRC tissue revealed similarly a significant upregulation of *DGAT1* in both tumour-adjacent and tumour tissue relative to healthy controls (Fig. 3m). This increased *DGAT1* expression is associated with elevated serum TAG concentrations in CRC patients compared to nonCRC patients (Fig. 3n, Supplementary Tables 4, 5), suggesting a role for DGAT1 in the metabolic alterations associated with tumorigenesis. Of note, the nonCRC cohort was relatively small and no clinical information regarding dietary status or Body-Mass-Index (BMI) were available. Further validation in larger, well-characterised cohorts will be required, particularly as DGAT1 expression may be influenced by inter-individual differences in lipid metabolism, metabolic status, medication use, and other clinical factors. Together, these findings indicate enhanced colonic ω6-PUFA metabolism via ALOX enzymes in mice after 6 weeks of HFD, which becomes attenuated in the colonic EC insulin-resistant mice. In compensation, the upregulation of colonic *Lpl*, *Dgat1*, and *Daglβ* could point towards the establishment of a high-turnover lipid cycle characterised by enhanced FA uptake, transient triglyceride storage, and subsequent remobilisation. While our data do not directly assess lipid flux, this interpretation is supported by literature demonstrating the role of LPL in triglyceride uptake into the tissue, DGAT1 in triglyceride buffering, and DAGLβ in PUFA-containing triglyceride hydrolysis [46, 48, 49]. Additionally, Maan et al. summarize that tumour cells have a high FA turnover to meet their energetic requirements and that altered FA metabolism plays a central role in tumour development [50].

### Excessive colonic ω6-PUFA metabolism inhibits glucose uptake at the expense of LGR5^+^  ISC proliferation, but drives qISC expansion

**Fig. 4:**
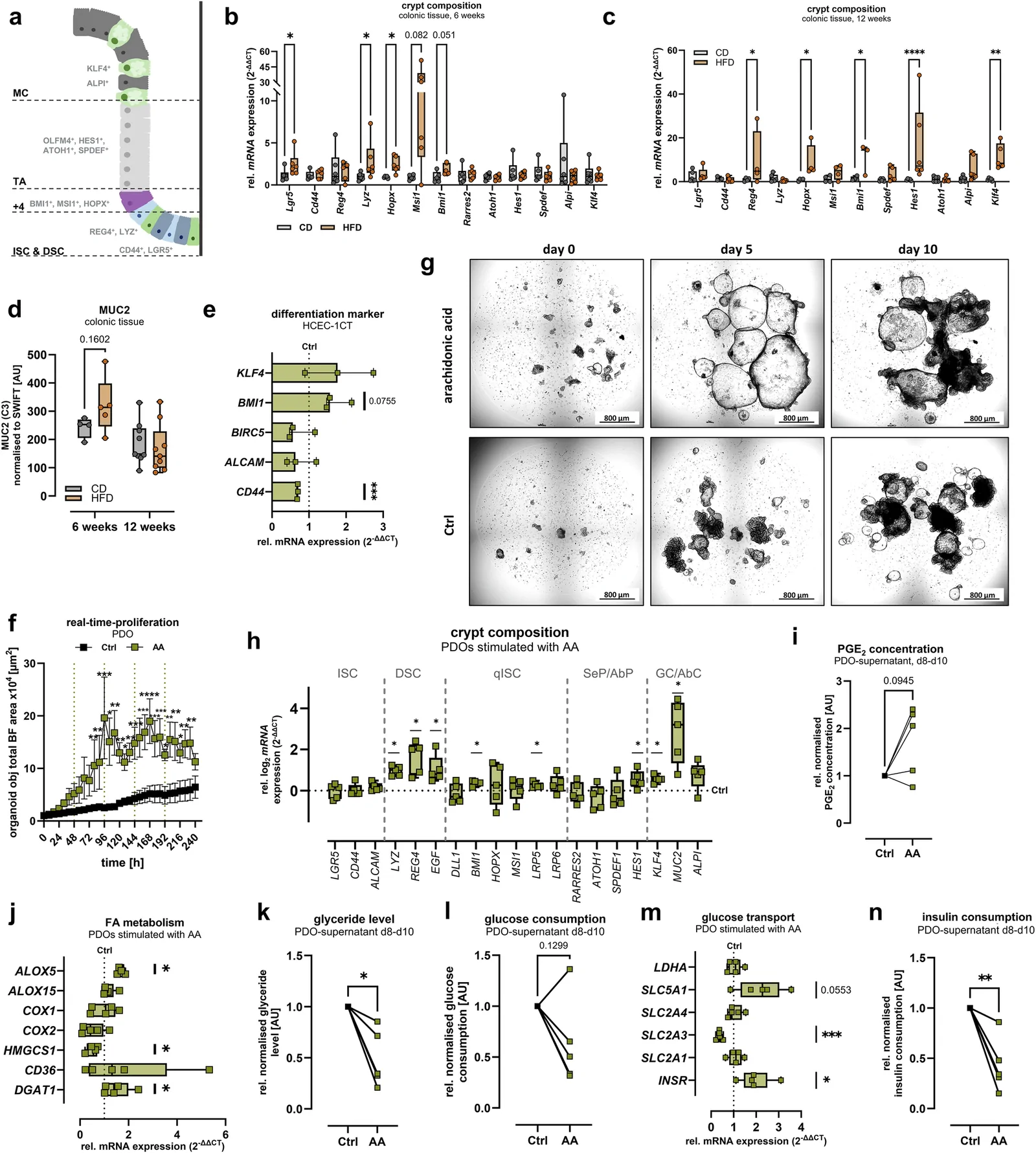
ω6-PUFA overload limits glucose uptake and LGR5^+^ ISC proliferation while expanding qISCs. **a** Illustration of the colonic crypt composition. **b** Relative expression of colonic crypt markers in colonic tissue of mice after 6-week HFD (*n *= 5 mice for *Reg4*, *Atoh1*, *Hes1* or *n *= 6) compared to CD (*n *= 5 mice for *Cd44*, *Hopx*, *Msi1*, *Bmi1*, *Atoh1* or *n *= 6). **c** Expressional comparison of colonic crypt markers in colonic tissue of mice between HFD (*n *= 3 mice for *Bmi1*; *n *= 4 for *Reg4*, *Hopx*, *Msi1*; *n *= 5 for *Lgr5*, *Cd44*, *Klf4*; *n *= 6 for *Lyz*, *Spdef*, *Hes1*, *Atoh1*, *Alpi*) and CD (*n *= 4 for *Cd44*, *Reg4*, *Hopx*, *Msi1*, *Bmi1*; *n *= 5 for *Spdef*, *Hes1*, *Klf4*; *n *= 6 for remaining targets) after 12-week dietary intervention. **d** Normalized quantification of MUC2 in colonic tissue of HFD and CD mice after 6 weeks (HFD *n *= 5, CD *n *= 4) and 12 weeks (each group *n *= 9 mice). **e** Transcriptional differentiation profile of AA-stimulated HCEC-1CT cells (*n *= 3) relative to DMSO-treated controls (Ctrl, *n *= 3). **f** Paired real-time proliferation of human colonic organoids from healthy (nonCRC) patients treated with AA (*n *= 5 independent PDOs) or Ctrl (*n *= 5 independent PDOs), depicting Brightfield Area x 10^4^ [µm²] captured every eight hours, normalised to day 0 with medium exchange indicated by dotted vertical lines. **g** Representative brightfield microscopy images of human colonic organoids at initiation (day 0), on day 5, and on day 10 of AA stimulation or Ctrl (4x magnification). **h** Log₂-transformed relative expression of AA-treated healthy human colonic organoids (*n *= 5 independent PDOs) compared to paired DMSO-treated controls (Ctrl, *n *= 5 independent PDOs). **i** Relative normalized prostaglandin E2 (PGE2) concentration quantified in the supernatant of day 8 to day 10 (d8-d10) from AA-treated (*n *= 5 independent PDOs) and compared to paired DMSO-treated (Ctrl, *n *= 5 independent PDOs) organoids. **j** Expression of different enzymes of the FA-metabolism quantified in AA-treated human colonic organoids from nonCRC patients (*n *= 5 independent PDOs) relative to paired DMSO-treated controls (Ctrl, *n *= 5 independent PDOs) using RT-qPCR. **k** Relative normalised glyceride levels and **l** relative normalised D-glucose consumption were quantified in the supernatant of d8-10 from AA-treated human colonic organoids from nonCRC patients (*n *= 5 independent PDOs) and compared to paired DMSO-treated organoids (Ctrl, *n *= 5 independent PDOs). **m** RT-qPCR analysis of enzymes important for glycolytic activity in AA-treated human colonic organoids (*n *= 5 independent PDOs) relative to paired DMSO-treated controls (Ctrl, *n *= 5 independent PDOs). **n** Relative normalised insulin consumption quantified in the supernatant of d8-d10 from AA-treated human healthy colonic organoids (*n *= 5 independent PDOs) and compared to paired DMSO-treated organoids (Ctrl, *n *= 5 independent PDOs). **b**, **e**, **h**, **j** Multiple Mann-Whitney *U* test with unadjusted *P-*values. **c**, **d**, **f**, **m**, Two-way ANOVA with Fisher’s least significant difference (LSD) test. **i**, **k**, **l**, **n** Paired student’s t-test. **b**–**d**, **h**, **j**, **m** Box plots depict the median and IQR with whiskers indicating the range, **e** data shown as median ± range and **f** as mean ± S.E.M.. Data in **e** from independent biological replicates, **b**–**d** from at least three different mice and **f** five independent PDOs. **b**–**d** Relative values were determined by normalisation to the median of the respective control group (set to 1); **e**, **h**–**n** relative values were calculated by normalising to the paired control condition (set to 1). *P ≤ 0.05, **P ≤ .01, ***P ≤ 0.001, ****P ≤ 0.0001. AbC absorptive enterocyte, AbP absorptive progenitor, Ctrl control, DSC deep secretory cell, FA fatty acid, GC goblet cell, PDO patient-derived organoid, ISC intestinal stem cell, IQR interquartile range, qISC quiescent stem cell, S.E.M standard error of the mean, SeP secretory progenitor.

As illustrated in the schematic representation of the colonic crypt (Fig. 4a), its architecture can be divided into four distinct zones: The lower part of the colonic crypt comprises the active cycling stem cell zone, housing cycling, highly glycolytic LGR5^+^/CD44^+^ ISCs, and REG4^+^/LYZ^+^ deep secretory cells (DSCs). Adjacent to this, the +4-position harbours the BMI1^+^/MSI1^+^/HOPX^+^ qISCs. In a gradual transition, the transient amplifying (TA) zone follows, where HES1^+^/ATOH1^+^/SPDEF1^+^ progenitor cells actively proliferate and migrate upwards within the crypt, towards the mature cell (MC) zone where the fully functional KLF4^+^/MUC2^+^ secretory and ALPI^+^ absorptive cells reside [51]. The cellular composition of the colonic crypt is tightly regulated and shifts in its balance can disrupt crypt homeostasis. To assess whether insulin resistance affects this equilibrium, we analysed the crypt composition in 6-week HFD-fed mice that did not exhibit systemic or colonic insulin resistance. Strikingly, only markers associated with the lower crypt compartment were significantly upregulated in HFD-fed mice compared to CD-fed controls. Specifically, the ISC marker *Lgr5*, the qISC markers *Hopx*, *Bmi1*, and *Msi1*, as well as the DSC marker *Lyz*, showed strong upregulation, while markers of the TA and MC zones were not differentially expressed (Fig. 4b). To determine whether these alterations persist or evolve with the onset of systemic and the colonic EC insulin resistance-like phenotype, we next analysed crypt composition in mice after the 12-week intervention. Increased expression of *Hopx* and *Bmi1* persisted, whereas *Msi1* and *Lgr5* were no longer differentially expressed (Fig. 4c). Instead, markers for secretory ECs such as *Klf4* or *Reg4* were significantly upregulated (Fig. 4c). While *Klf4* upregulation implied an involvement in secretory cell differentiation, the absence of increased colonic MUC2 protein levels suggested that the DSC function rather than goblet cell function was expanded (Fig. 4d, Supplementary Fig. 1g). Additionally, analysis of the *CELLxGENE Explorer* dataset “Tabula Muris Senis, Large Intestine - A single-cell transcriptomic atlas characterizing ageing tissues in the mouse (10x)” [52] revealed that elevated *Klf4* expression may also be attributable to *Lyz*^+^, *Reg4*^+^, *Bmi1*^+^, and *Hes1*^+^ cells, as a notable proportion of these populations co-express *Klf4* (Supplementary Fig. 1h). Within the TA zone, the absorptive progenitor cell marker *Hes1* was significantly upregulated, while markers of mature absorptive cells were not altered (Fig. 4c). Taken together, the data mainly point towards an enrichment of qISC and DSCs. Having established the expression profile in murine colonic epithelium after 6- and 12-week intervention, we validated our findings using the non-cancerous HCEC-1CT. It should be noted that the concentration used was selected based on dose-dependent experiments assessing the expression of the inflammatory markers *CASP*-*1* and *IL1β* (Supplementary Fig. 1i). In reference to the study by Wang et al., employing a concentration of 100 µM [53], we optimized the concentration to 10 µM, as this elicited the strongest effect on the markers described above while also ensuring that the final DMSO concentration in control conditions did not exceed 0.1% (v/v). This was done to minimise any potential confounding effects of DMSO on cellular responses. Based on established markers for HCEC-1CT cells [54], we unravelled AA to alter the analysed EC marker profile (Fig. 4e), whereas OA, LA, and SA had no effects (Supplementary Fig. 1j). In detail, AA treatment led to increased expression of the +4-position marker *BMI1* and significantly decreased expression of the ISC marker *CD44* (Fig. 4e). Given that HCEC-1CT cells represent a simplified colonic EC model, we extended our analysis to ex vivo healthy human colonic 3D-organoids patient-derived organoids (PDOs), which were stimulated with AA or control for a 10-day period. AA exposure enhanced organoid growth, with significantly increased growth from day 3 onwards (Fig. 4f). Moreover, AA stimulation induced a morphological shift, with organoids transitioning from the typical budding morphology towards a more spheroid-like structure (Fig. 4g). These morphological changes are an indication of an altered cellular composition and metabolic shifts, as organoid architecture can be linked to both stem cell abundance and epithelial integrity [55, 56]. Of note, we also investigated whether altered cell polarity or changes in adhesion molecule expression contributed to the observed phenotype. We observed an increase in *EPCAM*, *OCLN*, and *ITGB4* (Supplementary Fig. 1k), suggesting subtle alterations in epithelial organization and cell adhesion rather than a global disruption of epithelial polarity. Importantly, the expression of core epithelial polarity and junctional markers, including *CDH1*, *EZR*, *TJP1* and *CLDN1*, remained unchanged, arguing against overt epithelial disintegration or loss of apical-basal polarity (Supplementary Fig. 1k). Instead, this expression pattern may reflect a modest reinforcement of epithelial cohesion and barrier-associated features. We therefore examined whether AA stimulation affected organoid cell composition and metabolism. AA stimulation had effects on the +4-position and mature secretory cell populations of the colonic organoids, which reflects the findings from the mouse models (Fig. 4h). Of note, a recent study demonstrated that AA activates WNT signalling, possibly stimulating +4-position cells in an LGR5⁺-independent manner [53]. Compared to controls, the +4-position markers *BMI1* (median 1.3-fold), secretory lineage markers such as *MUC2* (median 9.24-fold), *KLF4* (median 1.5-fold), *LYZ* (median 2.0-fold), EGF (median 1.7-fold) or *REG4* (median 4.0-fold), along with the enterocyte marker *ALPI* (median 1.9-fold) were significantly increased (Fig. 4h). Furthermore, analysis of the supernatant of AA-stimulated human organoids between days 8 to 10 revealed strong ω6-PUFA metabolism reflected by increased PGE_2_ secretion (Fig. 4i). Of note, increased PGE_2_ is associated with chronic low-grade inflammation [57]. However, the expression of *CASP1* was not significantly altered by AA compared to control (Supplementary Fig. 1l). Additionally, elevated PGE_2_ secretion suggests the contribution of COX enzymes in the metabolism of AA in human colonic ECs. Although, AA stimulation did not alter *COX1*/*2* expression, elevated PGE_2_ secretion may indicate enhanced COX activity (Fig. 4j). Notably, increased activity of prostaglandin E synthases may also underlie this effect [58]. Furthermore, *ALOX5* and *DGAT1* were upregulated by AA stimulation (Fig. 4j). Interestingly, the main enzyme for cholesterol production *HMGCS1* was significantly downregulated (Fig. 4j). This could align with DGAT’s role in promoting cholesterol uptake, which reduces the need for endogenous cholesterol production [59]. The supernatant of AA-stimulated organoids contained significantly lower concentration of glycerides, including mono-, di- and triglycerides (Fig. 4k). Consistent with the findings in the mouse model, our data suggest that upstream of Dgat1, glycerol-3-phosphate availability might be limited due to reduced glycolytic activity, while FAs are preferentially directed toward β-oxidation. This is supported by the decreased glucose consumption observed in four of five stimulated PDOs (Fig. 4l), pointing towards decreased or inhibited glucose uptake. Additionally, within the glucose metabolism pathway, *INSR* expression was significantly upregulated in colonic PDOs stimulated with AA (Fig. 4m). However, neither the insulin-dependent glucose transporter *SLC2A4* nor the insulin-independent *SLC2A1* were differentially expressed (Fig. 4m). Moreover, glucose transporter *SLC2A3* was significantly downregulated (Fig. 4m). Instead, the sodium-dependent transporter *SLC5A1* showed a strong trend towards upregulation and likely compensates for impaired insulin-dependent glucose uptake (Fig. 4m). Additionally, unchanged *LDHA* expression may point to a shift towards aerobic glycolytic flux (Fig. 4m). Despite increased *INSR* expression, insulin consumption by AA-stimulated organoids was significantly decreased compared to control (Fig. 4n). Together, excessive colonic ω6-PUFA metabolism in insulin sensitive colonic ECs, as observed in 6-week HFD-fed mice, boosted the qISC and DSC compartments. In contrast, reduced ω6-PUFA metabolism in insulin resistant colonic ECs, as observed in 12-week HFD-fed mice, suppressed the LGR5⁺ stem cell pool while promoting expansion of TA cells, qISC and DSC populations. Furthermore, excessive extracellular AA load is highly likely to contribute to impaired glucose uptake and thereby long-term to reduce insulin sensitivity.

### ALOX15 deficiency impairs the active *Lgr5*^+^ ISC compartment and promotes compensatory expansion of *Hopx*^+^ cells and niche-forming *Reg4*^+^ cells

**Fig. 5:**
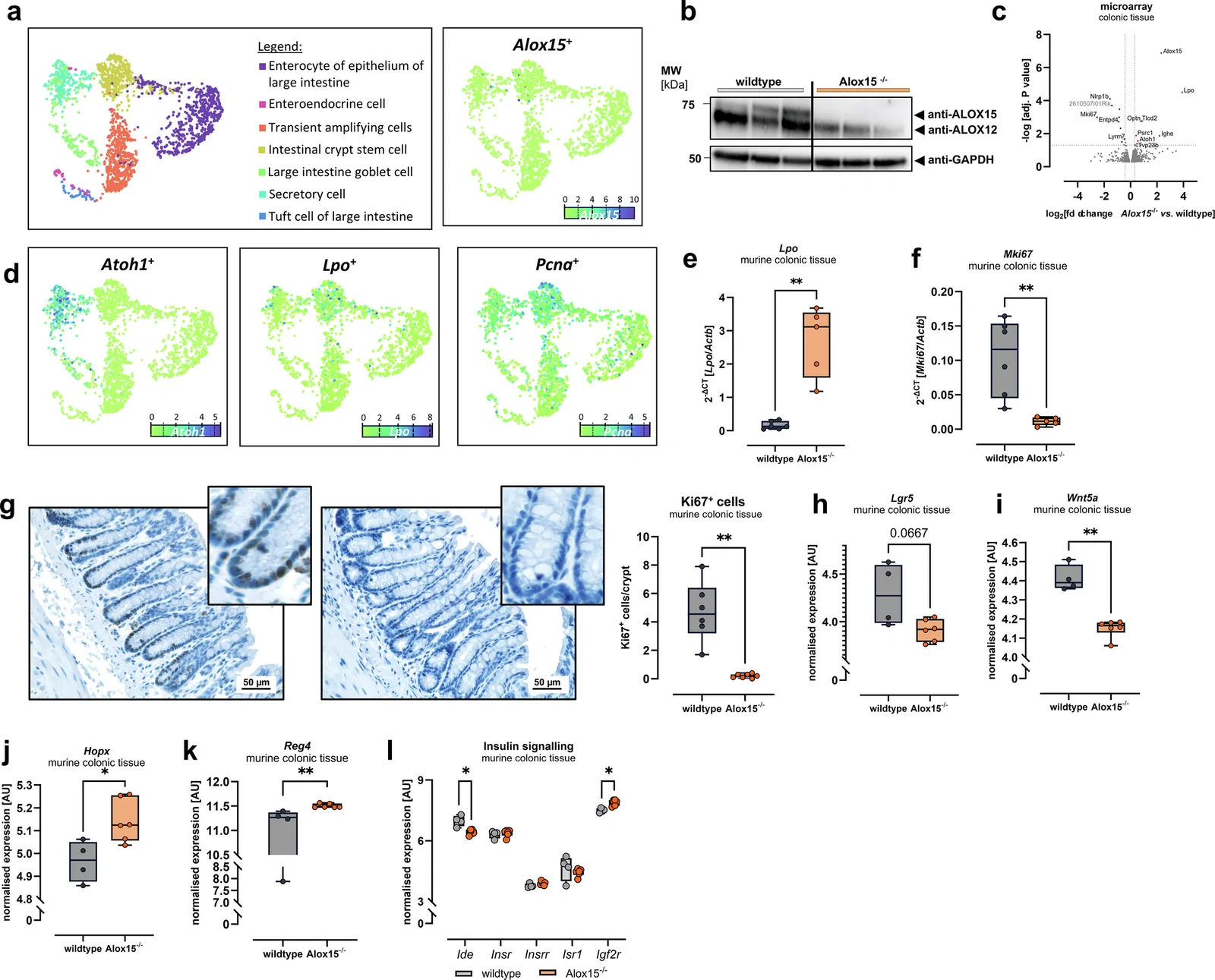
ALOX15 deficiency impairs the active *Lgr5*^+^ ISC compartment and drives compensatory expansion of *Hopx*^+^ cells and *Reg4*^+^ niche cells. **a** Cell type annotation and *Alox15*^*+*^ expression profile in colonic single-cell transcriptomics data of colonic tissue from 3-months old male mice. Single-cell transcriptomics dataset of colonic healthy mouse tissue publicly available at *CELLxGENE Explorer* dataset “Tabula Muris Senis, Large Intestine - A single-cell transcriptomic atlas characterizing ageing tissues in the mouse (10x)”. **b** Validation of ALOX15 loss in colonic tissue of Alox15^-/-^ mice (*n *= 3) compared to wildtype mice (*n *= 3) using Western blot analysis. **c** Vulcano plot showing differential expression analysis of Alox15^-/-^ mice (*n *= 6) and wildtype controls (*n *= 4). **d** *Atoh1*, *Lpo*, and *Pcna* expression in colonic single-cell transcriptomics data of colonic tissue from 3-months old male mice. Single-cell transcriptomics dataset of colonic healthy mouse tissue publicly available at *CELLxGENE Explorer* dataset “Tabula Muris Senis, Large Intestine - A single-cell transcriptomic atlas characterizing ageing tissues in the mouse (10x)”. **e** Validation of increased *Lpo* expression in colonic tissue of Alox15^-/-^ mice (*n *= 5) compared to wildtype mice (*n *= 6) using RT-qPCR. **f** *Mki67* expression analysis via RT-qPCR comparing wildtype mice (*n *= 6) with Alox15^-/-^ mice (*n *= 5). **g** Left: Representative Ki67 staining of colonic murine tissue from wildtype and Alox15^-/-^ mice. Right: Quantification of Ki67⁺ cells per crypt (11-37 crypts per animal). Mean values from *n *= 6 wildtype and *n *= 6 Alox15^-/-^ mice were used for statistical analysis. Normalised expression from microarray analysis of **h** *Lgr5*, **i** *Wnt5a*, **j** *Hopx*, **k** *Reg4*, and **l** transcripts important for insulin signalling (*Ide*, *Insr*, *Insrr*, *Isr1*, *Igf2r*) of wildtype (*n *= 4 mice for each analysis) and Alox15^-/-^ (*n *= 6 mice for each analysis) mice. **c** Differentially expressed genes were identified using a fold-change threshold of |log₂FC | ≥ 0.3 and ≤ -0.4, with multiple testing correction applied using the Benjamini-Hochberg false discovery rate (FDR) method. **e**–**k** Mann-Whitney U test. **l** Mann-Whitney U test using Holm-Šidák method correcting for multiple testing. **e**–**l** Box plots depict the median and IQR with whiskers indicating the range from at least three different mice. *P ≤ 0.05, **P ≤ 0.01.

To gain a better understanding of the role of ALOX enzymes in insulin resistance and qISC expansion, we first interrogated the *CELLxGENE Explorer* dataset “Tabula Muris Senis, Large Intestine - A single-cell transcriptomic atlas characterising ageing tissues in the mouse (10x)” [52]. This analysis revealed that *Alox15* expression was largely restricted to the ISC compartment (Fig. 5a). Based on these observations, we hypothesised that ALOX15 plays an important role in ISC biology and cellular metabolism. To investigate this further, we analysed colonic tissue from basal, non-intervention Alox15^-/-^ and wildtype mice. As expected, ALOX15 protein was undetectable in colonic tissue from Alox15^-/-^ mice, confirming effective gene deletion (Fig. 5b). Next, differential transcriptomic analysis of the colonic tissue revealed significantly reduced expression of the proliferation marker *Mki67*, the transcript of the ectonucleoside triphosphate diphosphohydrolase 4 (*Entpd4*), which hydrolyses metabolic by-products within lysosomes to salvage nucleotides and has also been implicated in the regulation of non-classical apoptotic cell death [60], the inflammasome component *Nlrp1b*, and *Lyrm7*, a transcript encoding for a protein required for the final stages of mitochondrial complex III assembly [61] (Fig. 5c). In contrast, significantly increased expression of optineurin (*Optn*), a multifunctional protein involved in autophagy, membrane trafficking and innate immune responses [62], TRAM-Lag1p-CLN8 domain containing 2 (*Tlcd2*), which regulates membrane composition, lipid distribution and membrane fluidity [63], and proline and serine rich coiled-coil 1 (*Psrc1*), a tumour suppressor encoding a microtubule-associated protein essential for cell division and chromosome segregation [64] (Fig. 5c). Of note, besides strong upregulation of the *Alox15* transcript as a compensatory transcriptional feedback mechanism induced by deletion of exon 3 of the Alox15 gene, transcripts encoding lactoperoxidase (*Lpo)* and *Atoh1* were also significantly upregulated (Fig. 5c). Single-cell RNA expression patterns revealed that *Lpo* was predominantly expressed in proliferating ISCs (Fig. 5d). *Pcna* was used for visualization as a surrogate proliferation marker due to the lack of *Mki67* data in the utilised dataset, strongly enriched in ISCs and partly in DSCs (Fig. 5d). *Atoh1* was highly expressed in secretory epithelial cell populations (Fig. 5d). Independent RT-qPCR experiments were performed to validate microarray data regarding upregulation of *Lpo* expression (Fig. 5e), as well as downregulation of *Mki67* expression (Fig. 5f). Consistent with these transcriptional findings, immunohistochemical staining demonstrated a significantly decreased number of Ki67-positive cells within colonic crypts of Alox15^-/-^ mice compared with wildtype controls (Fig. 5g), indicating reduced proliferative activity. Hence, we next assessed regulation of the *Lgr5* transcript and observed a significant downregulation in Alox15^-/-^ mice (Fig. 5h), highlighting reduction of the proliferative ISC compartment. Additionally, expression of colonic *Wnt5a*, a crucial ligand for receptors on ISC that promote cell proliferation, was significantly decreased (Fig. 5i). Interestingly, expression of *Hopx* (Fig. 5j) and *Reg4* (Fig. 5k) was significantly increased in Alox15^-/-^ mice. Together, these findings point to the crucial role of ALOX15 in cell proliferation of ISC, while the loss of ALOX15 results in loss of cycling ISCs in expense of EC differentiation mainly into DSCs as well as qISCs. Given that ALOX15 acts downstream of ω6-uptake, expression of mediators of the insulin signalling pathway such as *Insr*, insulin receptor-related receptor (*Insrr*), and insulin receptor substrate 1 (*Irs1*) was not affected (Fig. 5l). In contrast, expression of insulin degrading enzyme (*Ide*) was significantly reduced in concert with increased insulin-like growth factor 2 receptor (*Igf2r*) expression, which may point to improved colonic insulin sensitivity in Alox15^-/-^ mice (Fig. 5l).

### Proliferation of LGR5^+^ ISC is driven by INSR signalling, while qISC proliferation depends on growth factor receptor and lipid signalling

**Fig. 6:**
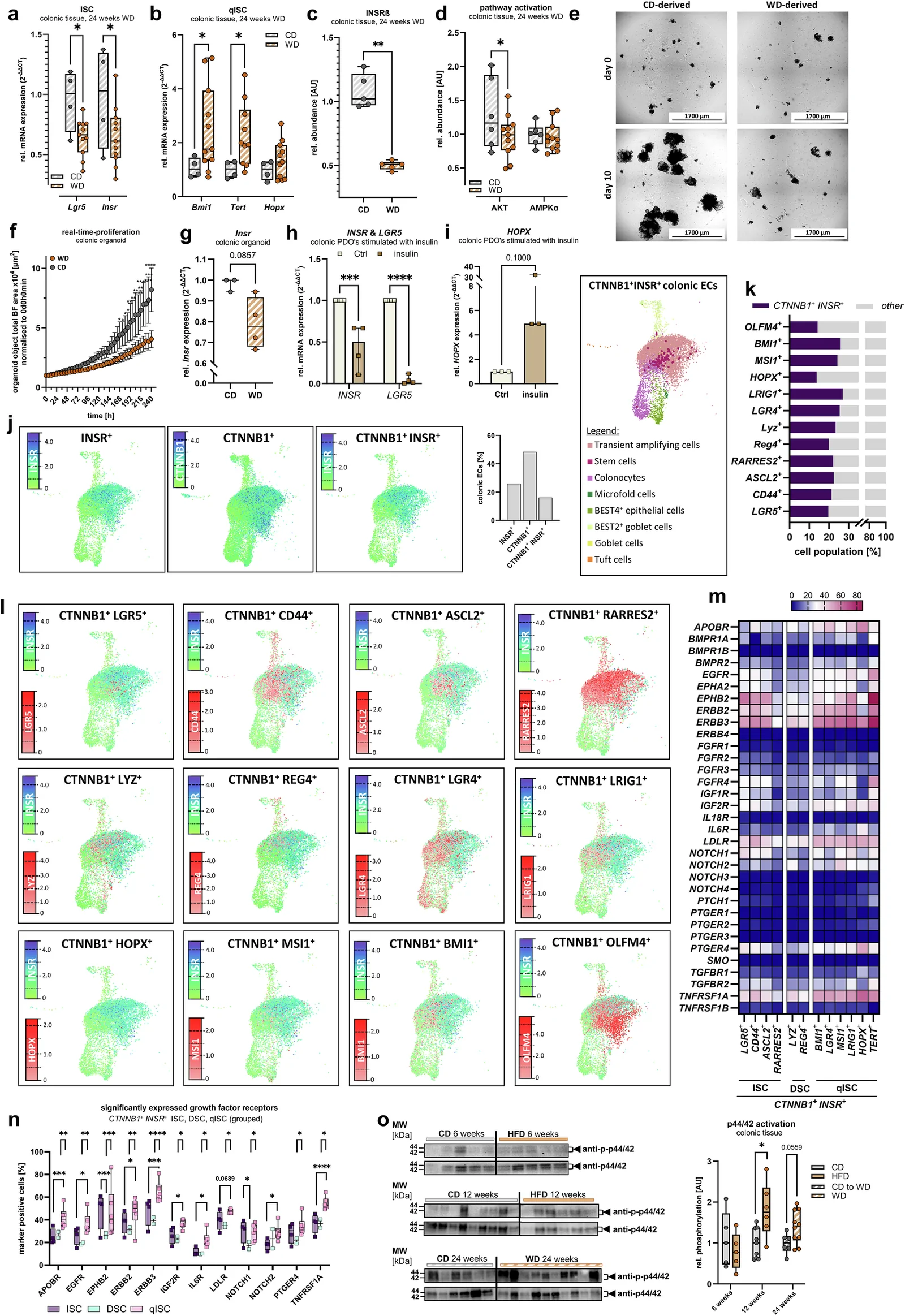
LGR5^+^ ISCs require INSR signalling for proliferation, whereas qISCs depend on growth factor and lipid pathways. **a** Expression of *Lgr5*, *Insr* and **b** *Bmi1*, *Tert*, and *Hopx* quantified in colonic tissue from WD-fed (*n *= 10 for *Tert*, *n *= 11 for remaining targets) relative to CD-fed (*n *= 4) mice using RT-qPCR, displaying unadjusted *P*-values. **c** INSRβ quantification in colonic tissue of WD-mice (*n *= 5) relative to CD-fed controls (*n *= 5) via Western blotting, displaying unadjusted *P*-values. **d** Relative phosphorylation status of AKT, and AMPKα in murine colonic tissue (WD *n *= 11, CD *n *= 6) determined by Western blot analysis. **e** Representative brightfield microscopy images of murine colonic organoids derived from WD- and CD-fed mice at day 0 and day 10 of basal cultivation (4x magnification). Real-time proliferation of WD- (*n *= 4 independent organoids) and CD-derived organoids (*n *= 3 independent organoids), depicting Brightfield Area x 10^4^ [µm²] captured every 6 hours and normalised to day 0. **g** Relative expression of *Insr* in murine WD-derived colonic organoids (*n *= 4 independent organoids) and CD-derived murine colonic organoids (*n *= 3 independent organoids). Relative mRNA expression of **h** *INSR* (Ctrl *n *= 4, insulin-treated *n *= 4) and *LGR5* (Ctrl *n *= 4, insulin-treated *n *= 4), and **i** *HOPX* (Ctrl *n *= 3, insulin-treated *n *= 3) of human colonic PDOs. **j** *INSR* and *CTNNB1* expression profile in single-cell transcriptomics data of colonic tissue from healthy human adults. **k** Proportion [%] of *CTNNB1*⁺*INSR*⁺ cells across lower crypt, based on single-cell transcriptomics data of colonic tissue from healthy human adults. **l** Panels illustrate cells (red scale) expressing ISC-, qISC-, and DSC-specific markers co-expressed within *CTNNB1*^*+*^ and *INSR*^+^ (expression of INSR in green-blue scale), derived from single-cell transcriptomics profiles of healthy human adult colon. **m** Heatmap of single-cell transcriptomics data from healthy human colon showing *CTNNB1*⁺*INSR*⁺ cells expressing ISC-, qISC, DSC-specific markers, and co-expressing a characteristic growth factor receptor signature. **n** Significantly regulated co-expressed markers across human colonic *CTNNB1*^+^*INSR*^+^ epithelial cells of the lower crypt grouped by intestinal stem cell (ISC: *LGR5*, *CD44*, *ASCL2*, *RARRES2*), deep secretory cells (DSC: *LYZ*, *REG4*), and quiescent intestinal stem cells (*BMI1*, *LGR4*, *MSI1*, *LRIG1*, *HOPX*, *TERT*) and based on (**k**). See Supplementary Table 6 for a detailed overview of all statistical analyses performed. **o** Left: Western blot analysis of (phosphorylated) p44/42 (MAPK) after 6 weeks (HFD *n *= 5, CD *n *= 5), 12 weeks (HFD *n *= 9, CD *n *= 9) and after 24 weeks (CD *n *= 6, WD *n *= 10) of dietary intervention. Right: Relative phosphorylation status of MAPK downstream effector p44/42 (6 weeks: HFD *n *= 5, CD *n *= 5 mice; 12 weeks: HFD *n *= 6, CD *n *= 9; 24 weeks: WD *n *= 10, CD *n *= 6), displaying unadjusted *P*-values. **a**, **b**, **d**, **f**, Two-way ANOVA with **a**, **b**, **d**, **f**, **n** Fisher’s least significant difference (LSD) test, or **h** with Šidák correction for multiple testing. **b**, **c**, **g**, **i**, **o** (Multiple) Mann-Whitney *U* test. **a**–**d**, **g**, **n**, **o** Box plots depict the median and IQR with whiskers indicating the range. **f** Data shown as mean ± S.E.M., **h**, **i** as median ± range, and **j**, **k**, **m** as parts of a whole [%]. Data in **h** and **i** from at least three independent biological replicates and **a**–**d**, **o** from at least three different mice. Relative values were determined by normalisation to the median of the respective control group (set to 1). *P ≤ 0.05, **P ≤ 0.01, ***P ≤ 0.001, ****P ≤ 0.0001. Single-cell transcriptomics data of colonic healthy human adults publicly available in the Human Gut Cell Atlas [52]. IQR interquartile range, S.E.M. standard error of the mean, WD western-style diet.

To further exacerbate the observed insulin-resistant phenotype and to assess the impact on colonic ECs, mice were subjected to a prolonged western-style diet (WD; Supplementary Table 1) for 24 weeks. The utilised model is considered a gold standard for inducing metabolic syndrome in rodents, as its combination of high-fat content and added fructose and sucrose promotes lipogenesis, elevates plasma insulin, leptin, TAG, glucose, and free FAs, and simultaneously impairs glucose tolerance [65, 66]. Colonic tissue from WD-fed mice exhibited greater proliferative activity than tissue from CD-fed mice (Supplementary Fig. 2a). Furthermore, WD-fed mice exhibited significantly reduced colonic expression of *Lgr5* and *Insr*, while expression of the qISC markers *Bmi1* and *Tert* were increased compared to CD-fed controls (Fig. 6a, b). The diminished *Insr* expression was reflected by significantly reduced colonic protein abundance in WD-fed mice (Fig. 6c, Supplementary Fig. 2b). Consistent with the 12-week HFD intervention, a colonic EC insulin resistance was established as indicated by reduced INSR downstream AKT phosphorylation (Fig. 6d, Supplementary Fig. 2c). Here, activation of colonic AMPKα remained unaffected (Fig. 6d, Supplementary Fig. 2c), suggesting preserved cellular energy availability in colonic tissue despite insulin resistance. Complementing the decreased *Lgr5* expression and reduced INSR-signalling in the colonic tissue, corresponding WD-derived colonic organoids exhibited significantly reduced growth from day 7 onward under WNT-dependent culture conditions, remaining smaller and exhibiting less pronounced crypt-like structures compared to colonic organoids derived from CD-fed controls (Fig. 6e, f, Supplementary Fig. 2d). Notably, WD-derived organoids failed to reproduce the hyperproliferative phenotype observed in vivo. This may reflect the stem cell-selective nature of the culture conditions, which favour the expansion of LGR5⁺ stem cells. As LGR5⁺ stem cell function is impaired under WD conditions, the growth of WD-derived organoids may be compromised. Further, *Insr* downregulation in WD colonic tissue was preserved in the corresponding organoids compared to CD-derived organoids (Fig. 6g). As stem cells predominantly rely on glycolysis for energy production [67] and have high proliferative capacity, insulin signalling is critical for nutrient uptake, such as glucose, FAs or amino acids. Our findings from the two independent mouse models of systemic insulin resistance, including the 12-week HFD and 24-week WD, demonstrate the development of colonic EC insulin resistance and thereby disruption of the self-renewal capacity of the LGR5^+^ ISCs. These findings are further supported by the significant reduction of the *INSR* and *LGR5* expression, in concert with strongly increased *HOPX* expression in healthy human patient-derived colonic organoids that were cultured under hyperinsulinemia mimicking conditions (Fig. 6h, i). Additionally, we treated CD- and WD-derived organoids with a mitogenic stimulus. The rationale was that, if WD-derived organoids possess an increased neoplastic potential, they would be expected to exhibit a more pronounced response towards neoplastic transformation upon exposure to the mitogenic stimulus. Here we decided to utilise the mitogen phorbol 12-myristate 13-acetate (PMA) that mimics diacylglycerol (DAG), a natural activator of Protein Kinase C (PKC) [68]. Under PMA exposure, analysis of main cancer hallmarks [69] revealed WD-derived organoids to display significantly higher cell proliferative activity compared to CD-derived organoids that underwent inhibition of cell proliferation (Supplementary Fig. 2e). While WD-derived organoids showed similar glucose uptake level compared to CD-derived organoids in the absence or presence of PMA, significantly higher L-lactate production was detected in WD-derived organoids without PMA stimulation (Supplementary Fig. 2f, g). Hence, these findings point to high anaerobic glycolytic activity, likely evoked by loss of mitochondria function in WD-derived organoids. When stimulated with PMA, L-lactate production was significantly decreased in WD-derived but not CD-derived organoids (Supplementary Fig. 2g). Interestingly, a significant increased MUC2 secretion was detected in PMA-stimulated CD- but not WD-derived organoids (Supplementary Fig. 2h). This improved differentiation under PMA stimulation of secretory ECs of CD-derived organoids, together with decreased cell proliferation, as well as unaffected glucose consumption and L-lactate production levels point towards a boost of mitochondria function by PMA stimulation in CD-derived organoids. In contrast, despite unaffected glucose consumption WD-derived organoids displayed reduced L-lactate production and did not present with increased cell differentiation as indicated by unaffected MUC2 secretion (Supplementary Fig. 2h). As differentiation of intestinal secretory ECs critically depends on mitochondrial OXPHOS activity [70], one may hypothesise that the high anaerobic glycolytic activity in WD-derived organoids switched to pentose-phosphate pathway activation enabling higher cell proliferative activity observed under PMA stimulation (Supplementary Fig. 2e). Together, the important cancer hallmarks such as loss of active stem cell function, loss of mitochondrial OXPHOS activity, low cell differentiation and sustained cell proliferation were detected in WD- but not CD-derived organoids under mitogenic environmental conditions, therefore indicating the presence of an early stage of cell transformation. To further elucidate the contribution of INSR to colonic crypt homeostasis, we interrogated single-cell transcriptomic data from healthy adults’ large intestine and rectum by using the *CELLxGENE Explorer* [52], revealing that *INSR* expressing cells (*INSR*⁺) closely overlapped with *CTNNB1*⁺ cells (Fig. 6j). Additionally, we identified two distinct populations within *CTNNB1*⁺ cells marked by highest INSR expression (Fig. 6j). Given that *CTNNB1* encodes β-catenin, a central effector of canonical WNT signalling, which governs the balance between LGR5^+^ ISC maintenance, proliferation, and differentiation [71], we hypothesised that especially cells of the lower crypt rely on INSR signalling for proliferation. Using cell annotation via* CELLxGENE Explorer*, we confirmed that cells exhibiting elevated INSR and CTNNB1 expression reside primarily within ISC and the TA zone, while low expression was found in absorptive colonocytes (Fig. 6j). Furthermore, there is evidence that INSR signalling has the ability to activate the β-catenin pathway [72]. Complementing, nearly all *INSR*⁺ colonic ECs also express *CTNNB1*, accounting for approximately one quarter of the total *CTNNB1*⁺ cell population (Fig. 6j). In the next step, we analysed which cell types within the lower crypt co-express *CTNNB1* and *INSR*, focusing on markers for EC subpopulations that were mainly affected by the presence of colonic insulin resistance such as ISC and qISC, but also DSCs, as these cells provide the niche for ISC [73]. Across all markers, approximately one quarter of the respective marker-positive cells co-express *CTNNB1* and *INSR* (Fig. 6k). To characterise molecular expressional differences underlying the two *CTNNB1*⁺*INSR*⁺^high^ clusters with the highest expression levels, differential gene expression analysis was performed using *CELLxGENE Explorer*. Amongst other markers the upper *CTNNB1*⁺*INSR*⁺^high^ cluster was strongly enriched for *RARRES2*⁺ cells. Notably, RARRES2 has been reported to be highly abundant in ISC, but is also found to a much lesser extend in secretory progenitor cells [74]. In contrast, the lower *CTNNB1*⁺*INSR*⁺^high^ cluster was characterized by enrichment of OLFM4^+^ cells, a marker associated with absorptive ECs in the colon [74]. Furthermore, established markers of active and quiescent colonic ISC populations, including *LGR5*, *ASCL2*, *CD44*, *BMI1*, *LRIG1*, *LGR4*, and *MSI1*, were predominantly localised within *CTNNB1*⁺*INSR*⁺^high^ cell populations. In addition, markers of DSCs, such as *LYZ* and *REG4*, were also predominantly enriched in the *CTNNB1*⁺*INSR*⁺^high^ populations. No specific enrichment within the *CTNNB1*⁺*INSR*⁺ cluster was observed for *HOPX*, but the number of cells positive for *CTNNB1*⁺*INSR*⁺*HOPX*⁺ was strongly limited (Fig. 6l). Although qISCs contain a similar number of *INSR*⁺ cells as active *LGR5*^+^ ISCs, they appear better equipped to withstand insulin resistance, potentially due to higher expression of other growth factor receptors (Fig. 6m). Specifically, *CTNNB1*⁺*INSR*⁺ qISCs exhibited significantly more cells positive for FA and lipid-associated receptors as well as growth factor receptors such as *ERBB* family, *IGF2R* and *IL6R* relative to ISC populations, including *APOBR*, and display a positive trend towards increased expression of *LDLR* (Fig. 6m, n, Supplementary Table 6), suggesting that these cells may strongly respond to lipid-mediated signalling, as detected in HFD-fed mice. Of note, especially *CTNNB1*⁺*INSR*⁺*HOPX*^*+*^ qISCs show highest expression of the aforementioned receptors and further *PTGER4* (Fig. 6m). Additionally, more cells positive for receptor tyrosine kinases such as growth factor receptors *ERBBR2/3*⁺, *EGFR*⁺ and *IGF2R*⁺ were detected for *CTNNB1*⁺*INSR*⁺ qISCs compared to ISCs (Fig. 6m, n, Supplementary Table 6). Particularly, *CTNNB1*⁺*INSR*⁺*RARRES*⁺ cells exhibit extremely low expression of lipid and growth factor receptors, underlining the pivotal role of INSR signalling in this ISC population. Further, *NOTCH2*, which is critically required for crypt regeneration following tissue damage [75], is also expressed in significantly more qISCs compared to actively cycling ISCs. This may contribute to an increased responsiveness of the qISC population to tissue injury and inflammatory processes. Consistent with the findings from *CELLxGENE Explorer* single-cell transcriptomic dataset, we identified elevated phosphorylation of the MAPK p44/42, a downstream effector of the receptor tyrosine kinases, in the mouse models of 12- or 24-week diet-induced insulin resistance (Fig. 6o). Notably, *CTNNB1*⁺*INSR*⁺ qISCs display higher *TNFRSF1A* and *IL6R* expression than ISCs, with *CTNNB1*⁺*INSR*⁺*HOPX*^*+*^ qISCs expressing highest *IL6R* levels. High activation of *TNFRSF1A* and *IL6R* could result downstream in elevated MAPK p44/42 activation and hence proliferation under inflammatory conditions. Supporting this, Nickel et al. demonstrated that IL-6, a key marker of chronic inflammation, was elevated in mice after 12-week HFD [29]. Consistently, colonic *Hopx* expression was significantly increased in both independent models of HFD feeding (Fig. 4c). Our findings suggest that under conditions of diet-induced excessive colonic ω6-PUFA metabolism and insulin resistance, qISCs possess a broader repertoire of growth factor receptors, which could drive unbalanced hyperproliferation of this population.

### Colonic expansion of inherently DNA error-prone qISC is associated with downregulation of MSH2

**Fig. 7:**
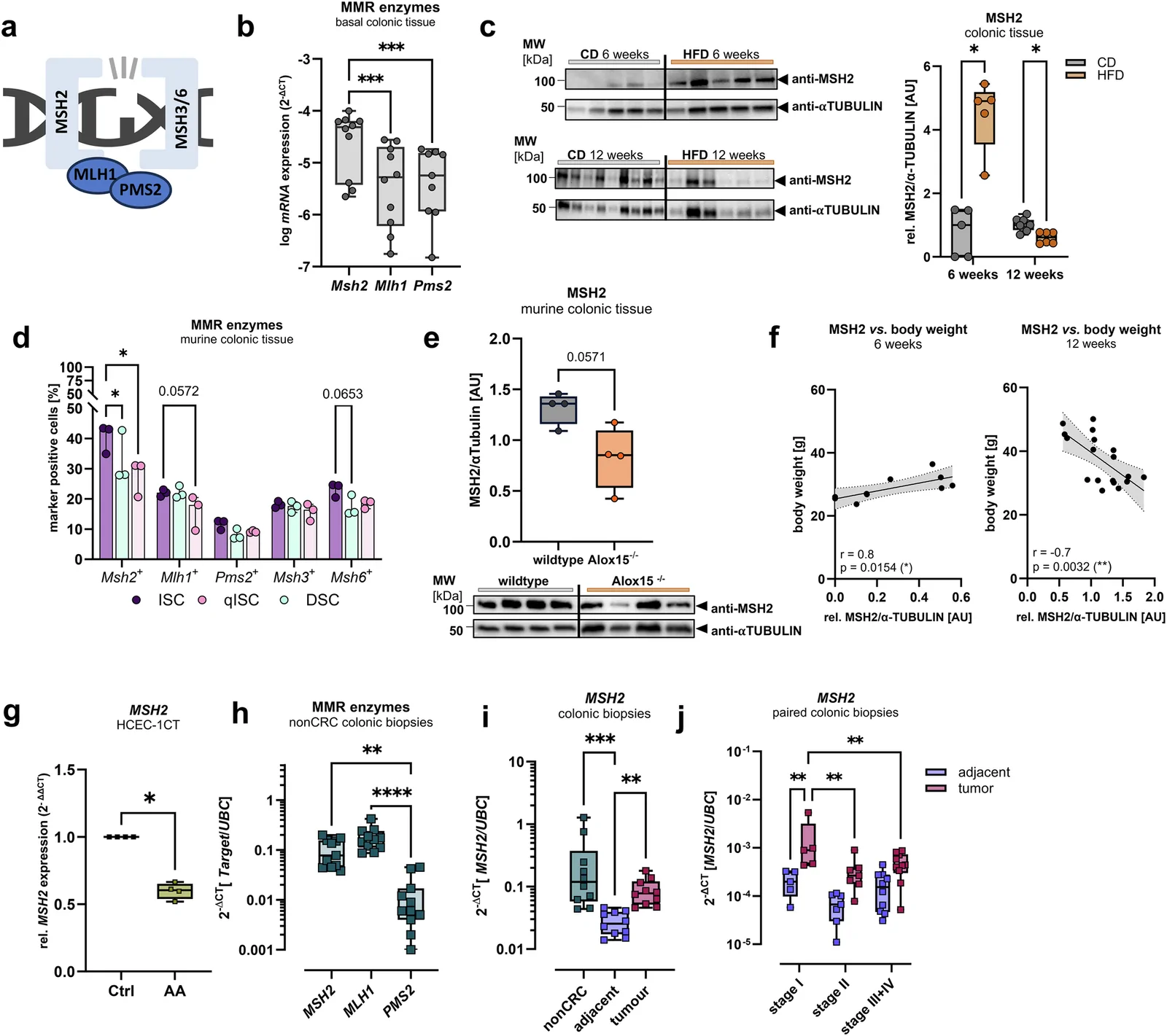
Colonic expansion of inherently DNA mismatch-prone qISC is linked to downregulation of MSH2. **a** Illustration of DNA mismatch repair (MMR) complex. **b** Log-transformed 2^-ΔCT^ values of MMR complex components in basal murine colonic tissue (*n *= 10). **c** Left: Western blot analysis using an anti-MSH2 antibody on colonic tissue from HFD- and CD-fed mice after 6 weeks (*n *= 5 per group) and 12 weeks (HFD *n *= 6, CD *n *= 9), with α-Tubulin serving as loading control. Right: Quantification of MSH2 abundance in murine colonic tissue of HFD-fed mice relative to CD-fed littermates (6 weeks: *n *= 5 per group; 12 weeks: HFD *n *= 6, CD *n *= 9). **d** Comparison of the percentage contribution of different MMR markers within ISC (*Lgr5*⁺, *Cd44*⁺, or *Ascl2*⁺), DSC (*Reg4*⁺, *Lyz*⁺, or *Egf*⁺), and qISC (*Hopx*⁺, *Bmi1*⁺, or *Msi1*⁺) populations, using single-cell transcriptomics dataset of colonic healthy mouse tissue publicly available at *CELLxGENE Explorer* dataset “Tabula Muris Senis, Large Intestine - A single-cell transcriptomic atlas characterizing ageing tissues in the mouse (10x)”. **e** Top: Western Blot of MSH2 in colonic tissue of wildtype (*n *= 4) and Alox15^-/-^ (*n *= 4) mice using anti-MSH2. Bottom: MSH2 abundance in colonic tissue of wildtype (*n *= 4) and Alox15^-/-^ (*n *= 4) mice. **f** Correlation of MSH2 level in murine colonic tissue with final body weight after 6 weeks (left; *n *= 8) and after 12 weeks (right; *n *= 18). **g** Expression of *MSH2* in human non-cancerous HCEC-1CT cells treated with 10 µM AA (*n *= 4) relative to DMSO-treated cells (*n *= 4). **h** Basal expression of MMR complex enzymes in human colonic biopsies of hospitalized normal patients (nonCRC; *n *= 11 different patients). **i** Basal expression of *MSH2* in age-matched human colonic biopsies from hospitalized normal tissue (nonCRC *n *= 10 different patients), as well as paired non-cancerous tissue adjacent to tumour side (adjacent, *n *= 10 different patients) and tumour tissue (tumour, *n *= 10 different patients) from OriGene. **j** *MSH2* expression in human colonic tumour and paired adjacent tissues (OriGene, #HCRT10303), stratified by UICC stage I (*n *= 5 different patients per group), II (*n *= 7 different patients per group), and III + IV (*n *= 10 different patients per group). **b** Two-way ANOVA with Dunnett’s test or **c**, **j** with Fisher’s least significant difference (LSD) test. **d** Two-way ANOVA using two-stage linear step-up procedure of Benjamini, Krieger and Yekutieli for controlling FDR. **e**, **g** Mann-Whitney U test. **f** Spearman correlation and linear regression with 95% confidence interval (dashed lines). **h**, **i** Kruskal-Wallis test with Dunn’s multiple comparisons test. **b**, **c**, **e**, **g**–**j** Box plots depict the median and IQR with whiskers indicating the range. **e** Data shown as median with error bars indicating the range. **i**, **j** Data shown on logarithmic y-axis. Data in **g** from independent biological replicates and **c**, **e**, **f** from at least three different mice. **c** Relative values were determined by normalisation to the median of the respective CD control group (set to 1). **h** Relative values were determined by normalisation to the paired control condition (set to 1). **d** Parts of a whole [%]. *P ≤ 0.05, **P ≤ 0.01, ***P ≤ 0.001, ****P ≤ 0.0001. AA arachidonic acid, DSC deep secretory cell, IQR interquartile range, ISC intestinal stem cells, MMR DNA mismatch repair, FDR false discovery rate, qISC quiescent intestinal stem cell, UICC Union Internationale Contre le Cancer.

Previous findings by Li et al. demonstrated interference of diet with DNA-repair mechanisms [22]. Our data revealed that HFD-associated colonic EC insulin resistance promotes inflammation-driven hyperproliferation and expansion of normally quiescent stem cells. We hypothesised that, as these cells usually reside in a dormant state, they may require lower basal DNA repair activity under homeostatic conditions. Supporting this hypothesis, Tümpel and Rudolph described quiescent stem cell populations as more error-prone and potentially more susceptible to DNA mismatch accumulation [76]. Hence, we hypothesised that increased DNA replication rates due to hyperproliferation may contribute to a higher risk of DNA damage accumulation, thereby potentially acting as an initiator of tumorigenesis. A primary system responsible for maintaining genomic integrity is the DNA mismatch repair (MMR) system. Here, MutS homolog 2 (MSH2) forms heterodimers with MSH3 or MSH6, these heterodimers associate with MutL homolog 1 (MLH1) and PMS1 protein homolog 2 (PMS2) to form a functional MMR complex (Fig. 7a). First, we investigated colonic MMR enzyme expression in CD-fed mice. *Msh2* displayed the highest expression in the colonic compartment compared to *Mlh1* and *Pms2* (Fig. 7b), underlining the pivotal role of MSH2 in the initiation of the MMR complex. Protein analysis revealed a significantly higher abundance of MSH2 in insulin sensitive colonic tissue (6 weeks of HFD), whereas a significant downregulation was observed in the colonic insulin-resistant-like phenotype (Fig. 7c). While the Western blot data reflects the bulk colonic tissue detection, we additionally utilised the *CELLxGENE Explorer* dataset “Tabula Muris Senis, Large Intestine - A single-cell transcriptomic atlas characterizing ageing tissues in the mouse (10x)” [52] to assess expression of the MMR complex at single-cell resolution. This analysis revealed that qISCs express the key MMR enzyme MSH2 significantly less frequently compared to actively cycling ISCs (Supplementary Fig. 1m, Fig. 7d), supporting published data demonstrating that this cell type has a reduced capacity to respond to DNA damage. Our findings are further supported by data from Alox15^-/-^ mice, which display a loss of active *Lgr5*^+^ ISC populations (Fig. 5f–i) and in parallel exhibit significantly reduced MSH2 abundance in colonic tissue (Fig. 7e). Further analyses identified colonic MSH2 protein level to correlate with total body weight of the mice after the 6-week intervention, while it was negatively correlated with body weight in mice with a colonic EC insulin resistance-like phenotype (Fig. 7f). To assess whether HFD-associated FAs contribute to the downregulation of MSH2, we evaluated their effects in vitro. Among the tested FAs, AA and PA significantly reduced MSH2 expression in HCEC-1CT cells compared to control (Fig. 7g, Supplementary Fig. 1n). To translate the findings from the mouse study in the context of CRC, we investigated the baseline state of the MMR complex enzymes in human colon biopsies. In healthy age-matched colonic tissue from hospitalized individuals (nonCRC), level of *MSH2* and *MLH1* were comparable, whereas *PMS2* expression was significantly lower than that of the aforementioned (Fig. 7h). Notably, *MSH2* expression was significantly lower in normal colonic tissue adjacent to the tumour site compared to paired tumour tissue from patients with CRC and nonCRC tissue, while no differences were detected between the latter two (Fig. 7i). To gain deeper insights into the basal expression of *MSH2* at different tumour stages, paired samples of adjacent normal tissue and tumour tissue were sorted by tumour stage, based on *Union internationale contre le cancer* (UICC) classification. We found that adjacent normal tissue showed significantly reduced *MSH2* exclusively in stage I, while only a non-significant trend towards decreased expression was detected between adjacent normal and tumour tissues in stages II-IV (Fig. 7j). Further, the data demonstrated a significant downregulation of *MSH2* in tumour tissue among the UICC stages II and III + IV in comparison to stage I (Fig. 7j). In summary, colonic level of MMR and especially MSH2 were downregulated in a body-weight-dependent manner in 12-week HFD-fed mice. Furthermore, *MSH2* was reduced in the colonic tissue with a higher cancer susceptibility (adjacent tissue), potentially contributing to impaired DNA repair capacity. Together, the data suggest that with advancing tumour progression, the MMR system becomes progressively suppressed, primarily through the downregulation of MSH2.

## Discussion

**Fig. 8:**
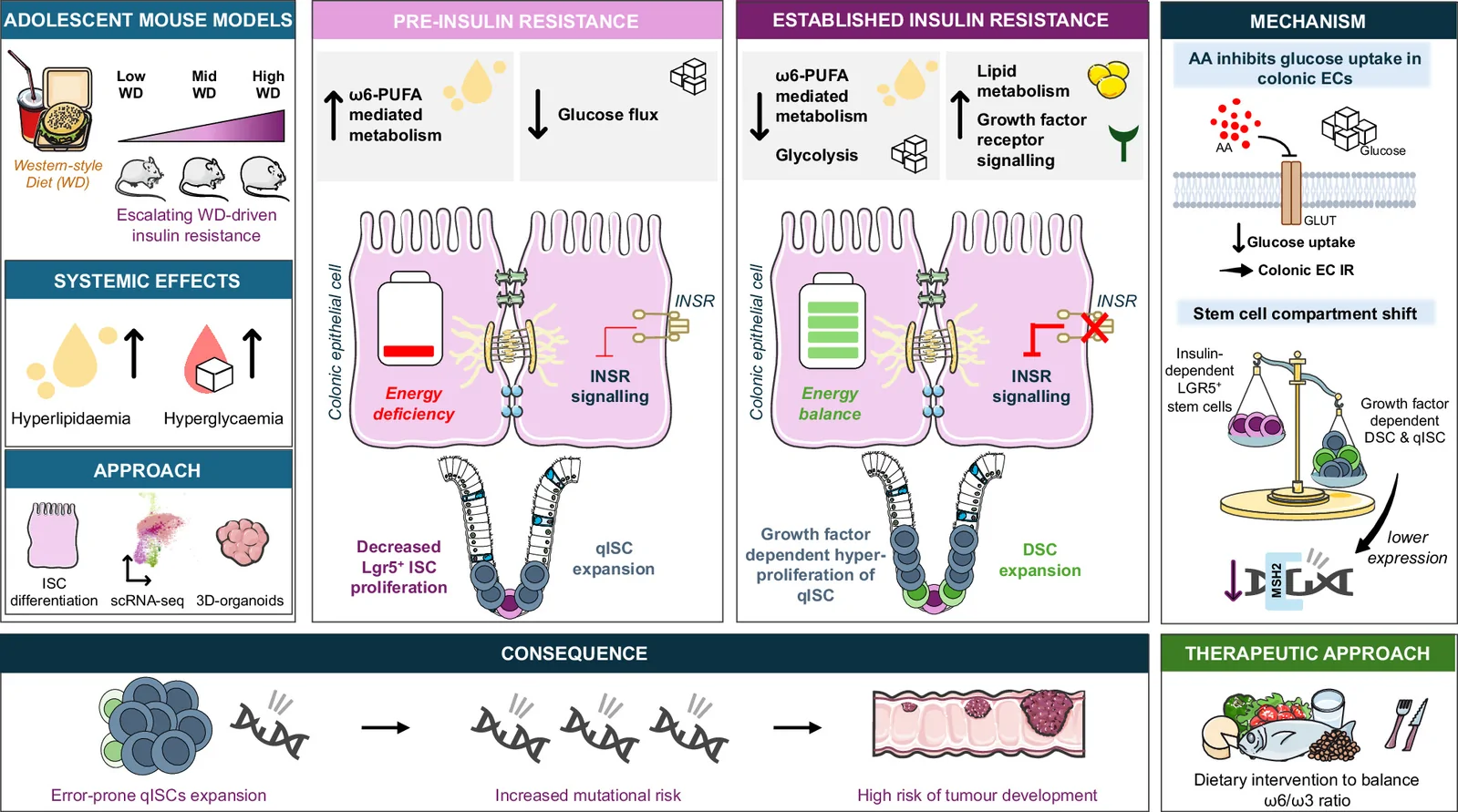
WD-induced stem cell remodelling. WD-induced hyperlipidaemia and hyperglycaemia promote ω6-PUFA metabolism and suppress glucose metabolism, resulting in epithelial energy deficiency, reduced proliferation, and qISC expansion. Established insulin resistance drives metabolic rewiring towards enhanced lipid metabolism, loss of insulin-receptor and increased growth factor signalling. Arachidonic acid-mediated inhibition of glucose uptake further favours lipid-dependent, DSCs and mismatch repair-prone qISCs at the expense of insulin-dependent LGR5^+^ stem cells, providing a potential link between WD-induced insulin resistance and eoCRC risk. DSC deep-secretory cell, IR insulin resistance, WD western-style diet, qISC quiescent intestinal stem cell, sc-RNA-seq single cell RNA sequencing.

The environmental risk factors of sporadic eoCRC remain poorly defined. Over recent decades, profound lifestyle and behavioural changes have been proposed to contribute to the decreasing age of CRC onset [77–80]. Among the various lifestyle factors implicated in eoCRC, diet is increasingly recognized as one of the most decisive contributors. In the present study, we address the impact of diet-induced colonic EC insulin resistance during early adolescence on the colonic crypt homeostasis in mice and its implications on human colonic EC homeostasis. Therefore, colonic ECs of three independent adolescent mouse models of progressive WD-driven insulin resistance were phenotyped in-depth. Ultimately, we have summarised our findings and hypothesis in Fig. 8.

We demonstrated that HFD not only induced a systemic insulin resistance, as previously reported [29, 65, 66], but may also elicited colonic EC-specific insulin resistance. Of note, this study was exclusively conducted in male mice. This choice was guided by epidemiological evidence indicating that males are more frequently and more severely affected by eoCRC than females [81, 82]. In addition, previous literature has demonstrated sex-specific differences in metabolic susceptibility, with female mice showing a relative resistance to the development of insulin resistance compared to male mice [83–85]. Further intervention studies in female mice are required to ensure the generalisability of these findings to the female sex and to confirm their translational relevance across both sexes. In 12-week HFD-fed mice we observed clear signs of impaired insulin receptor signalling in the colonic epithelium. Faecal lipidomic profiling of late adolescent mice after 6 weeks of HFD identified the HFD-associated PUFA AA, which was further found to inhibit glucose uptake in insulin sensitive colonic ECs. Further, this inhibition links to sustained upregulation of INSR, a phenomenon recapitulated in both 6-week HFD-fed mice and healthy ex vivo human colonic organoids treated with AA. Chronic INSR overexpression and hyperactivation can trigger receptor internalisation, activating negative feedback mechanisms that dampen downstream signalling. Furthermore, after 6 weeks of HFD, FAs are preferentially metabolised by ALOX and COX pathways and lipid accumulation in the form of stored visceral and subcutaneous adipose tissue, resulting in colonic EC energy deficiency and hypoproliferation. Ultimately, this culminates in an adaptive desensitization of insulin signalling, functioning as a protective mechanism against chronic overstimulation, but manifesting as insulin resistance [86]. Insulin-resistant colonic EC metabolism shifts from ALOX signalling towards energy production via β-oxidation and likely provision of FAs for membrane synthesis to support hyperproliferation. Concurrently, greater lipid uptake by tissues leads to a reduction in serum TAG levels. Notably, less visceral fat accumulates relative to subcutaneous fat, supporting the hypothesis of increased intracellular lipid consumption.

Our study demonstrates that a colonic EC-specific insulin resistance can arise alongside systemic resistance or may be driven by hyperglycaemia and hyperlipidaemia. As colonic ECs are continuously exposed to high basolateral glucose, overstimulation of the colonic EC INSR may lead to diminished insulin signalling. Notably, mice exhibiting systemic insulin resistance after 12 weeks of HFD showed unchanged colonic INSR abundance despite hyperglycaemia. Nevertheless, INSR downstream kinase AKT was significantly less activated, as well as main glycolytic enzymes, overall indicating colonic EC insulin resistance. This state was accompanied by colonic EC hyperproliferation, inflammation, and aberrant TAG metabolism. Ostermann and colleagues similarly observed reduced AKT phosphorylation in the colonic tissue of obese mice [87].

Based on the reduced growth capacity of our organoids derived from insulin-resistant WD-fed mice, colonic EC insulin resistance likely imposes a metabolic constraint on the epithelium. In particular, glucose-dependent self-renewal of active ISC was impaired, as these cells rely heavily on glycolysis and glucose uptake to support their proliferative activity [67, 88]. While we demonstrated a reduced growth capacity of organoids derived from mice fed a WD, as well as the reduction of *Insr* and *Lgr5* under hyperinsulinemia conditions, Ostermann et al. showed a decreased seeding capacity of organoids derived from insulin-resistant mice [87], and Andres and colleagues demonstrated that a specific deletion of the INSR in small intestinal epithelial tissue led to a decrease in the expression of stem cell markers [89], all highlighting the important role of the INSR in stem cell proliferation. Yet, further studies with specific INSR deletion in the colonic compartment are needed to directly link INSR with LGR5^+^ ISC proliferation. However, upon HFD-feeding of intestinal EC-specific INSR deficient mice Andres et al. showed reduced expression of qISC markers *Bmi1*, *Lrig1*, however *Hopx* was significantly upregulated upon HFD feeding [89]. It should be noted that the small intestine and the colon differ substantially in their biological function, stem-cell niche organization, and metabolic regulation [90]. The small intestine is optimised for nutrient absorption and is continuously exposed to pronounced postprandial insulin and IGF signalling, whereas the colon relies more on microbial metabolites such as short-chain FAs and displays distinct programmes of energy utilisation [91, 92]. Secondly, the small intestine and colon possess different stem-cell niche architectures. In the small intestine, Paneth cells represent a major epithelial niche component, providing WNT, EGF, and metabolic support to actively cycling Lgr5^+^ crypt base columnar (CBC) stem cells, whereas the colonic stem-cell niche depends more strongly on mesenchymal and stromal signalling cues [90]. Consequently, loss of epithelial INSR signalling may exert distinct downstream effects depending on whether ISC maintenance is primarily coupled to epithelial metabolic support, as in the small intestine, or to stromal compensatory mechanisms, as in the colon, potentially involving differential activation of PI3K-AKT-mTOR signalling, altered mitochondrial substrate utilisation, or region-specific metabolic plasticity. Finally, in addition to differences in the intestinal segment analysed, variations in experimental context may also contribute to the discrepancies between studies. The mice used by Andres et al. were generated on a mixed genetic background, incorporating both C57BL/6 and FVB strains, whereas the mice used in the present study were maintained on a pure C57BL/6 background [89]. Furthermore, Andres et al. included both male and female mice without reporting sex-stratified analyses [89]. Given that young female mice are generally less susceptible to the development of diet-induced insulin resistance than males [83–85], sex-specific differences in metabolic responses may have influenced the degree of systemic insulin resistance and consequently contributed to phenotypic differences between the models. Finally, the control diets also differed between studies with respect to their proportional fat content, providing an additional potential source of variation [89].

Across the multiple models used in this study consistent changes in REG4 and LYZ markers are detected suggesting a change in the ISC niche under metabolic stress. Although direct causal links between AA-derived PGE2 signalling and lineage-specific regulation of Reg4⁺ cells or Lyz expression have not yet been established. Lyz⁺ cells are known to emerge during epithelial injury and regenerative plasticity, representing a stress-induced secretory state. In this setting, they can act in a manner similar to qISCs, serving as facultative reserve populations that can re-enter the cell cycle and contribute to the regeneration of differentiated epithelial cell types [93]. Additionally, given that Reg4⁺ cells constitute the colonic stem-cell niche [73], it could be that ω6 lipid-driven eicosanoid signalling contributes to functional remodelling of the deep secretory niche under metabolic stress. Interestingly, DSCs are known to secrete EGF [73]. Together with the observed expression profile of EGFR in qISCs,this raises the possibility that enhanced EGF secreted by REG4^+^ cells contributes to the maintenance of the qISC state potentially as part of a feedback loop between DSCs and qISCs. However, further work is needed to substantiate these hypotheses.

Importantly, we further addressed the question of in vivo relevance of AA/ALOX signalling by analysing Alox15-deficient mice. Loss of ALOX15 led to an impairment of the active *Lgr5*⁺ stem cell compartment. In parallel, we observed a compensatory expansion of *Hopx*⁺ qISCs and *Reg4*⁺ niche-forming secretory cells. Notably, these changes occurred under basal in vivo conditions, supporting a functional role for ALOX15-dependent signalling for ISC regulation. Insulin signalling does not appear to be altered in Alox15^-/-^ mice. Further nutritional-intervention studies with these mice will be needed to investigate the role of ALOX15 in insulin resistance development.

A recent study demonstrated that loss of *Insr* expression potentiated intestinal inflammation and impaired mucosal healing following AOM/DSS challenge in mice [87]. We demonstrated that AA-driven metabolic impairment of ISCs may compromise colonic homeostasis, the maintained regenerative capacity suggests compensatory activation of an alternative stem cell-like population for mucosal repair. Previously it has been described that, qISC can smoothly transition to maintaining mucosal homeostasis when the active ISCs are ablated or threatened by damage due to radiation or environmental factors [94]. A similar effect was found by Wang et al., who were able to identify an increase in qISC and enhanced proliferation rates by stimulating mouse small intestinal organoids with AA [53]. Our data indicate that qISCs express higher levels of INSR-independent growth factor receptors (especially *EGFR* and *ERBB2/3)*, might display higher responsiveness to inflammation-driven IL-6 signalling due to high *IL6R* expression, and likely possess a greater repertoire of ω6-PUFA-dependent and other lipid receptors. This combination renders qISCs not only more insulin-independent than active ISCs but also more prone to proliferation under pro-inflammatory conditions associated with high lipid accumulation or colonic EC insulin resistance. Additionally, the murine small-intestinal organoids used in the study by Wang et al. displayed morphological alterations [53], which appeared similar to those observed in our work. The spheroid-like phenotype may be a result of the presence of PGE_2_, which accumulates in the medium of AA-stimulated organoids as a product of AA metabolism and might also be a potent driver of cellular compositional changes, whereas alterations in adhesion capacity or epithelial polarity did not appear to contribute substantially to the phenotype observed in AA-treated organoids. We observed increased *EPCAM*, *OCLN*, and *ITGB4* expression, while key polarity and junctional markers (*CDH1*, *EZR*, *TJP1*, *CLDN1*) remained unchanged, suggesting subtle reinforcement or remodelling of epithelial adhesion and organization without evidence of a global loss of apical-basal polarity. Increased *EPCAM* expression is commonly associated with enhanced epithelial identity and cell-cell adhesion, whereas elevated *OCLN* levels may indicate adaptive modulation of tight junction composition and epithelial barrier maintenance. Similarly, increased *ITGB4* expression could point towards altered epithelial-matrix interactions and enhanced basolateral anchorage to the extracellular matrix. Taken together, these findings support the notion that the observed phenotype is characterised by subtle epithelial remodelling and junctional adaptation rather than a broad polarity defect or epithelial-to-mesenchymal transition-like state. Furthermore, it has been shown that PGE_2_ induces proliferation and may also contribute to ion channel activation [53, 95, 96]. The study by Wang et al. further demonstrated that the observed phenotype appears to be reversible. Moreover, re-exposure of spheroids to normal growth medium without AA resulted in a significantly increased number of organoid buds, suggesting that AA treatment induces a reversible proliferative cellular state [53]. It is important to emphasize that the experiments in this study were conducted under conditions of excess AA to colonic ECs. The lipidomic analysis does not permit determination of absolute concentrations, which precludes accurate estimation of the physiological concentrations present in the intestinal lumen of mice used in this study. However, a study by Huss et al. reported a median AA concentration of 0.33 µmol/g dry faeces in human control subjects, whose nutritional status was not defined, indicating that substantial pools of arachidonic acid are present within the colonic lumen [97]. Based on this it cannot be stated with certainty whether a concentration of 10 µM reflects physiological relevance, and this would require further epidemiological investigation, ideally incorporating detailed information on participants’ nutritional status and related metabolic parameters.

Under balanced conditions, downstream metabolites of AA have been implicated in tissue repair and the resolution of inflammation [16]. This suggests that AA signalling follows a beneficial balance under normal healthy physiological conditions, whereas disruption of this equilibrium by excessive dietary AA intake may compromise intestinal homeostasis.

While we observe a consistent HFD- and AA-associated activation of inflammatory signalling pathways, including CASP1, IL6R signaling, MAPK pathway activation, and increased PGE2 production, our data do not allow us to determine the directionality of these events relative to qISC expansion and colonic insulin resistance. Specifically, it remains unclear whether inflammatory signalling acts as an upstream driver of these phenotypes or whether it represents a downstream or parallel consequence of altered lipid metabolism and epithelial reprogramming. We therefore acknowledge a limitation of the present study in that it does not include functional intervention experiments targeting inflammatory pathways. As such, we cannot exclude the possibility that the observed inflammatory responses are secondary to AA-driven metabolic changes rather than causally required for qISC expansion or insulin resistance. Future studies employing pharmacological or genetic inhibition of key inflammatory mediators will be required to resolve this causal hierarchy and to determine whether this can reverse the observed epithelial and metabolic phenotypes.

The qISC population plays a protective role by safeguarding homeostatic ISCs and maintaining tissue integrity under AA-driven inflammation. However, long-term activation of qISCs may increase mutation burden, as these cells exhibit inherently higher error rates, because of reduced efficiency in DNA MMR [21]. Consistently, we observed a reduction in MSH2, a key MMR enzyme, in the 12-week HFD-mice, despite hyperproliferation. Supporting this, Li et al. demonstrated that under a NWD1 diet, increased contribution of the BMI1⁺ qISCs to mucosal maintenance is associated with a higher mutation load [22]. Thus, both the elevated mutation burden and the stem/progenitor potential of BMI1⁺ cells may enhance the tumorigenic potential [27, 98]. While Li and colleagues attributed these effects primarily to dietary vitamin D deficiency, our data suggests that excessive levels of the WD-associated AA can similarly promote qISC activation and might also be associated with a higher mutagenic risk. However, further studies employing orthotopic transplantation approaches or AOM/DSS-induced CRC models in vivo in combination with dietary interventions will be required to directly assess the increased neoplastic potential associated with qISC expansion.

Importantly, several negative findings further refine the mechanistic interpretation of our data. While the data obtained from 2D HCEC-1CT cell line experiments suggest that effects on INSR are more likely to emerge following prolonged stimulation, it should be noted that the supernatant was not analysed in these experiments, nor were additional assessments of downstream insulin receptor signalling performed. Consequently, it is difficult to determine whether downstream INSR signalling is already impaired by AA within this relatively short experimental timeframe. It is not surprising that PA, OA, and SA did not activate *COX* and *LOX*, as these enzymes exhibit strong substrate specificity for AA and LA [99, 100]. These findings further support the hypothesis that lipid-mediated inflammatory signalling is not driven by individual FAs in a non-specific manner but may instead depend on AA as a selective mediator or on synergistic effects of FA mixtures. However, the latter case was not addressed in this study and would require further validation using defined FA combinations. Furthermore, as limitation it should be noted that the proposed high-turnover lipid cycle remains a working hypothesis, as no isotope-tracing experiments or direct measurements of FA uptake and oxidation were performed in the present study. Future metabolic flux analyses will therefore be required to experimentally validate this model. In conclusion, exposure to a WD primes colonic EC, promoting metabolic colonic EC insulin resistance and therefore may increase susceptibility to neoplastic transformation of colonic ECs. Our findings indicate that this effect is associated with activation of the inherently DNA mismatch-prone qISC population, which compensates to maintain intestinal homeostasis under metabolic stress [22, 27]. However, our study is limited, as it includes a relatively small sample size, particularly within the human nonCRC cohort. Future studies involving larger and more comprehensively characterised patient cohorts, including detailed information on body mass index, dietary habits, metabolic status, and other relevant clinical parameters, will be required to validate and extend the observations reported here.

While the present study cannot conclusively determine whether the proposed mechanism is specific to eoCRC, previous studies have demonstrated that ageing is associated with profound changes in ISC metabolism, including reduced stem cell number and function, as well as alterations in FA-dependent signalling pathways [101]. Based on these observations, we hypothesise that stem cells in aged organisms may exhibit a diminished responsiveness to high lipid loads, potentially resulting in a distinct mode of diet-stem cell interaction that could influence CRC development in late-onset CRC. However, this hypothesis requires further investigation involving the comparison of adolescent and adult mice under dietary intervention.

Finally, epidemiological studies indicate that diabetogenic processes characterised by insulin resistance and associated hyperinsulinemia are linked to increased odds of CRC, including early-onset disease [102]. In addition, independent data suggest that elevated insulin levels may contribute to the initiation of colorectal tumorigenesis [103]. Evidence from experimental mouse models further indicates that insulin resistance is closely associated with dysregulation of AA metabolism. Specifically, increased AA production has been shown to promote hyperinsulinemia and insulin resistance, whereas pharmacological or genetic inhibition of AA biosynthesis improves insulin sensitivity. Moreover, AA-derived eicosanoid signalling pathways are consistently altered in insulin-resistant states and contribute to impaired insulin signalling and chronic low-grade inflammation [104, 105]. Together, these findings suggest that attenuation of the AA pathway may improve systemic insulin sensitivity, which, in line with our data, could also enhance epithelial insulin responsiveness. Considering that younger adults often exhibit poorer ω3-FA status compared with older populations [106, 107], this may contribute to an imbalance in the ω6/ω3 fatty acid ratio and thereby potentiate AA-derived inflammatory signalling, including COX/ALOX-mediated pathways. Accordingly, personalized ω3 supplementation may represent a plausible preventive strategy to restore intestinal metabolic homeostasis and reduce pro-tumorigenic inflammatory signalling. Still translation to human disease remains limited by interspecies differences and the multifactorial aetiology of eoCRC. Therefore, while our findings provide mechanistic insight into diet-induced epithelial reprogramming, prospective human studies in young adults are required to determine clinical relevance and to evaluate whether colonic EC insulin resistance, an imbalanced ω6/ω3 ratio, and altered qISC activation states may serve as early biomarkers of CRC risk.

## Methods

### Patient recruitment and sample collection

RNA from CRC patients utilised for quantitative real time PCR (RT-qPCR) was purchased from OriGene (OriGene Technologies, Inc., Rockville, MD, USA).

Colonic biopsies from healthy individuals were obtained during endoscopy as part of regular patient management in the Medical Department 1, University Hospital Schleswig-Holstein Campus Luebeck, Germany. Biopsy specimens were obtained from descending colon, sigmoid colon, and rectum. Serum samples of healthy patients (nonCRC) were collected during routine patient management in the Medical Department 1, University Hospital Schleswig-Holstein Campus Luebeck, Germany. Serum samples of patients with CRC were collected prior to administering anaesthesia during surgery. All human serum samples collected in the context of this study were obtained from participants and patients who had fasted for at least 10 hours prior to blood collection. Patients’ characteristics are depicted in Supplementary Tables 4 and 5. Investigators were not blinded for the above-mentioned studies.

### Animal experiments

All mice were housed under specific pathogen-free conditions on a regular 12-hour light-dark cycle with *ad libitum* access to food (specified in Supplementary Table 1) and water.

For the 6-week HFD intervention, male C57BL/6 J mice (Jackson Laboratories, ME, USA) were habituated to laboratory conditions for at least 10 days prior to the start of the experiment. Mice were randomly assigned either a control diet (CD, Autoclavable rodent diet 5010, LabDiet, St. Louis, USA) or the HFD D12492i (Research Diets Inc., New Brunswick, USA). All mice were sacrificed at the end of the 6th week for serum and organ collection.

For the 12-week HFD feeding model, male C57BL/6 N mice (Charles River Laboratories, Sulzfeld, Germany) were used, receiving either a regular chow diet (CD, EF acc. D12450B mod., ssniff®, Soest, Germany) with vehicle treatment or HFD (EF acc. D12492 [I] mod., ssniff®, Soest, Germany) with vehicle treatment, as previously described [29].

Six-months-old male B6·129S2-Alox15tm1Fun/J (Alox15^-/-^) mice and C57BL/6 J wild-type mice were kindly provided by Prof. Dr. Christian Sadik. Briefly, mice were purchased from The Jackson Laboratory (Bar Harbor, ME, USA) and bred and housed in a 12-h light-dark cycle at the animal facility of the University of Luebeck (Luebeck, Germany). Alox15^-/-^ mice were genotyped according to instructions of the Jackson Laboratory with slight modifications, as described previously [108]. Organ harvest of these animals was approved by the Schleswig-Holstein state government.

For murine colonic organoid generation, male mice were ordered from University Medical Center of Johannes Gutenberg University Mainz (Mainz, Germany). The Dnmt1 2lox mice were made as a congenic strain by backcrossing to C57BL/6 for 10 generations and then intercrossing to homozygous the 2lox allele (JAX Lab, Dnmt1 flx/flx). Embryos of these mice were transferred and bred in the animal facility of the University of Luebeck. Male Dnmt1 flx/flx mice, corresponding to wildtype C57BL/6 were randomly assigned into two groups placed on the Control (to Surwit) diet (ssniff®, Soest, Germany) starting at 4 to 6 weeks of age. After 2 weeks on the Control (to Surwit) diet, animals were randomly assigned to two groups: one group remained on the Control (to Surwit) diet, while the other was switched to the Surwit diet supplemented with sucrose (EF acc. D12331, ssniff®, Soest, Germany) and supplemented with 45% (w/v) glucose and 55% (w/v) fructose via the drinking water. Mice were maintained on the respective diet for 24 weeks (median age 222 days, IQR 215-226.5 days) before organ sampling.

For all animal experiments no randomisation was used and no blinding was done. Additionally, all sample size calculations were performed according to standard procedures.

### Generation of murine and human colonic 3D-organoids

Human and murine colonic crypts were isolated using protocols from StemCell™ Technologies. Briefly, colon from WD- and CD-fed mice were washed in cold DPBS (Gibco, Waltham, MA, USA, #14190144) and incubated with Gentle Cell Dissociation Reagent (GCDR; StemCell™ Technologies, Vancouver, Canada, #100-0485) for 45 min at room temperature. Supernatants were passed through 70 µm filters (StemCell™ Technologies), and fractions enriched in crypts were collected, centrifuged, and resuspended in cold DMEM/F12 (Gibco, #11530566). After a second centrifugation, crypts were embedded in UltiMatrix Reduced Growth Factor Basement Membrane Extract (UltiMatrix; R&D Systems, Minneapolis, USA, #BME001) and seeded in 50 µL domes in pre-warmed 24-well plates (Sarstedt, Nürnbrecht, Germany, #83.3922.300). Domes were solidified at 37°C for 30 minutes, overlaid with IntestiCult™ Organoid Growth Medium (StemCell™ Technologies, #06010) supplemented with 100 ng/µl IGF1 (PeproTech Gmbh, Hamburg, Germany, #250-90), and 50 ng/µl FGF-basic (PeproTech Gmbh, Hamburg, Germany, #450-33), and maintained at 37°C, 5% CO₂, with medium changes every 2-3 days.

Human colonic crypts were obtained from biopsy specimens of the descending colon, sigmoid colon, and rectum. Samples were washed, minced, and incubated in GCDR (StemCell™ Technologies). After centrifugation, fragments were resuspended in cold DMEM/F12 (Gibco) supplemented with 1% (w/v) BSA (AppliChem/Illinois Tool Works Inc., Chicago, USA, #A1391) and 15 mM HEPES (Gibco, #15630-056). Supernatants were filtered, crypts counted, quality assessed and then pelleted. Isolated crypts were mixed 1:1 (v/v) with Human IntestiCult™ Serum-free Organoid Growth Medium (SFhOGM; StemCell™ Technologies, #100-0340) and Cultrex BME Type 2 (Cultrex; R&D Systems, #3533). Approximately 1000 crypts per 50 μL dome were seeded to a 24-well plate (Sarstedt) and maintained at 37°C, 5% CO₂, with medium changes every 2-3 days.

### Generation of murine primary colonic epithelial cells (pCEC)

The colon of sacrificed mice was excised, opened longitudinally, washed, and cut into small pieces. Tissue fragments were shaken for 10 minutes in 1 mM dithiothreitol (DTT, Carl Roth, Karlsruhe, Germany, #6908) in DPBS (Gibco) at room temperature. After removing the supernatant, the pieces were incubated for 2 h in 1 U/ml Dispase (StemCell™ Technologies) in DPBS (Gibco) containing 1% (w/v) BSA (AppliChem/Illinois Tool Works Inc.) at 37°C with agitation. The resulting suspension was passed through a 70 µm cell strainer (Sarstedt), washed with PBS, isolated cells were subsequently counted and further processed.

### Organoid culture

Primary organoid cultures were passaged after 7 to 14 days. Medium was removed, and cold GCDR was added to the dome. After one minute incubation with GCDR at room temperature, the dome and organoids were disrupted by pipetting the suspension. The well was washed again with GCDR. After incubating the organoids on ice for 10 min, followed by centrifugation, organoids were washed in DMEM/F12 with 15 mM HEPES containing 1% (w/v) BSA, centrifuged and resuspended in a 1:1 (v/v) mixture of SFhOGM (StemCell™ Technologies) and Cultrex (R&D Systems) or pure UltiMatrix (R&D Systems), respectively. Next, 50 µl domes were formed on a 24-well plate (Sarstedt) and placed at 37 °C for solidification. The domes containing human colonic organoids were overlaid with 500 µl SFhOGM and domes containing murine colonic organoids were overlaid with 500 µl IntestiCult™ Organoid Growth Medium supplemented with 100 ng/µl IGF1 (Pepro Tech Gmbh) and 50 ng/µl FGF-basic (Pepro Tech Gmbh). Complete medium changes were performed every 2-3 days. Organoids were regularly tested for mycoplasma contamination.

### Real-time proliferation analysis of 3D-organoid culture

Real-time proliferation data were obtained using the IncuCyte SX5 (Sartorius, Göttingen, Germany). Organoids were seeded into a 48-well plate (Corning, Glandale, USA, #CLS3548) according to the protocols provided by Sartorius. For stimulation 10 µM arachidonic acid (AA; Sigma Aldrich, MO, USA #10931), dissolved in DMSO, or a respective DMSO control (containing ≤ 0.1% DMSO, Sigma Aldrich, #D2438) were freshly added to the medium. Every 48 hours, organoid culture medium was renewed containing respective stimuli. Continuous growth of organoids was quantified every 6 h for a total of 10 days using the IncuCyte Imaging System (Essen BioScience, Ann Arbor, USA).

To mimic hyperinsulinemia conditions, organoids were stimulated with 1 µM insulin (Sigma Aldrich, #I0516). Subsequently, organoids were cultured for 72 h, and continuous growth was quantified as described above. To evaluate neoplastic potential, CD- and WD-derived organoids were seeded and allowed to recover for 24 h prior to stimulation. The culture medium was then replaced with fresh medium containing 1 µM phorbol 12-myristate 13-acetate (PMA; InvivoGen, #tlrl-pma, France) or a respective control condition ( ≤ 0.1% DMSO v/v). Organoid growth was monitored over the subsequent 72 hours and quantified as described above. The average Organoid Object Total Brightfield Area (*10^4^) in µm^2^ was normalized to 0 days 0 hours 0 minutes. Analysis was conducted using IncuCyte Organoid QC analysis software (v2022B Rev2, Sartorius).

### Microarray analysis

Gene expression profiling was performed using Affymetrix microarrays (now part of Thermo Fisher Scientific under the Applied Biosystems brand).

Raw intensity data (.CEL files) were imported and preprocessed using the oligo package (v1.76.0) in R (v4.6.0). Background correction, quantile normalisation, and summarization were performed using the Robust Multi-array Average (RMA) algorithm. Low-variance and non-informative probesets were removed prior to differential expression analysis using the nsFilter function from the genefilter package (v1.94.0).

Differential expression between Alox15^-/-^ mice (*n* = 6) and C57BL/6 wildtype controls (*n* = 4) was assessed using the limma package (v3.68.1). A linear model was fitted to the normalized expression matrix using a no-intercept design matrix, and the contrast of interest (Alox15^-/-^ − wildtype) was extracted using makeContrasts. Empirical Bayes moderation of standard errors was applied via eBayes to improve statistical power for small sample sizes. Differentially expressed genes were identified using a fold-change threshold of |log₂FC | ≥ 0.3 and ≤ -0.2, with multiple testing correction applied using the Benjamini–Hochberg false discovery rate (FDR) method. The full ranked gene list was annotated by merging probeset identifiers with a custom annotation file containing gene symbols and Entrez IDs.

### Cell culture

Immortalized human colonic epithelial cell line HCEC-1CT was kindly provided by Damian Graczyk (Institute of Biochemistry and Biophysics Polish Academy of Sciences, Warsaw, Poland) with permission from Jerry W. Shay (UT Southwestern Medical Center, TX, USA). The cells were grown in DMEM/F12 (1:1) (Gibco) containing 2% (v/v) heat-inactivated fetal calf serum (FCS), 100 U/ml penicillin, and 100 mg/ml streptomycin. With each passage the medium was freshly supplemented with 1 µg/ml hydrocortisone (Sigma Aldrich, #I0516), 5 nM sodium selenite (ThermoFisher Scientific, Waltham, USA, #11366449), 20 ng/ml recombinant human epidermal growth factor (EGF; Roche, Basel, Switzerland, #11376454001), 10 µg/ml insulin (Sigma Aldrich), and 2 µg/ml human holo-transferrin (Biozol, Echingen, Germany, #ABM-G599). For fatty acid (FA) stimulation, 50,000 cells/well were seeded into a 24-well plate. After 24 h, 10 µM AA (Sigma-Aldrich) or palmitic acid (PA; Sigma-Aldrich, #P0500) dissolved in DMSO, linoleic acid (LA; Sigma-Aldrich, # L5900) or oleic acid (OA; Sigma-Aldrich, #O1257) dissolved in DEPC-treated water (Gibco), or 10 µM stearic acid (SA; Sigma-Aldrich, #800673) dissolved in absolute ethanol (EtOH; Carl Roth, 5054) were added to each well. Medium and FA stimulation were refreshed after 96 h, and cells were harvested after 144 h (6 days) for counting using the QuadCount™ (Accuris Instruments, Sayreville, USA) and subsequent analyses. Cells were regularly tested for mycoplasma contamination.

### RNA extraction, cDNA synthesis and quantitative real-time PCR

Isolation of total RNA from HCEC-1CT cells, human colonic biopsies or murine colonic tissues was performed using the innuPREP RNA Mini Kit (Analytic Jena AG, Jena, Germany, #845-KS-2040010) according to the manufacturer’s instructions. Isolation of total RNA from organoids was performed using the Norgen Single Cell Isolation Kit (Norgen Biotek Corp., Thorold, Canada, #SKU51800) according to the manufacturer’s instructions. After binding of RNA to the RNA column, additional DNA digestion was performed with 4 units of DNase I (Sigma Aldrich, #AMPD1-1KT). RNA quality was assessed using the Agilent 2100 Bioanalyzer (Agilent Technologies, CA, USA), and samples with a RIN ≤ 3.0 were excluded.

For cDNA synthesis, a maximum of 1 µg RNA was transcribed utilizing Oligo(dT)18 primers and the RevertAid H Minus reverse transcriptase (Thermo Fisher Scientific #EP0451).

Target amplification was performed by RT-qPCR on the StepOnePlus^TM^ or the QuantStudio^TM^ 1 system (Thermo Fisher Scientific), in combination with the PerfeCTaTM SYBR® Green SuperMix (Quantabio, MA, USA, #95055-02 K) and target-specific oligonucleotides (listed in Supplementary Table 7). Data were analysed using the 2^-ΔCT^ or 2^-ΔΔCT^ algorithm (for murine colonic samples to the median CD target expression for each of the respective animal experiments, due to baseline variations) by normalisation to *Actb, ACTB*, *Ubc*, *UBC*, or *Rn18S*.

### Bulk RNA sequencing of patient-derived colonic organoids

Stimulated and control-treated organoids were harvested and total RNA was isolated following the manufacturer’s instructions of the Norgen Single Cell Isolation Kit (Norgen Biotek Corp.), including on-column DNA digestion using DNase I. RNA was quantified using the NanoDropTM OneC spectrophotometer (Thermo Fisher Scientific) and verified to have an RNA integrity number (RIN) > 5 using the RNA 6000 Nano Kit and the 2100 Bioanalyzer instrument (Agilent Technologies, #5067-1511).

Bulk mRNA sequencing was performed at Novogene. Following poly(A) enrichment and non-directional mRNA library preparation, paired-end 150 bp sequencing was carried out on the NovaSeq X Plus platform (Illumina) to a depth of approximately 30 million reads per sample. Raw sequencing reads were processed using the nf-core/RNAseq workflow (v3.17.0) implemented in Nextflow (v24.04.4). Adapter trimming and quality filtering were performed using fastp, and alignment was conducted against the human reference genome GRCh38 using STAR (v2.6.1 d) for mapping and Salmon (v1.10.3) for quantification, with sequence-specific and GC bias correction enabled (seqBias, gcBias) and 100 bootstrap replicates per sample. Salmon quantifications were imported at the transcript level with tximport (v1.40.0) and summarized to gene-level TPM abundances via summarizeToGene.

### Protein isolation from formalin-fixed paraffin-embedded (FFPE) tissue

To isolate proteins from FFPE colon tissue, the Qproteom FFPE Kit from Qiagen (QIAGEN N.V., Venlo, Netherlands, #37502) was used according to manufacturer’s instructions.

Protein isolation from colon tissue, native frozen biopsies were homogenized in denaturing lysis buffer containing 1% (w/v) sodium dodecyl sulfate (SDS), 10 mM Tris (pH7.4), 2% (v/v) phosphatase inhibitor 2 and 3 (Sigma-Aldrich #P5726 and #P0044), and 1% (v/v) protease inhibitor (Sigma-Aldrich #P8340). The homogenate was heated at 100 °C for 5 min, treated with ultrasonication, and cleared by centrifugation at 12,000 × g and 4 °C for 15 min. Whole protein concentration was determined using the Roti® Quant universal assay (Carl Roth, #0120).

### Sodium dodecyl sulfate-polyacrylamide gel electrophoresis (SDS-PAGE) and immunoblotting

For SDS-PAGE, whole protein extracts were separated on a CriterionTM TGXTM 4-15% polyacrylamide gel (Bio-Rad, CA, USA, #5671085). Separated proteins were blotted to a PVDF membrane by semi-dry transfer. After blocking in 5% (w/v) non-fat milk in Tween20-Tris-buffered saline (T-TBS), membranes were probed with specific primary antibodies (Supplementary Table 8), followed by respective horseradish peroxidase (HRP)-conjugated secondary antibodies. Specific proteins were detected using chemiluminescent HRP substrate on the ChemiDocTM XRS+ (Bio-Rad) or the iBright 1500 imaging system (ThermoFisher) and quantified in ImageJ. To ensure similar transfer and equal loading of proteins, the abundance of target proteins was normalized to α-Tubulin.

### Dot blot

Protein samples were directly applied onto nitrocellulose membranes (Amersham™ Protran® Western-Blotting-Membrane, GE Healthcare Life science, Il, USA) and air-dried for 30 min at room temperature. Membrane was blocked in 5% (w/v) non-fat milk (AppliChem) in T-TBS for 1 h, followed by incubation with an anti-MUC2 antibody (Supplementary Table 8) over night at 4 °C. After two washes in T-TBS, membrane was incubated with Anti-rabbit IgG HRP (Supplementary Table 8) for 1 h at room temperature and washed again. Signals were detected using SuperSignal™ West Atto Ultimate Sensitivity Chemiluminescent Substrate (ThermoScientific, #A38554) and visualized on the iBright™ CL1500 Imaging System (ThermoFisher). To determine equal transfer and loading, membranes were stripped and stained afterwards with Swift Membrane Stain™ (G-Biosciences, MO, USA, #786-677) to normalise the abundance of MUC2.

### Histology and microscopy analyses

Immunohistochemical (IHC) staining in Formaldehyde (Th. Geyer BIOSOLUTE, Renningen, Germany) (FFPE)-fixed and paraffin-embedded murine colonic tissue biopsies was performed according to standard protocols. After deparaffinization, rehydration, endogenous peroxidase blockage, a blockage in 2% (w/v) BSA, and antigen retrieval, tissue slides were probed with specific primary antibodies or isotype control antibodies, followed by respective HRP-conjugated secondary antibodies or HRP-labelled polymers (listed in Supplementary Table 8). Tissue slides were incubated with DAB-substrate (Dako, Jena, Germany, #GV825) and counterstained with Haemalaune. Images were obtained and analysed on an Axio Scope.A1 microscope (Zeiss, Oberkochen, Germany) utilizing the ZEN imaging software (v2.3, Zeiss). If appropriate, stained areas were quantified using the colour deconvolution plugin for the software ImageJ software (version 1.54i) [109]. Crypt length was quantified using Zen imaging software (v2.3, Zeiss). Six complete colonic crypts sectioned longitudinally from base to apex were measured per mouse within one image, and the median value was calculated for analysis.

### Lipidomic analysis of murine faecal samples

A Dionex Ultimate 3000 RS LC-system coupled to an Orbitrap mass spectrometer (QExactive, ThermoFisher Scientific, Bremen, Germany) equipped with a heated-electrospray ionization (HESI-II) probe was used. All solvents were of LC-MS grade quality and were purchased from Merck (Darmstadt, Germany). Collected faeces samples were homogenized in 150-500 µl water using a tissue homogenizer (Bullet Blender, Next Advance, NY, USA). After centrifugation (2000 *x* g/ 4 °C/ 5 min), supernatants were diluted accordingly to reach a protein concentration between 0.5-1.2 mg/ml. Exact protein concentration was determined using a Bradford assay (Sigma-Aldrich, #B6916) according to the manufacturer’s instruction. Lipid profiling was performed as described previously [110]. Briefly, lipids were extracted by adding 1 ml of methanol/methyl tertiary-butyl ether/chloroform (1.33:1:1, v/v), containing butylated hydroxytoluene (100 mg/l, Sigma-Aldrich, #B1378) and 2.5 ml/l of SPLASH internal standard mix (Avanti Polar Lipids, Alabaster, AL, USA, #330707), to 50 µl of tissue homogenate. After incubation and centrifugation, supernatants were dried under vacuum and resuspended in 50 µl of methanol/isopropyl alcohol (1:1, v/v) for LC-MS/MS analysis. Extracted lipids were separated on an Accucore C30 RP column (150 × 2.1 mm, 2.6 µm) using acetonitrile/water 6:4 v/v as eluent A and isopropyl alcohol/acetonitrile (9:1, v/v) as eluent B; 10 mM ammonium acetate (Sigma-Aldrich, #A7330) and 0.1% (v/v) formic acid (Biosolve, Dieuze, France, #069106) was added to both eluents. Ionisation and data acquisition with data-dependent MS² scans (top 15) was performed as described previously [110, 111]. Lipid species were identified based on an in-house database, according to exact mass, retention time, fragmentation ions, and isotopic pattern. The area under the peak was normalized to the internal standard and the protein concentration. Pooled samples at 5 concentrations were used as quality controls. In accordance with the 3 R principle we analysed samples that were derived from a previously conducted experiments, therefore, stool samples from the 12-week mouse model were not available for analysis.

### NMR metabolome analysis

Thawed aliquots of frozen serum samples were mixed 1:1 (v/v) with 75 mM sodium phosphate buffer (pH 7.4). 600 µL of the mixed samples were transferred into a 3 mm Nuclear Magnetic Resonance (NMR) tube. NMR analysis was conducted on a Bruker 600 MHz Avance III HD spectrometer with a TXI probe, using a Bruker SampleJetTM automatic sample changer with cooling set to 6 °C. The experiments were performed following the Bruker in vitro Diagnostic Research (IVDr) protocol. To ensure quality control, the NMR spectrometer was calibrated daily using a strict standard operation procedure protocol based on the method described by Dona et al. [112]. For each sample, four different 1H NMR experiments were measured at 310 K. One-dimensional (1D) proton spectra were recorded using a standard Bruker pulse program (noesygppr1d) with pre-saturation water suppression during the 4-second recycle delay. Additionally, a 1D Carr-Purcell-Meiboom-Gill (CPMG) spin-echo experiment (pulseprogram: cpmgpr1d) was acquired to suppress proteins and other macromolecular signals. These spectra were obtained with 32 scans and 131,072 data points. Moreover, 2D J-resolved and diffusion-edited experiments were performed. From the acquired spectra, concentrations of 39 metabolites and 112 lipoproteins were automatically determined using B.I. Quant-PS 2.0.0 and the Bruker IVDr Lipoprotein Subclass Analysis B.I.-LISATM (Bruker BioSpin). Of note, results from this analysis were also used to determine TAG level in murine and human serum, as well as glucose concentrations in murine serum.

### Enzyme-linked immunosorbent assay (ELISA)

The serum C-peptide levels were measured using a C-peptide Enzyme Immunoassay Kit (RayBiotech, Norcross, USA), according to the manufacturer’s instructions.

For determination of insulin concentrations in serum the Mouse Insulin ELISA Kit (Invitrogen, Waltham, USA, #EMINS) was used, following the instructions from the manufacturer. Only exception, the sample and standard volume was reduced to 30 µl per well.

The serum level of arachidonic acid was measured using an Arachidonic Acid Immunoassay Kit (Abcam, Cambridge, UK, # ab287798), according to the manufacturer’s instructions.

To determine PGE_2_ concentrations undiluted supernatants of organoids were applied to the Human Prostaglandin E2 ELISA Kit (Invitrogen, #KHL1701), according to the manufacturer’s instructions.

For all ELISAs the absorbance was read, using a microplate reader (SpectraMax iD3, Molecular Devices, CA, USA).

### D-Glucose assay

D-glucose levels were measured using the D-Glucose Assay Kit (Megazyme, #K-GLUC). D-Glucose levels were measured in supernatants from organoid culture diluted in ddH_2_O. 5 µl of diluted sample, blank, and standards were added in duplicates into a 96-well plate. 150 µl GOPOD reagent was added to each well and the plate was incubated for 20 mi at 45 °C protected from light. Absorbance was read at 510 nm, using the SpectraMax iD3 (Molecular Devices).

### L-Lactate assay

L-lactate concentrations were determined in 1:20 (v/v) diluted organoid supernatants collected after 72 h incubation with 1 µM PMA (Invivogen) using the L-Lactate Assay Kit (Megazyme, #K-LATE), following the manufacturer’s protocol.

### Triglyceride assay

Glyceride levels in supernatants from organoid culture were measured using the Triglyceride Assay (Sigma Aldrich, #MAK266A). The assay was performed according to manufacturer’s protocol, however using only half of the recommended volume and half area plates (Corning, #CLS3690). After 45-min incubation samples were measured at 570 nm, using a microplate reader (SpectraMax iD3, Molecular Devices).

Triglyceride levels in plasma samples of the 12-week intervention animals were analysed by LC-MS, as published in a previous study [29].

### Statistics

Statistical analysis and data visualization was performed using the GraphPad Prism version 10.2.1 (San Diego, California, US). No statistical method was used to predetermine sample sizes. Outliers were identified by Grubbs’ test (significance level α = 0.05). The F test was used to compare variances and D’Agostino-Pearson test was applied to test for normal distribution. Statistical differences between two groups were analysed by unpaired t-test or paired t-test (normally distributed data), unpaired t-test with Welch’s correction (significantly different variances) or Mann-Whitney U-test (not-normally distributed data). Two-way analysis of variance (ANOVA) with Bonferroni’s, Dunnett’s, Šidák’s, Tukey’s, or Fisher’s least significant difference (LSD) post hoc test was applied for datasets with two variables. Correlation analysis was performed by obtaining the Spearman’s rank correlation coefficient. *P*-values were calculated, and null hypotheses were rejected when *P* ≤ 0.05. Depending on normal distribution, data are shown as median with range, as mean ± standard error of the mean (mean ± S.E.M.), or as mean ± standard deviation (mean ± S.D.).

Additionally, mice that died prematurely during the study or were removed from the experiment upon reaching predefined endpoints were excluded from the statistical analyses. Further, biological samples obtained from these mice, as well as any samples failing to meet predefined quality control criteria, were excluded from subsequent analyses.

## Supplementary information

Supplement

Uncropped Western Blots

## Data Availability

The bulk RNA-sequencing and microarray data generated during this study have been deposited in the Gene Expression Omnibus (GEO) repository under accession numbers GSE335477 and GSE335478, respectively. The study (reference number Az-21-415) is registered in the German Clinical Trials Register (DRKS) under the identifier DRKS00031028. Full-length and uncropped Western blots are provided in the Supplementary Material under “Uncropped Western Blots”.
