# Supplementary material for: Westernized diet related arachidonic acid metabolism expands DNA error-prone growth factor-dependent colonic quiescent stem cells in adolescent mice
Source: Cell Death Dis. 2026 Sep 14;17(1):798. doi: 10.1038/s41419-026-09240-9 (PMC13575188; doi:10.1038/s41419-026-09240-9)

Fig. 1k

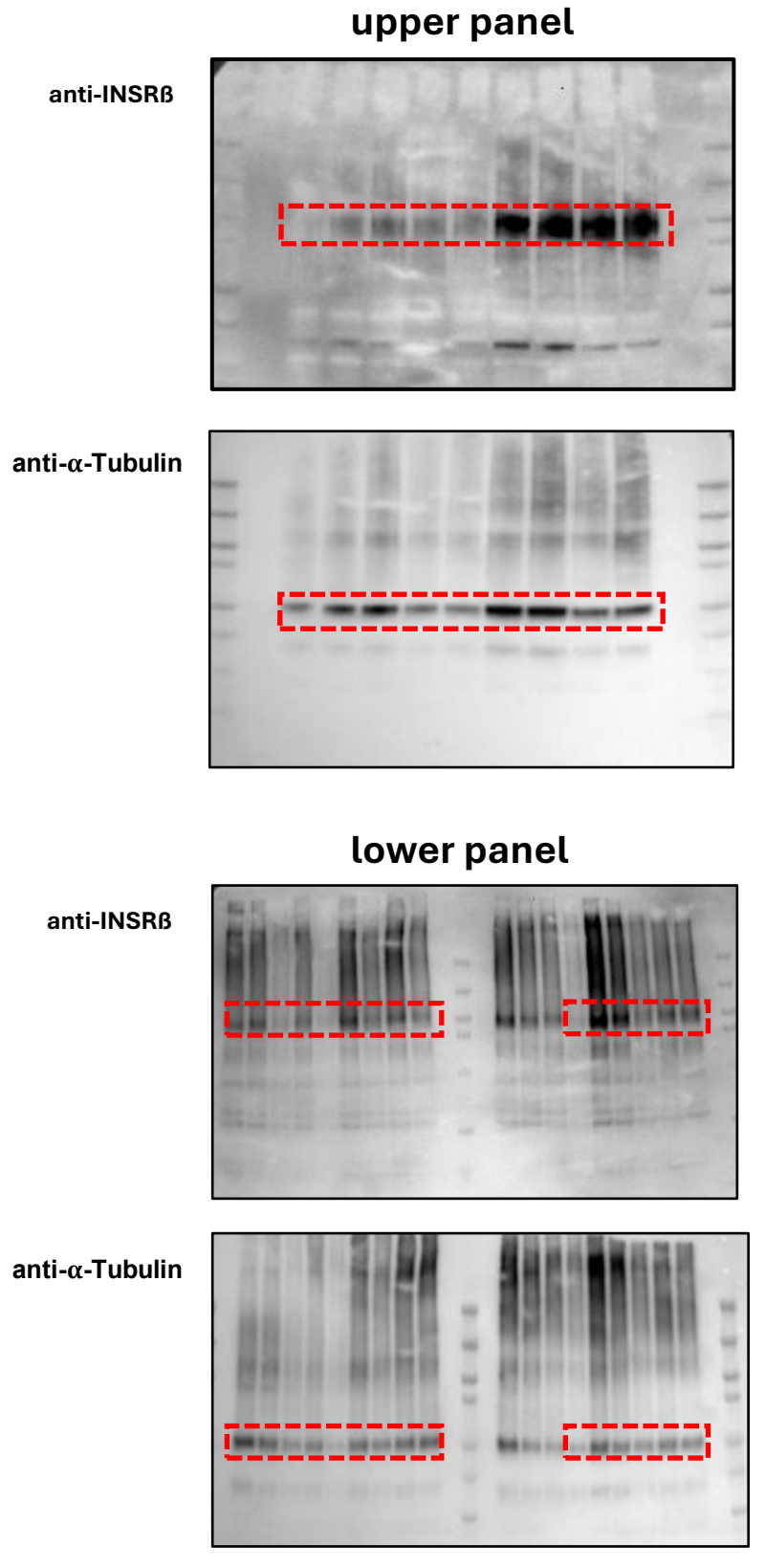

Fig. 1l

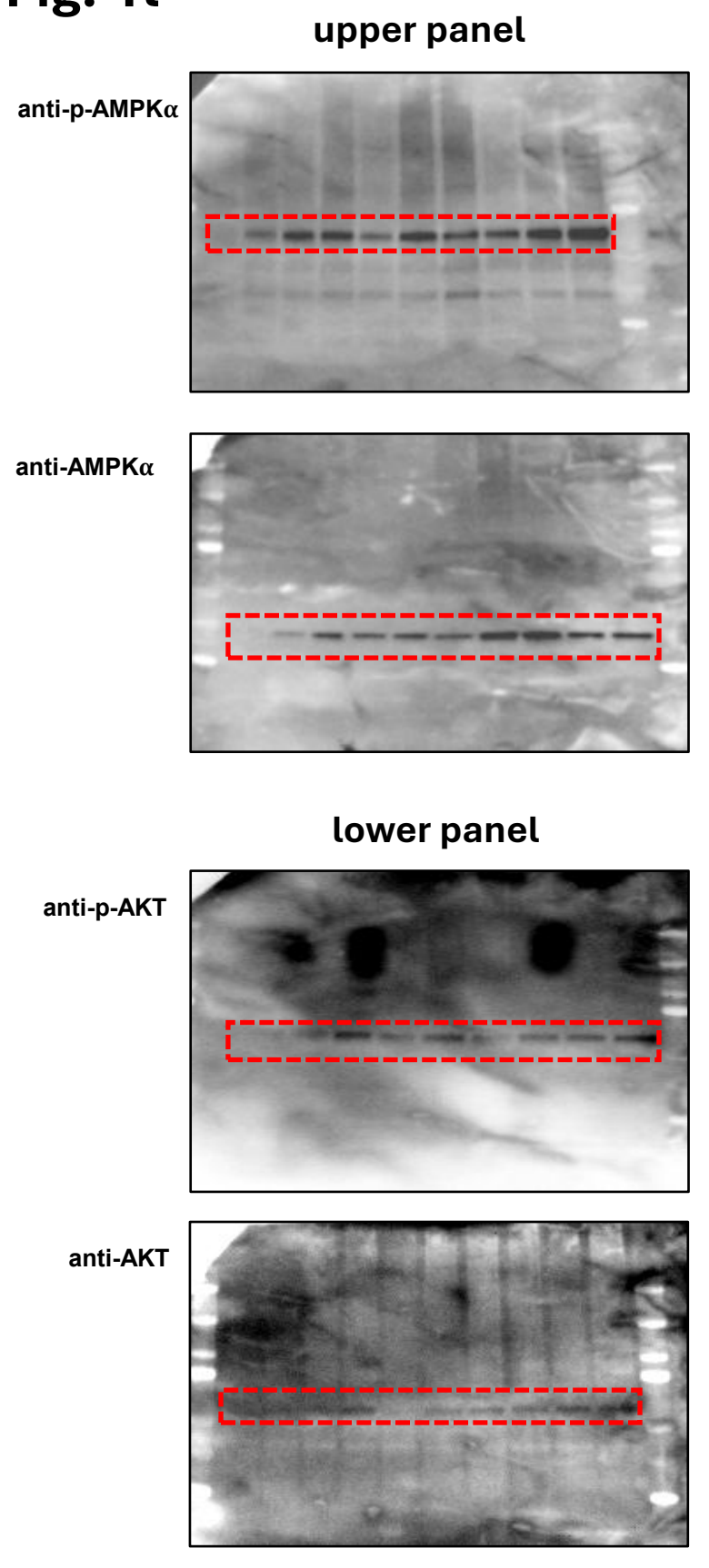

Fig. 1m

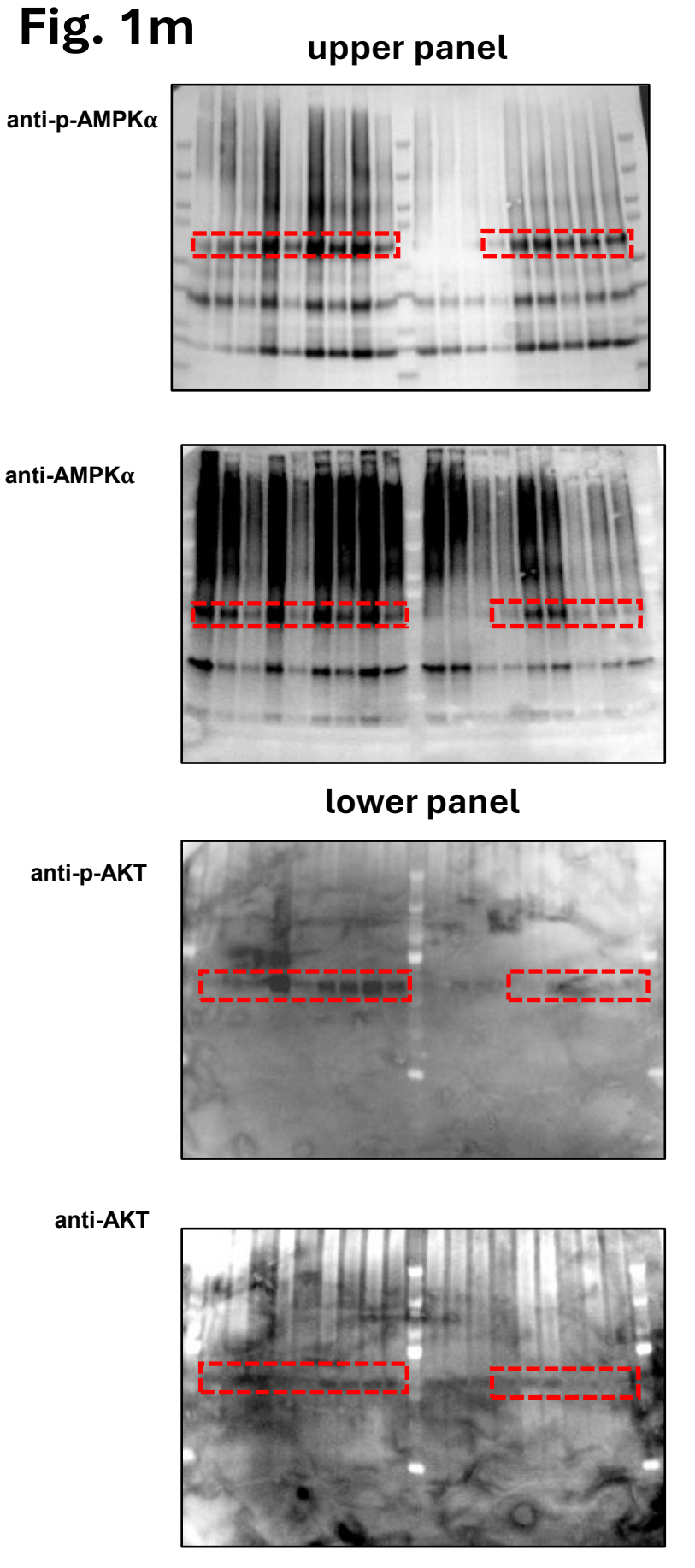

Fig. 2b

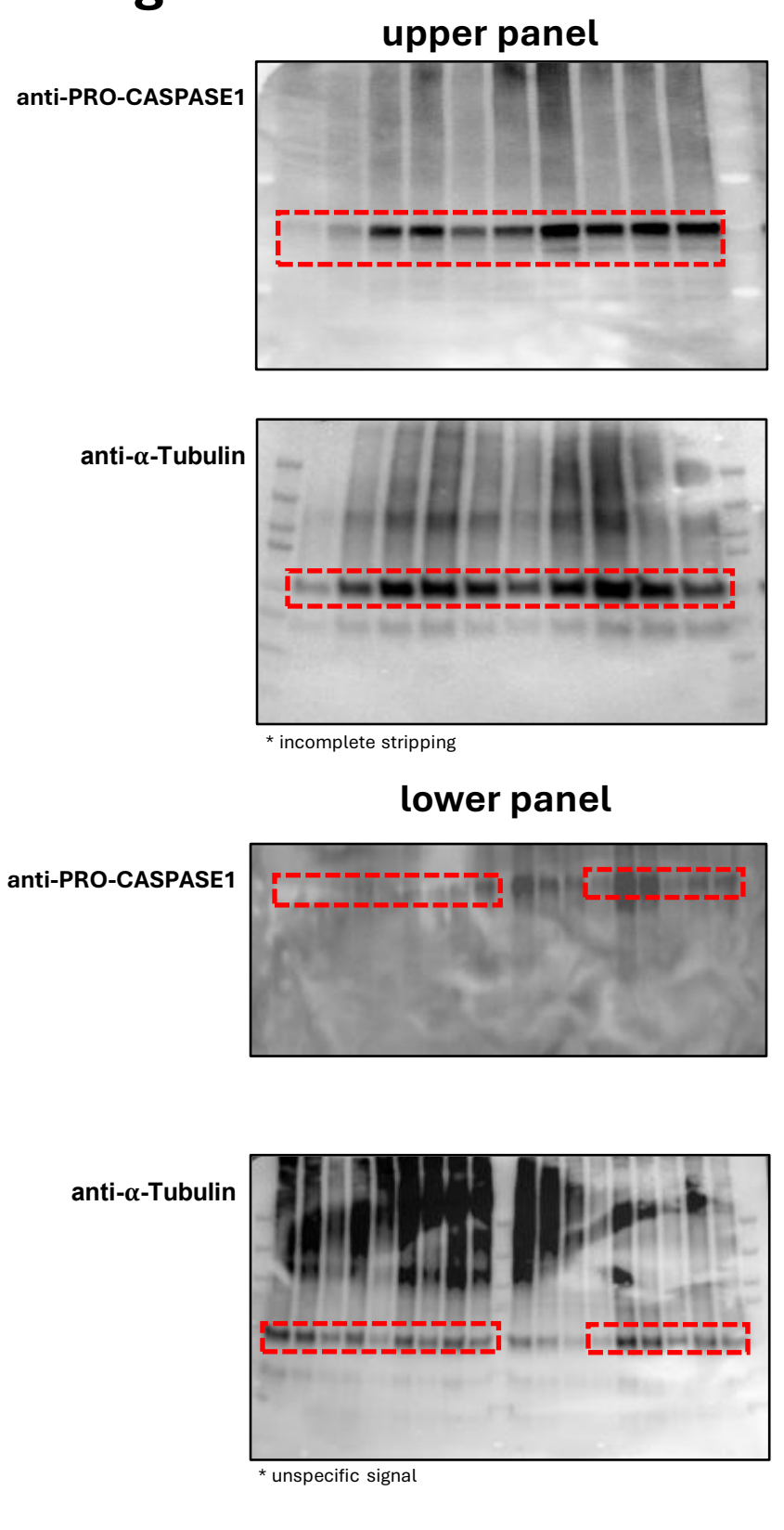

Fig. 5b

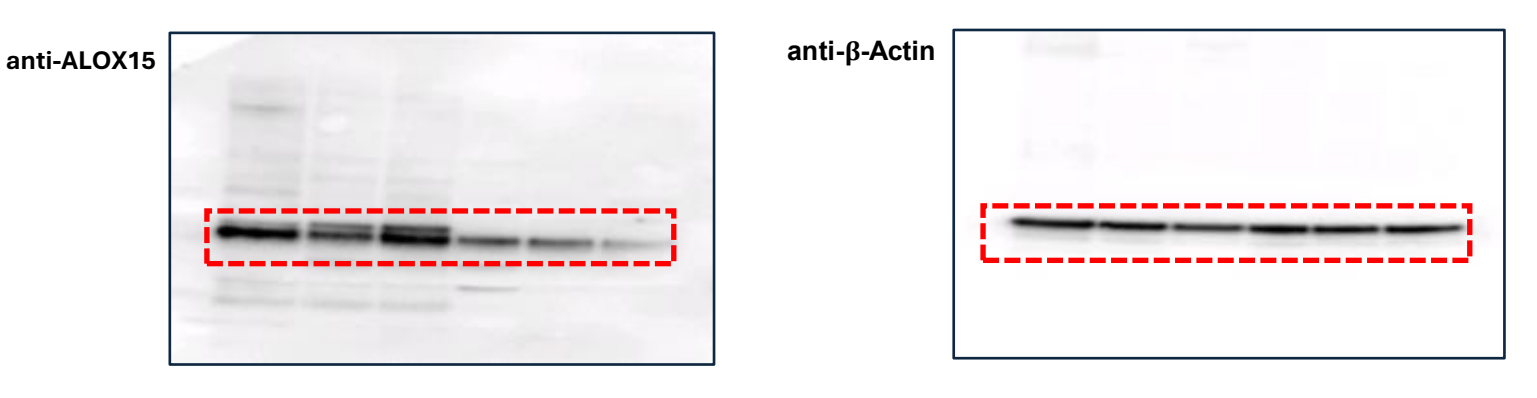

Fig. 6o

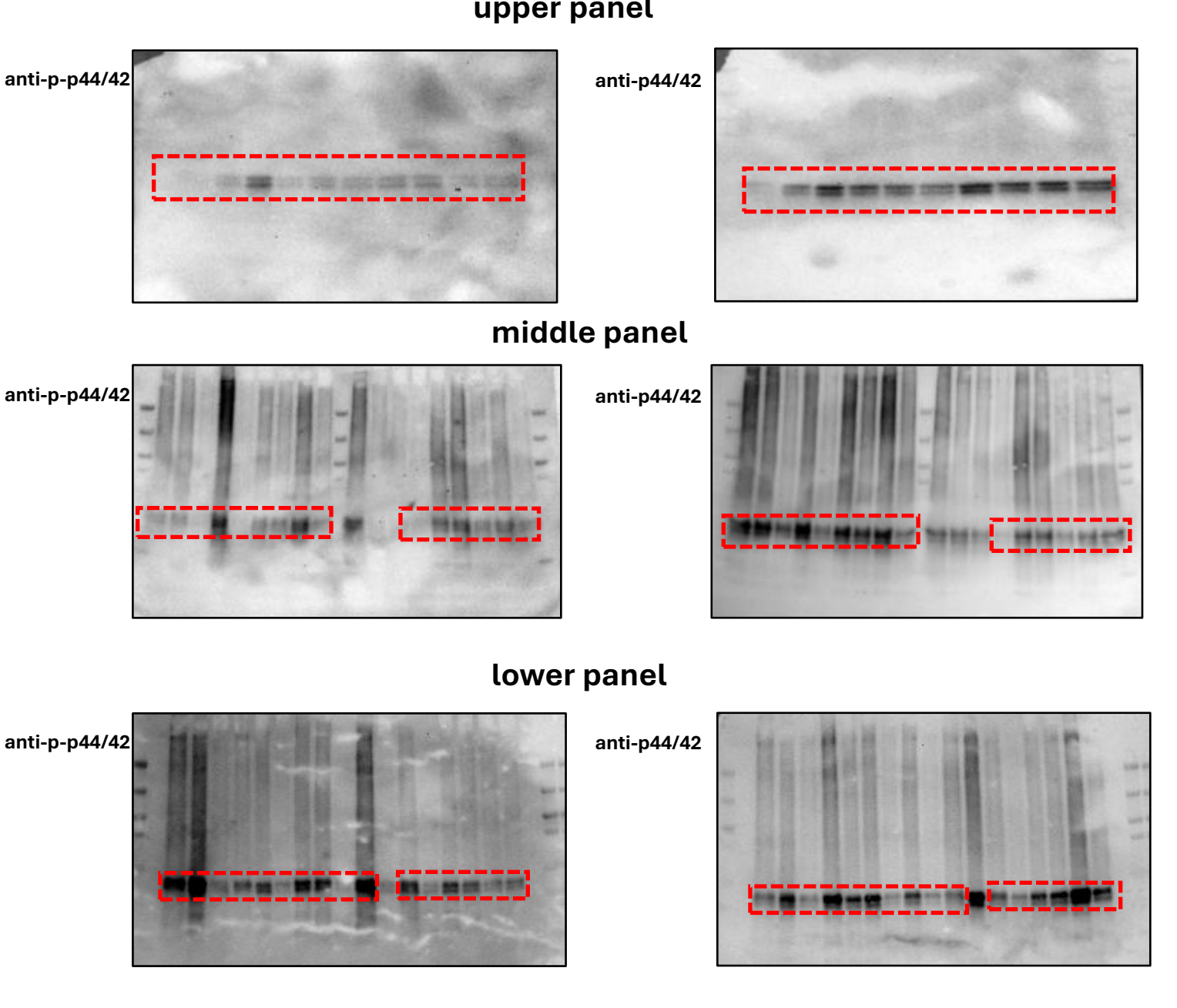

Fig. 7c

upper panel

anti-MSH2

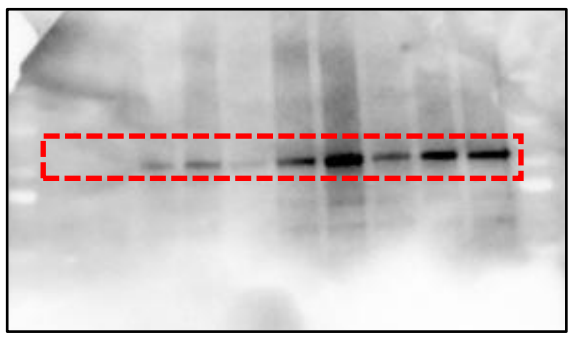

anti-α-Tubulin

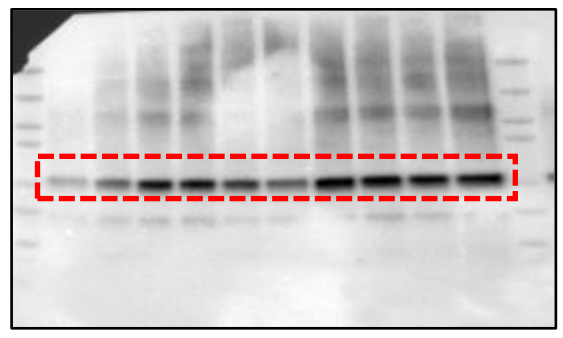

lower panel

anti-MSH2

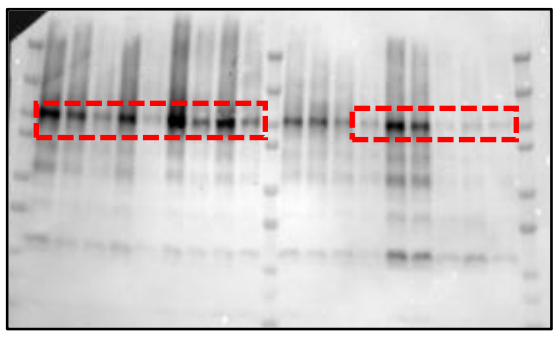

anti-α-Tubulin

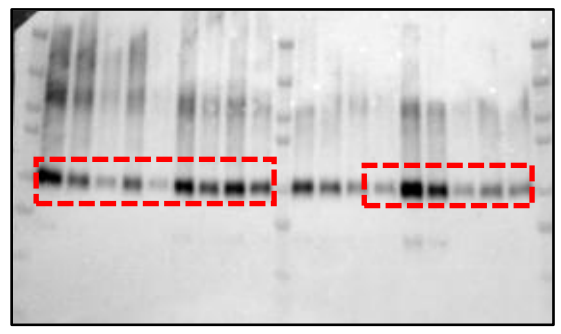

Fig. 7e

anti-MSH2

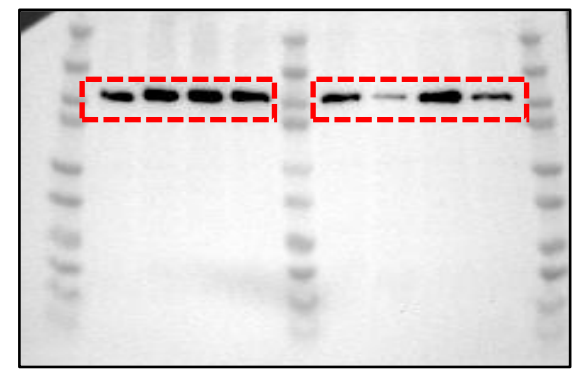

anti-α-Tubulin

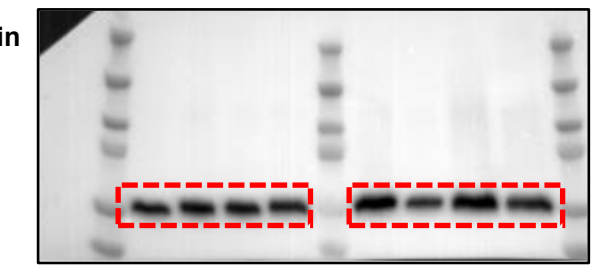

Supp. Fig. 2b

anti-INSRß

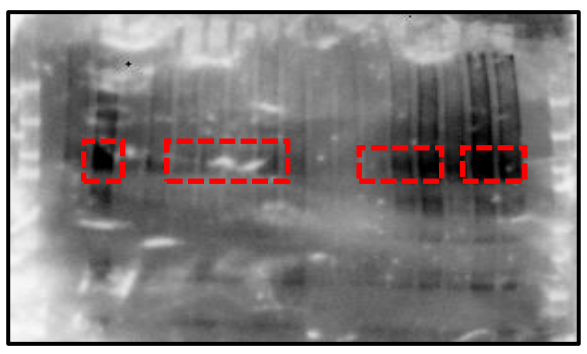

anti-α-Tubulin

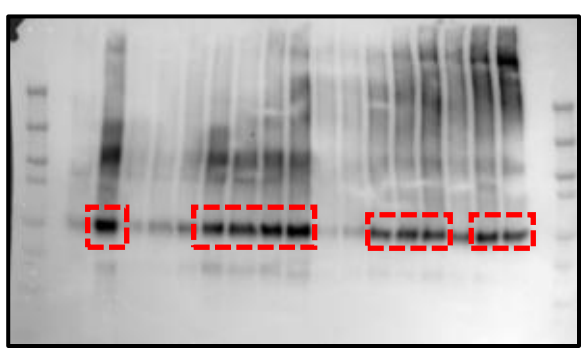

Supp. Fig. 2c

upper panel

anti-p-AMPKα

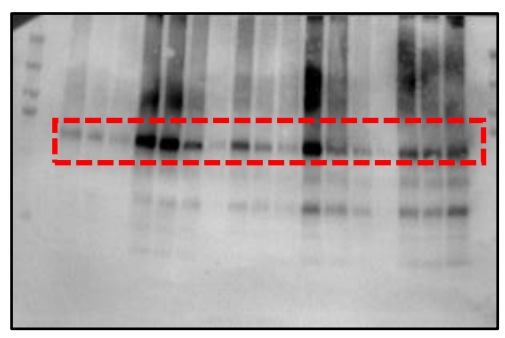

anti-p-AMPKα

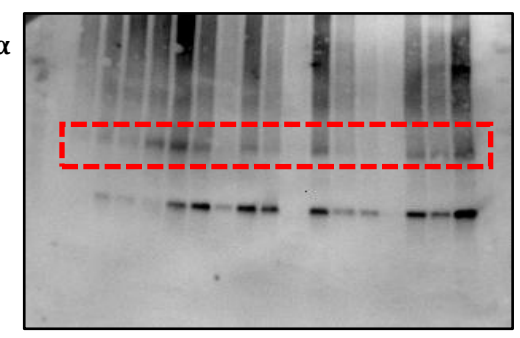

lower panel

anti-p-AMPKα

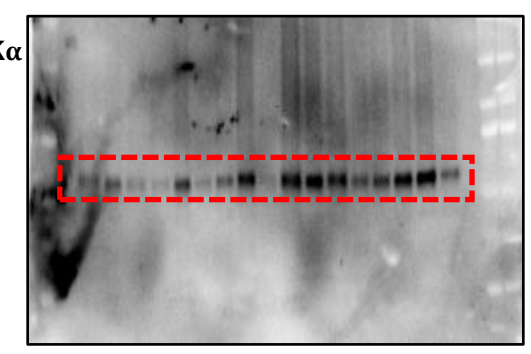

anti-p-AMPKα

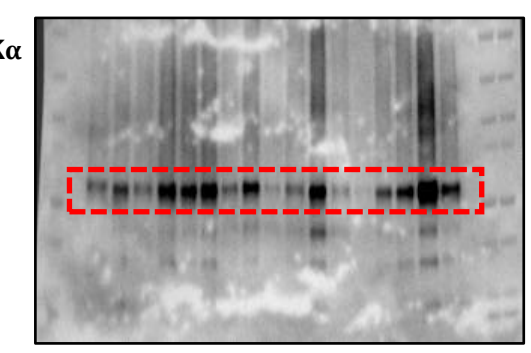

Supplement: Supplementary file 1 [file 41419_2026_9240_MOESM2_ESM.pdf]
