# Supplementary material for: Westernized diet related arachidonic acid metabolism expands DNA error-prone growth factor-dependent colonic quiescent stem cells in adolescent mice
Source: Cell Death Dis. 2026 Sep 14;17(1):798. doi: 10.1038/s41419-026-09240-9 (PMC13575188; doi:10.1038/s41419-026-09240-9)
Supplement: Supplementary file 2 [file 41419_2026_9240_MOESM1_ESM.docx]

**Supplementary Figure 1**

**Supplementary Fig. 1 | Characterisation of diet-induced alterations in cellular metabolism and differentiation. (a)** Heatmap of significantly downregulated lipid molecules in faecal samples of mice after 6 weeks of HFD (*n* = 4) in comparison to CD (*n* = 5). **(b)** Expression of *INSR* in HCEC-1CT cells (n = 3) stimulated with 10 *µ*M AA (n = 3) relative to DMSO-treated HCEC-1CT (Ctrl, *n* = 3). **(c)** Relative expression of *COX1* and *COX2* in HCEC-1CT cells (*n* = 4) stimulated with 10 *µ*M AA (*n* = 4) compared to DMSO-treated HCEC-1CT (Ctrl, *n* = 4). **(d)** Expression of PUFA-metabolising enzymes in PA (*n* = 3), OA (*n* = 4), or SA-treated HCEC-1CT (*n* = 4) relative to DMSO- (*n* = 3), H_2_O- (*n* = 4), or EtOH- (*n* = 4) cultured HCEC-1CT cells (Ctrl) using RT-qPCR. Relative expression of **(e)** *Cox1* and **(f)** *Cox2* in murine colonic tissue of HFD-fed mice compared to CD-fed littermates (6 weeks: HFD *n* = 6, CD *n* = 6; 12 weeks: HFD *n* = 5, CD *n* = 4 for *Cox1* and *n* = 3 for *Cox2*). **(g) Top:** Dot blot analysis using anti-MUC2 on colonic tissue from HFD- and CD-fed mice after 6 weeks (HFD n = 5, CD n = 4) and **bottom:** 12 weeks (each group *n* = 9), with Swift Membrane Stain™ as loading control. **(h)** Percentage [%] of *Klf4*⁺ colonic ECs across mouse colonic epithelial cells positive for *Lyz*, *Reg4*, *Bmi1*, and *Hes1*. Single-cell transcriptomics dataset of colonic healthy mouse tissue publicly available at Tabula “Muris Senis, Large Intestine - A single-cell transcriptomic atlas characterizing ageing tissues in the mouse (10x)”. **(i)** **Left:** *CASP1* and **right:** *IL1ß* expression in 10 µM AA (*n* = 3), 1 µM AA (*n* = 3) and 0.1 µM AA (*n* = 3) stimulated HCEC-1CT cells relative to Ctrl (0µM AA; *n* = 3). **(j) Left:** Transcriptional differentiation profile of PA-stimulated HCEC-1CT cells (*n* = 3) relative to DMSO-treated controls (Ctrl, *n* = 3); **Mid:** SA-stimulated HCEC-1CT cells (*n* = 4) relative to EtOH-treated controls (Ctrl, *n* = 4); **Right:** OA-stimulated HCEC-1CT cells (*n* = 4) relative to H_2_O-treated controls (Ctrl, *n* = 4). **(k)** *CDH1, EZR EPCAM ZO-1, OCLN, ITGB4, and CLDN1 expression* in human colonic organoids following stimulation with 10 µM AA (*n* = 5 independent PDOs) relative to paired DMSO-treated control organoids (Ctrl, *n* = 5 independent PDOs). **(l)** *CASP1* expression in human colonic organoids following stimulation with 10 *µ*M AA (*n* = 5 independent PDOs), normalized to paired DMSO-treated control organoids (Ctrl, *n* = 5 independent PDOs). **(m)** Cell type annotation colonic healthy mouse tissue and localisation of intestinal stem cell marker (*Lgr5*, *Cd44*, *Ascl2*), deep secretory cell marker (*Lyz*, *Reg4*, *Egf*), and quiescent intestinal stem cell marker (*Bmi1*, *Msi1*, *Hopx*), using Single-cell transcriptomics dataset of colonic healthy mouse tissue publicly available at Tabula “Muris Senis, Large Intestine - A single-cell transcriptomic atlas characterizing ageing tissues in the mouse (10x)”. **(n)** Relative *MSH2* expression of FA-treated HCEC-1CT in comparison to the respective control treatment (each group n = 4). Two-way ANOVA with **(a)**, **(c)**, **(e)**-**(f)**, **(i)**, **(n)** Fisher’s least significant difference (LSD) test or **(j)**-**(k)** Dunnett test for multiple comparison. **(l)** Wilcoxon matched pairs signed rank test. **(a)** Data shown as log-transformed individual data point. **(b)-(c)**, **(e)**-**(f)**, **(k)**, **(n)** Box plots depict the median and IQR with whiskers indicating the range. **(d)** Data depicted as median of log-transformed 2^-∆Ct^. **(h)** Parts of a whole [%]. **(i)**-**(j)** Data shown as median with error bar indicating the range. Data in **(b)**-**(d)**, **(i)**-**(j)** and **(n)** from independent biological replicates and **(a)**, **(e)**-**(f)** from at least three independent mice. **(b)**-**(c), (i)**-**(l)**, **(n)** Relative values were calculated by normalising to the paired control condition (set to 1) and **(e)**, **(f)** relative values were determined by normalisation to the median of the respective CD control group (set to 1). **P*≤0.05, ***P*≤0.01, *****P*≤0.0001. AA, arachidonic acid; EC, epithelial cell; FA, fatty acid; IQR, interquartile range; OA, oleic acid; PA, palmitic acid; PDO, patient-derived organoid; SA, steric acid; WD, western-style diet.

**Supplementary Figure 2**

**Supplementary Fig. 2 | Characterisation of WD- and CD-derived colonic murine organoids. (a)** IHC staining of murine colonic Ki67 after 24-week dietary intervention (WD n = 3 mice, CD n = 3 mice). **(b)** INSRß detection (WD *n* = 5 mice, CD *n* = 5 mice) and **(c)** phosphorylation status of AKT and AMPKα (WD *n* = 11 mice, CD *n* = 6 mice) in murine colonic tissue after 24-week dietary intervention, determined by Western blotting. **(d)** Representative brightfield microscopy image tracing of murine colonic organoid from WD- and CD-fed mice at day 0, day 3, day 5, day 7 and day 10 of basal cultivation. **(e)** Relative growth of WD- and CD-derived organoids following treatment with 1 µM PMA, normalised to timepoint 20 h and expressed relative to the respective paired control condition (WD-derived organoids, PMA/Ctrl: *n* = 3 independent organoids; CD-derived organoids, PMA/Ctrl: *n* = 3 independent organoids). **(f)** Normalised D-glucose concentration and **(g)** normalised L-lactate production in the supernatant of WD- and CD-derived organoids treated with 1 µM PMA or the respective paired control condition (WD-derived organoids, PMA/Ctrl: *n* = 3 independent organoids; CD-derived organoids, PMA/Ctrl: *n* = 3 independent organoids). **(h)** **Left:** Dot blot analysis of MUC2 in supernatants collected from WD- and CD-derived organoids treated for 72 h with 1 µM PMA or the respective control condition (WD-derived organoids, PMA/Ctrl: n = 3 independent organoids; CD-derived organoids, PMA/Ctrl: *n* = 3 independent organoids). **Right:** MUC2 abundance in the supernatant normalised to organoid biomass (AUC of relative growth) following 72 h treatment with 1 µM PMA or the respective control condition (WD-derived organoids, PMA/Ctrl: *n* = 3 independent organoids; CD-derived organoids, PMA/Ctrl: *n* = 3 independent organoids). **(a)** Mann Whitney *U* test. Two-way ANOVA using **(e)** Tukey, **(f)** Šidák correction for multiple testing, or **(h)-(i)** Fisher’s least significant difference (LSD) test. **(a)** Box plots depict the median and IQR with whiskers indicating the range. **(e)**-**(h)** Data shown as median ± range from three independent organoids per group. **(a)** Data from three different mice per group. *P≤0.05, **P≤0.01. #P≤0.05 (comparison between WD- or CD-derived organoid and paired control condition). AUC, area under the curve; CD, control diet; Ctrl, control condition; h, hour; IQR, interquartile range; PMA, Phorbol-12-myristate-13-acetate; WD, western-style diet.

**Supplementary Tables**

**Supplementary Table 1.** Diet composition as provided by the manufacturers.

| **Macronutrient** | **Autoclavable Rodent Diet 5010** | **D12450B** | **D12492i** | **D12331** | **Control (to Surwit)** |
| --- | --- | --- | --- | --- | --- |
| **Model** | CD, 6 weeks | CD, 12 weeks | HFD, 6 and 12 weeks | Surwit, 24 weeks | CD to Surwit, 24 weeks |
| Physiological fuel value | 3.430 kcal/g | 3.430 kcal/g | 5.240 kcal/g | 5.400 kcal/g | 3.695 kcal/g |
| Protein | 30 kcal% | 20 kcal% | 20 kcal% | 15 kcal% | 22 kcal% |
| Fat | 10 kcal% | 10 kcal% | 60 kcal% | 59 kcal% | 13 kcal% |
| Carbohydrates | 60 kcal% | 70 kcal% | 20 kcal% | 25 kcal% | 65 kcal% |

**Supplementary Table 2.** Results of statistical analysis serum 1H-NMR profiles (Multiple Mann-Whitney test, HFD *vs*. CD).

| **Metabolite** | **Below  threshold?** | ***P-*value** | **Mean rank HFD** | **Mean rank CD** | **Mean rank  diff.** | **Mann-Whitney U** |
| --- | --- | --- | --- | --- | --- | --- |
| Ethanol | Yes | 0.0065 | 3.75 | 9.25 | -5.50 | 1.50 |
| Trimethylamine-N-oxide | No | 0.2489 | 5.25 | 7.75 | -2.50 | 10.50 |
| Alanine | No | 0.6667 | 7.00 | 6.00 | 1.00 | 15.00 |
| Creatine | No | 0.1472 | 4.92 | 8.08 | -3.17 | 8.50 |
| Creatinine | No | 0.3593 | 7.58 | 5.42 | 2.17 | 11.50 |
| Glutamic acid | No | 0.7381 | 6.08 | 6.92 | -0.83 | 15.50 |
| Glutamine | No | 0.4589 | 5.67 | 7.33 | -1.67 | 13.00 |
| Glycine | No | 0.9740 | 6.42 | 6.58 | -0.17 | 17.50 |
| Histidine | No | 0.9784 | 6.58 | 6.42 | 0.17 | 17.50 |
| Isoleucine | No | 0.8571 | 6.25 | 6.75 | -0.50 | 16.50 |
| Leucine | No | 0.7251 | 6.08 | 6.92 | -0.83 | 15.50 |
| Lysine | No | 0.9134 | 6.67 | 6.33 | 0.33 | 17.00 |
| Methionine | No | 1.0000 | 6.50 | 6.50 | 0.00 | 18.00 |
| Ornithine | No | 0.1385 | 8.08 | 4.92 | 3.17 | 8.50 |
| Phenylalanine | No | 0.2035 | 5.08 | 7.92 | -2.83 | 9.50 |
| Proline | No | 0.6970 | 6.83 | 6.17 | 0.67 | 16.00 |
| Tyrosine | No | 0.3398 | 7.58 | 5.42 | 2.17 | 11.50 |
| Valine | No | 0.9697 | 6.42 | 6.58 | -0.17 | 17.50 |
| 2-Hydroxybutyric acid | No | 0.4545 | 7.50 | 5.50 | 2.00 | 12.00 |
| Acetic acid | Yes | 0.0022 | 3.50 | 9.50 | -6.00 | 0.00 |
| Citric acid | No | 0.3312 | 7.58 | 5.42 | 2.17 | 11.50 |
| Formic acid | No | 0.6926 | 7.17 | 5.83 | 1.33 | 14.00 |
| Lactic acid | Yes | 0.0087 | 3.83 | 9.17 | -5.33 | 2.00 |
| Succinic acid | No | 0.7879 | 6.83 | 6.17 | 0.67 | 16.00 |
| Choline | No | 0.0909 | 8.33 | 4.67 | 3.67 | 7.00 |
| 2-Oxoglutaric acid | No | 1.0000 | 6.58 | 6.42 | 0.17 | 17.50 |
| 3-Hydroxybutyric acid | No | 0.9177 | 6.33 | 6.67 | -0.33 | 17.00 |
| Acetone | No | 1.0000 | 6.33 | 6.67 | -0.33 | 17.00 |
| Pyruvic acid | No | 0.5173 | 5.75 | 7.25 | -1.50 | 13.50 |
| Glucose | Yes | 0.0065 | 9.33 | 3.67 | 5.67 | 1.00 |
| Glycerol | No | 0.9372 | 6.33 | 6.67 | -0.33 | 17.00 |
| TAG | No | 0.8182 | 6.83 | 6.17 | 0.67 | 16.00 |
| Cholesterol | Yes | 0.0022 | 9.50 | 3.50 | 6.00 | 0.00 |

1H-NMR, 1H-nucelar magnetic resonance; HFD, high-fat diet; CD, control diet

**Supplementary Table 3.** Results of statistical analysis of faecal lipidome (Multiple Mann-Whitney test; CD *vs.* HFD).

| **Lipid** | **Below  threshold?** | ***P-*value** | **Mean rank CD** | **Mean rank HFD** | **Mean rank  diff.** | **Mann-Whitney U** |
| --- | --- | --- | --- | --- | --- | --- |
| AcCa (16:0) | Yes | 0.0159 | 3.00 | 7.50 | -4.50 | 0.00 |
| AcCa (18:0) | Yes | 0.0159 | 3.00 | 7.50 | -4.50 | 0.00 |
| AcCa (18:1) | No | 0.1905 | 3.80 | 6.50 | -2.70 | 4.00 |
| AcCa (24:0) | Yes | 0.0159 | 3.00 | 7.50 | -4.50 | 0.00 |
| CE (18:1) | Yes | 0.0159 | 7.00 | 2.50 | 4.50 | 0.00 |
| CE (20:4) | Yes | 0.0317 | 3.20 | 7.25 | -4.05 | 1.00 |
| CE (22:6) | Yes | 0.0317 | 6.80 | 2.75 | 4.05 | 1.00 |
| Cer (d32:0) | Yes | 0.0159 | 3.00 | 7.50 | -4.50 | 0.00 |
| Cer (d34:0) | Yes | 0.0159 | 3.00 | 7.50 | -4.50 | 0.00 |
| Cer (d34:1) | Yes | 0.0159 | 7.00 | 2.50 | 4.50 | 0.00 |
| Cer (d36:0) | Yes | 0.0317 | 3.20 | 7.25 | -4.05 | 1.00 |
| Cer (d36:1) | Yes | 0.0079 | 3.00 | 7.50 | -4.50 | 0.00 |
| Cer (d38:0) | No | 0.2857 | 4.00 | 6.25 | -2.25 | 5.00 |
| Cer (d40:0) | Yes | 0.0159 | 3.00 | 7.50 | -4.50 | 0.00 |
| Cer (d41:0) | No | 0.1905 | 3.80 | 6.50 | -2.70 | 4.00 |
| Cer (d44:0) | No | 0.4127 | 4.20 | 6.00 | -1.80 | 6.00 |
| CerG (d36:2) | Yes | 0.0159 | 3.00 | 7.50 | -4.50 | 0.00 |
| CerG (d40:0) | Yes | 0.0159 | 3.00 | 7.50 | -4.50 | 0.00 |
| CerG (d40:2) | Yes | 0.0159 | 3.00 | 7.50 | -4.50 | 0.00 |
| CerG (d41:2) | Yes | 0.0159 | 3.00 | 7.50 | -4.50 | 0.00 |
| CerG (d42:2) | Yes | 0.0317 | 3.20 | 7.25 | -4.05 | 1.00 |
| SA (C17:0) | No | 0.1111 | 6.40 | 3.25 | 3.15 | 3.00 |
| SA (C18:0) | No | 0.9048 | 5.20 | 4.75 | 0.45 | 9.00 |
| SA (C19:0) | Yes | 0.0159 | 7.00 | 2.50 | 4.50 | 0.00 |
| DAG (34:1) | No | 0.9048 | 4.80 | 5.25 | -0.45 | 9.00 |
| DAG (36:3) | Yes | 0.0159 | 7.00 | 2.50 | 4.50 | 0.00 |
| DAG (36:4) | Yes | 0.0159 | 7.00 | 2.50 | 4.50 | 0.00 |
| DAG (40:7) | Yes | 0.0159 | 7.00 | 2.50 | 4.50 | 0.00 |
| FA (16:0) | Yes | 0.0159 | 3.00 | 7.50 | -4.50 | 0.00 |
| FA (18:0) | Yes | 0.0159 | 3.00 | 7.50 | -4.50 | 0.00 |
| FA (18:1) | Yes | 0.0159 | 3.00 | 7.50 | -4.50 | 0.00 |
| FA (18:2) | No | 0.0635 | 3.40 | 7.00 | -3.60 | 2.00 |
| FA (20:0) | Yes | 0.0159 | 3.00 | 7.50 | -4.50 | 0.00 |
| FA (20:1) | Yes | 0.0159 | 3.00 | 7.50 | -4.50 | 0.00 |
| FA (20:4) | Yes | 0.0159 | 3.00 | 7.50 | -4.50 | 0.00 |
| FA (22:4) | No | 0.2857 | 4.20 | 6.00 | -1.80 | 6.00 |
| FA (22:5) | No | 0.1905 | 3.80 | 6.50 | -2.70 | 4.00 |
| FA (22:6) | No | 0.9048 | 5.20 | 4.75 | 0.45 | 9.00 |
| FA (24:0) | No | 0.9048 | 4.80 | 5.25 | -0.45 | 9.00 |
| FA (24:1) | Yes | 0.0079 | 3.00 | 7.50 | -4.50 | 0.00 |
| LPC (16:0) | Yes | 0.0159 | 3.00 | 7.50 | -4.50 | 0.00 |
| LPC (17:0) | Yes | 0.0159 | 3.00 | 7.50 | -4.50 | 0.00 |
| LPC (18:0) | Yes | 0.0159 | 3.00 | 7.50 | -4.50 | 0.00 |
| LPC (18:1) | No | 0.0635 | 3.40 | 7.00 | -3.60 | 2.00 |
| LPC (18:2) | No | 0.9048 | 5.20 | 4.75 | 0.45 | 9.00 |
| LPC (20:0) | Yes | 0.0159 | 3.00 | 7.50 | -4.50 | 0.00 |
| LPC (20:4) | Yes | 0.0159 | 3.00 | 7.50 | -4.50 | 0.00 |
| LPC (22:0) | Yes | 0.0159 | 7.00 | 2.50 | 4.50 | 0.00 |
| LPC (22:1) | Yes | 0.0159 | 3.00 | 7.50 | -4.50 | 0.00 |
| LPC (24:0) | Yes | 0.0159 | 7.00 | 2.50 | 4.50 | 0.00 |
| LPC (24:1) | No | 0.9048 | 5.20 | 4.75 | 0.45 | 9.00 |
| LPI (18:0) | Yes | 0.0159 | 3.00 | 7.50 | -4.50 | 0.00 |
| DeoxySA (C18:0) | Yes | 0.0317 | 6.80 | 2.75 | 4.05 | 1.00 |
| Deoxysphingosine (C18:1) | No | 1.0000 | 5.00 | 5.00 | 0.00 | 10.00 |
| Oleoyl-EA (C18:1) | Yes | 0.0159 | 7.00 | 2.50 | 4.50 | 0.00 |
| PC (30:0) | Yes | 0.0159 | 3.00 | 7.50 | -4.50 | 0.00 |
| PC (31:0) | Yes | 0.0317 | 3.20 | 7.25 | -4.05 | 1.00 |
| PC (32:1) | Yes | 0.0159 | 3.00 | 7.50 | -4.50 | 0.00 |
| PC (33:0) | No | 0.2063 | 3.90 | 6.38 | -2.48 | 4.50 |
| PC (34:1) | Yes | 0.0159 | 3.00 | 7.50 | -4.50 | 0.00 |
| PC (34:2) | Yes | 0.0159 | 3.00 | 7.50 | -4.50 | 0.00 |
| PC (36:1) | Yes | 0.0159 | 3.00 | 7.50 | -4.50 | 0.00 |
| PC (36:2) | Yes | 0.0159 | 3.00 | 7.50 | -4.50 | 0.00 |
| PC (36:4) | Yes | 0.0159 | 3.00 | 7.50 | -4.50 | 0.00 |
| PC (38:4) | Yes | 0.0159 | 3.00 | 7.50 | -4.50 | 0.00 |
| PC (38:5) | Yes | 0.0159 | 3.00 | 7.50 | -4.50 | 0.00 |
| PC (38:6) | Yes | 0.0159 | 3.00 | 7.50 | -4.50 | 0.00 |
| PC (40:6) | Yes | 0.0159 | 3.00 | 7.50 | -4.50 | 0.00 |
| PC (40:8) | Yes | 0.0159 | 3.00 | 7.50 | -4.50 | 0.00 |
| PE (31:0) | No | 0.1905 | 6.20 | 3.50 | 2.70 | 4.00 |
| PE (32:0) | No | 0.9048 | 4.80 | 5.25 | -0.45 | 9.00 |
| PE (32:1) | No | 0.5556 | 4.40 | 5.75 | -1.35 | 7.00 |
| PE (34:1) | Yes | 0.0159 | 3.00 | 7.50 | -4.50 | 0.00 |
| PE (36:2) | No | 0.5556 | 5.60 | 4.25 | 1.35 | 7.00 |
| Palmitoyl-EA (C16:0) | Yes | 0.0159 | 7.00 | 2.50 | 4.50 | 0.00 |
| SM (d32:0) | Yes | 0.0159 | 3.00 | 7.50 | -4.50 | 0.00 |
| SM (d32:1) | Yes | 0.0159 | 3.00 | 7.50 | -4.50 | 0.00 |
| SM (d34:0) | Yes | 0.0159 | 3.00 | 7.50 | -4.50 | 0.00 |
| SM (d34:1) | Yes | 0.0159 | 3.00 | 7.50 | -4.50 | 0.00 |
| SM (d34:2) | Yes | 0.0159 | 3.00 | 7.50 | -4.50 | 0.00 |
| SM (d36:0) | Yes | 0.0159 | 3.00 | 7.50 | -4.50 | 0.00 |
| SM (d36:1) | Yes | 0.0159 | 3.00 | 7.50 | -4.50 | 0.00 |
| SM (d38:0) | Yes | 0.0159 | 3.00 | 7.50 | -4.50 | 0.00 |
| SM (d38:1) | Yes | 0.0159 | 3.00 | 7.50 | -4.50 | 0.00 |
| SM (d39:1) | Yes | 0.0159 | 3.00 | 7.50 | -4.50 | 0.00 |
| SM (d40:0) | Yes | 0.0159 | 3.00 | 7.50 | -4.50 | 0.00 |
| SM (d40:1) | Yes | 0.0159 | 3.00 | 7.50 | -4.50 | 0.00 |
| SM (d40:2) | No | 0.2857 | 4.00 | 6.25 | -2.25 | 5.00 |
| SM (d41:0) | Yes | 0.0159 | 3.00 | 7.50 | -4.50 | 0.00 |
| SM (d41:1) | Yes | 0.0159 | 3.00 | 7.50 | -4.50 | 0.00 |
| SM (d42:0) | Yes | 0.0159 | 3.00 | 7.50 | -4.50 | 0.00 |
| SM (d42:1) | Yes | 0.0159 | 3.00 | 7.50 | -4.50 | 0.00 |
| SM (d42:2) | No | 0.5556 | 4.40 | 5.75 | -1.35 | 7.00 |
| SM (d42:3) | No | 0.1905 | 3.80 | 6.50 | -2.70 | 4.00 |
| SM (d43:3) | No | 0.0556 | 6.60 | 3.00 | 3.60 | 2.00 |
| Phytosphingosine (C18:0) | No | 0.9048 | 5.20 | 4.75 | 0.45 | 9.00 |
| TAG (48:1) | No | 0.4127 | 4.20 | 6.00 | -1.80 | 6.00 |
| TAG (51:1) | Yes | 0.0159 | 3.00 | 7.50 | -4.50 | 0.00 |
| TAG (52:1) | Yes | 0.0159 | 3.00 | 7.50 | -4.50 | 0.00 |
| TAG (54:1) | Yes | 0.0159 | 3.00 | 7.50 | -4.50 | 0.00 |
| TAG (54:6) | Yes | 0.0317 | 6.80 | 2.75 | 4.05 | 1.00 |
| TAG (54:7) | Yes | 0.0159 | 7.00 | 2.50 | 4.50 | 0.00 |
| TAG (56:0) | Yes | 0.0159 | 3.00 | 7.50 | -4.50 | 0.00 |
| TAG (56:3) | No | 0.2857 | 6.00 | 3.75 | 2.25 | 5.00 |

HFD, high-fat diet; CD, control diet

|  | **Tissue** | **Male (n)** | **Female**  **(n)** | **Sex  un-known (n)** | **Median age (Min/ Max)**  **(y)** | **TNM** | **Tumour**  **tage** | **Tumour grade (n)** | **Tumour cells [%]** |
| --- | --- | --- | --- | --- | --- | --- | --- | --- | --- |
| Paired samples from CRC patients for **Figure 3m + 7i** (OriGene) | normal | 5 | 5 | 0 | 74.5 (65/89) | n.a. | n.a. | n.a. | n.a. |
|  | tumour | 5 | 5 | 0 |  | pT3pN0pMX | IIA (10) | G1 (3) G2 (7) | 60-89 |
| Tissue Scan Colon Cancer tissue qPCR Panel *III* Matched pairs for **Figure 7j** (OriGene, #HCRT10303) | normal | 11 | 13 | 0 | 76 (21/89) | n.a. | n.a. | n.a. | n.a. |
|  | tumour | 11 | 13 | 0 |  | pT2pN0pMX  pT1pN0pMX  pT3pN0pMX  pT4pN0pMX  pTXpN1pMX  pT1pN1pMX  pT3pN1pMX  pT4pN2pMX  pT3pNXpM1 | I (5), II (7), III (8), IV (4) | n.a. (2)  G10 (10), G2 (13), G3 (1) | 40-95 |
| Human colonic biopsies of hospitalized normal patients for qPCR  (Az-19-233; Az-2023-172; Az-05-112) | normal | 9 | 9 | 1 | 70  (26/85) | n.a. | n.a. | n.a. | n.a. |
| Healthy colonic biopsy donors for organoid generation (Az-21-415) | normal | 2 | 3 | 0 | 34 (20/58) | n.a. | n.a. | n.a. | n.a. |
| nonCRC serum donors (Az-21-415) | n.a. | 22 | 39 | 0 | 45  (20/79) | n.a. | n.a. | n.a. | n.a. |
| CRC serum donors  (Az-16-282) | n.a. | 40 | 24 | 0 | 70 (21/89) | n.a. | I (11),  II (18),  III (14),  IV (2),  n.a. (38) | n.a. | n.a. |

**Supplementary Table 4.** Patient characteristics of tissue and serum samples.

n.a., not applicable/not available

**Supplementary Table 5.** Clinical data of nonCRC cohort used to determine serum triglyceride (TG) level.

| **Sample size, n**  (total/female/male) | **BMI**  Median (Min/Max) | **Dietary background**  (omnivore/vegetarian/vegan/n.a.) | **Comorbidities**  (IBS/allergic food intolerance/ Celiac disease/SIBO/ Hypertension /Diabetes Type I/ Diabetes Type II/n.a.) |
| --- | --- | --- | --- |
| 61/39/22 | 23.82 (17.28/37.92) | 51/6/2/2 | 7/8/3/5/3/1/1/30 |

BMI, Body-Mass-Index, IBS, Irritable Bowel Syndrome; not applicable/not available; SIBO, Small Intestinal Bacterial Overgrowth

**Supplementary Table 6.** Statistical analysis of grouped marker-positive cell populations (Twoway ANOVA with Fishers LSD test; ISC vs. DSC vs. qISC).

| **Uncorrected Fishers LSD test** | **Predicted (LS) Mean diff.** | **95,00% CI of diff.** | **Adjusted P Value** |
| --- | --- | --- | --- |
| ***APOBR*** | | | |
| ISC vs. DSC | -1.885 | -13,50 to 9,730 | 0.7497 |
| ISC vs. qISC | -16.81 | -25,47 to -8,151 | 0.0002 |
| DSC vs. qISC | -14.92 | -25,87 to -3,973 | 0.0077 |
| ***BMPR1A*** |  |  |  |
| ISC vs. DSC | -0.505 | -12,12 to 11,11 | 0.9319 |
| ISC vs. qISC | -8.188 | -16,85 to 0,4687 | 0.0637 |
| DSC vs. qISC | -7.683 | -18,63 to 3,267 | 0.1684 |
| ***BMPR1B*** |  |  |  |
| ISC vs. DSC | -1.066E-14 | -11,61 to 11,61 | >0,9999 |
| ISC vs. qISC | -0.008333 | -8,665 to 8,649 | 0.9985 |
| DSC vs. qISC | -0.008333 | -10,96 to 10,94 | 0.9988 |
| ***BMPR2*** |  |  |  |
| ISC vs. DSC | 4.11 | -7,505 to 15,72 | 0.4867 |
| ISC vs. qISC | -5.512 | -14,17 to 3,145 | 0.2112 |
| DSC vs. qISC | -9.622 | -20,57 to 1,329 | 0.0848 |
| ***EGFR*** | | | |
| ISC vs. DSC | 7.3 | -4,315 to 18,91 | 0.2171 |
| ISC vs. qISC | -10.1 | -18,76 to -1,443 | 0.0224 |
| DSC vs. qISC | -17.4 | -28,35 to -6,450 | 0.0019 |
| ***EPHA2*** | | | |
| ISC vs. DSC | 1.675 | -9,940 to 13,29 | 0.7768 |
| ISC vs. qISC | -5.288 | -13,95 to 3,369 | 0.2303 |
| DSC vs. qISC | -6.963 | -17,91 to 3,987 | 0.2118 |
| ***EPHB2*** | | | |
| ISC vs. DSC | 21.72 | 10,11 to 33,33 | 0.0003 |
| ISC vs. qISC | 1.54 | -7,117 to 10,20 | 0.7265 |
| DSC vs. qISC | -20.18 | -31,13 to -9,230 | 0.0003 |
| ***ERBB2*** | | | |
| ISC vs. DSC | 6.615 | -5,000 to 18,23 | 0.2633 |
| ISC vs. qISC | -9.193 | -17,85 to -0,5363 | 0.0375 |
| DSC vs. qISC | -15.81 | -26,76 to -4,858 | 0.0048 |
| ***ERBB3*** | | | |
| ISC vs. DSC | 8.68 | -2,935 to 20,29 | 0.1424 |
| ISC vs. qISC | -16.41 | -25,07 to -7,755 | 0.0002 |
| DSC vs. qISC | -25.09 | -36,04 to -14,14 | <0,0001 |
| ***ERBB4*** | | | |
| ISC vs. DSC | 0.0275 | -11,59 to 11,64 | 0.9963 |
| ISC vs. qISC | 0.01917 | -8,638 to 8,676 | 0.9965 |
| DSC vs. qISC | -0.008333 | -10,96 to 10,94 | 0.9988 |
| ***FGFR1*** | | | |
| ISC vs. DSC | 1.3 | -10,31 to 12,91 | 0.8258 |
| ISC vs. qISC | 0.8517 | -7,805 to 9,509 | 0.8466 |
| DSC vs. qISC | -0.4483 | -11,40 to 10,50 | 0.9358 |
| ***FGFR2*** | | | |
| ISC vs. DSC | 2.113 | -9,502 to 13,73 | 0.7206 |
| ISC vs. qISC | -0.6792 | -9,336 to 7,978 | 0.8774 |
| DSC vs. qISC | -2.792 | -13,74 to 8,159 | 0.6162 |
| ***FGFR3*** | | | |
| ISC vs. DSC | 1.593 | -10,02 to 13,21 | 0.7875 |
| ISC vs. qISC | -3.836 | -12,49 to 4,821 | 0.3839 |
| DSC vs. qISC | -5.428 | -16,38 to 5,522 | 0.3301 |
| ***FGFR4*** | | | |
| ISC vs. DSC | 9.013 | -2,602 to 20,63 | 0.1278 |
| ISC vs. qISC | -1.406 | -10,06 to 7,251 | 0.7495 |
| DSC vs. qISC | -10.42 | -21,37 to 0,5321 | 0.0621 |
| ***IGF1R*** | | | |
| ISC vs. DSC | 5.74 | -5,875 to 17,35 | 0.3316 |
| ISC vs. qISC | -1.255 | -9,912 to 7,402 | 0.7756 |
| DSC vs. qISC | -6.995 | -17,95 to 3,955 | 0.2097 |
| ***IGF2R*** | | | |
| ISC vs. DSC | 3.318 | -8,297 to 14,93 | 0.5745 |
| ISC vs. qISC | -9.551 | -18,21 to -0,8938 | 0.0307 |
| DSC vs. qISC | -12.87 | -23,82 to -1,918 | 0.0214 |
| ***IL18R*** | | | |
| ISC vs. DSC | 1.16 | -10,45 to 12,77 | 0.8443 |
| ISC vs. qISC | 0.43 | -8,227 to 9,087 | 0.9222 |
| DSC vs. qISC | -0.73 | -11,68 to 10,22 | 0.8957 |
| ***IL6R*** | | | |
| ISC vs. DSC | 1.608 | -10,01 to 13,22 | 0.7855 |
| ISC vs. qISC | -10.6 | -19,26 to -1,945 | 0.0166 |
| DSC vs. qISC | -12.21 | -23,16 to -1,260 | 0.029 |
| ***LDLR*** | | | |
| ISC vs. DSC | 4.438 | -7,177 to 16,05 | 0.4527 |
| ISC vs. qISC | -8.031 | -16,69 to 0,6262 | 0.0689 |
| DSC vs. qISC | -12.47 | -23,42 to -1,518 | 0.0258 |
| ***NOTCH1*** | | | |
| ISC vs. DSC | 12.7 | 1,083 to 24,31 | 0.0322 |
| ISC vs. qISC | 1.039 | -7,618 to 9,696 | 0.8134 |
| DSC vs. qISC | -11.66 | -22,61 to -0,7079 | 0.037 |
| ***NOTCH2*** | | | |
| ISC vs. DSC | -5.23 | -16,84 to 6,385 | 0.3762 |
| ISC vs. qISC | -10.76 | -19,42 to -2,103 | 0.015 |
| DSC vs. qISC | -5.53 | -16,48 to 5,420 | 0.3211 |
| ***NOTCH3*** | | | |
| ISC vs. DSC | 0.94 | -10,67 to 12,55 | 0.8736 |
| ISC vs. qISC | 0.09667 | -8,560 to 8,754 | 0.9825 |
| DSC vs. qISC | -0.8433 | -11,79 to 10,11 | 0.8796 |
| ***NOTCH4*** | | | |
| ISC vs. DSC | 0.7 | -10,91 to 12,31 | 0.9057 |
| ISC vs. qISC | -2.312 | -10,97 to 6,345 | 0.5996 |
| DSC vs. qISC | -3.012 | -13,96 to 7,939 | 0.5887 |
| ***PTCH1*** | | | |
| ISC vs. DSC | 1.193 | -10,42 to 12,81 | 0.84 |
| ISC vs. qISC | -2.944 | -11,60 to 5,713 | 0.5038 |
| DSC vs. qISC | -4.137 | -15,09 to 6,814 | 0.4578 |
| ***PTGER1*** | | | |
| ISC vs. DSC | -0.1875 | -11,80 to 11,43 | 0.9747 |
| ISC vs. qISC | -0.8875 | -9,545 to 7,770 | 0.8402 |
| DSC vs. qISC | -0.7 | -11,65 to 10,25 | 0.9 |
| ***PTGER2*** | | | |
| ISC vs. DSC | 0.2675 | -11,35 to 11,88 | 0.9639 |
| ISC vs. qISC | -2.698 | -11,35 to 5,960 | 0.5402 |
| DSC vs. qISC | -2.965 | -13,92 to 7,985 | 0.5945 |
| ***PTGER3*** | | | |
| ISC vs. DSC | 0.005 | -11,61 to 11,62 | 0.9993 |
| ISC vs. qISC | -0.04167 | -8,699 to 8,615 | 0.9924 |
| DSC vs. qISC | -0.04667 | -11,00 to 10,90 | 0.9933 |
| ***PTGER4*** | | | |
| ISC vs. DSC | 6.92 | -4,695 to 18,53 | 0.2419 |
| ISC vs. qISC | -6.425 | -15,08 to 2,232 | 0.1452 |
| DSC vs. qISC | -13.35 | -24,30 to -2,395 | 0.0171 |
| ***SMO*** |  |  |  |
| ISC vs. DSC | 0.4225 | -11,19 to 12,04 | 0.943 |
| ISC vs. qISC | 0.4825 | -8,175 to 9,140 | 0.9127 |
| DSC vs. qISC | 0.06 | -10,89 to 11,01 | 0.9914 |
| ***TGFBR1*** | | | |
| ISC vs. DSC | 0.6475 | -10,97 to 12,26 | 0.9127 |
| ISC vs. qISC | -3.936 | -12,59 to 4,721 | 0.3717 |
| DSC vs. qISC | -4.583 | -15,53 to 6,367 | 0.4108 |
| ***TGFBR2*** | | | |
| ISC vs. DSC | 4 | -7,615 to 15,61 | 0.4985 |
| ISC vs. qISC | -2.042 | -10,70 to 6,615 | 0.6429 |
| DSC vs. qISC | -6.042 | -16,99 to 4,909 | 0.2785 |
| ***TNFRSF1A*** | | | |
| ISC vs. DSC | 0.0825 | -11,53 to 11,70 | 0.9889 |
| ISC vs. qISC | -17.68 | -26,33 to -9,019 | <0,0001 |
| DSC vs. qISC | -17.76 | -28,71 to -6,808 | 0.0016 |
| ***TNFRSF1B*** | | | |
| ISC vs. DSC | -0.2125 | -11,83 to 11,40 | 0.9713 |
| ISC vs. qISC | 0.01083 | -8,646 to 8,668 | 0.998 |
| DSC vs. qISC | 0.2233 | -10,73 to 11,17 | 0.968 |

ISC, intestinal stem cell; DSC, deep secretory cells, qISC, quiescent stem cell

**Supplementary Table 7.** Sequences of the primer pairs used for RT-qPCR (produced by Metabion international AG, Planegg, Germany).

| **Target genes human:** | **Forward primer (5‘-3‘)** | **Reverse primer (5‘-3‘)** |
| --- | --- | --- |
| *ACTB* | *ACATCCGCAAAGACCTGTACG* | *TTGCTGATCCACATCTGCTGG* |
| *ALCAM* | *CTTCTGCCTCTTGATCTCCG* | *AAATACTGGGGAGCCATCG* |
| *ALOX15* | *TGGATGTCAAGGCCAAC* | *CATGTCAGAGACCAGCCCAG* |
| *ALOX5* | *TGCCAACAAAACAGACCCCT* | *GAAGGTGGGTGATGGTCTGG* |
| *ALPI* | *CCCGCTTTAACCAGTGCAAC* | *GGAAACATGTACTTGCGGCC* |
| *ATOH1* | *CCAGCTGCGCAATGTTATCC* | *TGCTGTTTTCCTCCTGCACT* |
| *BIRC5* | *GGACCACCGCATCTCTACAT* | *GTCGTCATCTGGCTCCCA* |
| *BMI1* | *ATGTGTGTGCTTTGTGGAGG* | *TCCGATCCAATCTGTTCTGG* |
| *CASP1* | *CAAGACCTCTGACAGCACGT* | *GCATCTGCGCTCTACCATCT* |
| *CD36* | *AAAACGGCTGCAGGTCAAC* | *TCACCACACCAACACTGAGT* |
| *CD44* | *TGCCGCTTTGCAGGTGTATT* | *CCGATGCTCAGA GCTTTCTCC* |
| *COX1* | *AGCCCTTCAATGAGTACCGC* | *ACTCCGGAGAACAGATGGGA* |
| *COX2* | *ATGATTGCCCGACTCCCTTG* | *AACTGATGCGTGAAGTGCTG* |
| *DGAT1* | *GAGCTACCCGGACAATCTGA* | *GCGTGAGTAGTCCATGTCCT* |
| *DLL1* | *ACTGAGAAGCTAGAGGGCCA* | *AGTCCGCCTTCTTGTTGGT* |
| *EGF* | *ACTTGTTTCCTGTCCACGCA* | *TGGTTGTGGTCCTGAAGCTG* |
| *HES1* | *GAAGAAAGATAGCTCGCGGCA* | *GGAAAGCAAACTGGCCATCG* |
| *HMGCS1* | *GCACAGTACTCACCTCAGCA* | *CGAAGACATCTGGTGCCACA* |
| *HOPX* | *CTTCAACAAGGTCGACAAGC* | *GTGACGGATCTGCACTCTGA* |
| *INSR* | *CGCTCTGGTGTCACTTTCCT* | *ATGTCGTTTCTCTCCTGGCG* |
| *KLF4* | *CCATCTTTCTCCACGTTCG* | *ATCGGATAGGTGAAGCTGCA* |
| *LDHA* | *GCACCCAGTTTCCACCATGA* | *GCACTCTTCTTCAAACGGGC* |
| *LGR5* | *CACACACTG TCATTGCGAG* | *GCTTCTGTGGGTACGTGTCTT* |
| *LRP5* | *CTGCATGGACTGAGGAACG* | *CGGCTGTAGATGTCGATGC* |
| *LRP6* | *ATCTCGACAGTCAGGGTGGA* | *CGGGGCTCTGTTGTTCATC* |
| *LYZ* | *GCCTAGCAAACTGGATGTGT* | *ATGCCTTGTGGATCACGGAC* |
| *MLH1* | *CCTACTTCCAGCAACCCCAG* | *TGAGGATTCACACAGCCCAC* |
| *MSH2* | *AGTGCAGCCAGAGATCTTGG* | *GGCATCCTGGGCTTCAT* |
| *MSI1* | *AGCCAAAGGAGGTGATGTCG* | *GCAGTGAGAGGAATGGCTGT* |
| *MUC2* | *GACGGAGCTGAAGTTGGAAG* | *GGACACGGAGATGTTGGAGT* |
| *PMS2* | *ATCTCGCAACCCCAAACACA* | *GAGTCTGAGGTGCTATGAGCC* |
| *RARRES2* | *GTGCAAAGTCAGGCCCAATG* | *GCGAACTGTCCAGGGAAGTA* |
| *REG4* | *TCCTATTGCTGAGCTGCCTG* | *CGGCATCAGACCAGTTCCTC* |
| *SLC2A1* | *TGGCATCAACGCTGTCTTCT* | *CTAGCGCGATGGTCATGAGT* |
| *SLC2A3* | *GGCTGCTTTATGGGACTGTG* | *CAAAATGGAAGGGCTGCACT* |
| *SLC2A4* | *TAGGCTCCGAAGATGGGGAA* | *GGAAAAGATGGCCACGGAGA* |
| *SLC5A1* | *TGCTGGTGGGGTCTTTAATC* | *GGATGGACATCCCAAAGATG* |
| *SPDEF* | *GATTCACTACTGTGCCTCGAC* | *ATGTCTGGCTTCCGGATGAT* |
| *UBC* | *CCGGGATTTGGGTCGCAG* | *TCACGAAGATCTGCATTGTCAAG* |
| **Target genes mouse:** | **Forward primer (5‘-3‘)** | **Reverse primer (5‘-3‘)** |
| *Rn18S* | *GCAAATTACCCACTCCCGAC* | *CCGCTCCCAAGATCCAACTA* |
| *Alpi* | *GGACATCGCCACTCAACTCA* | *TGGTCAATACGACCCCCTTC* |
| *Alox12* | *GCCTGCTGA GAATCCCAA GT* | *GGTGAGGAA ATGGCAGAGCT* |
| *Alox15* | *ATGGTGCTGAAGCGGTCTAC* | *ATCCGCTTCAAACAGAGTGC* |
| *Alox5* | *GCGCAAATACTGGCTCCAT* | *TACGTCTGTGCTGCTTGAGG* |
| *Atoh1* | *GTGGGGTTGTAGTGGACGAG* | *GTTGCTCTCCGACATTGGG* |
| *Bmi1* | *GCTCCAATGAAGACCGAGGA* | *CAGCTCTCCAGCATTCGTCA* |
| *Casp1* | *CTTCAATCAGCTCCATCAGC* | *CACGGCATGCCTGAATAATG* |
| *Cd44* | *CGGCGCTAAAGATGCAAGAA* | *TTCTGCCCACACCTTCTCCT* |
| *Cox1* | *AGTCGAAGGAGTCTCTCGCT* | *CCAGGTCCAGATCTCAGGGA* |
| *Cox2* | *ATGACTGCCCAACTCCCATG* | *ACTGATGGGTGAAGTGCTGG* |
| *Daglβ* | *GGTGGCCGCTCTACATCTAC* | *GGGCAGCTCATACACCTTGT* |
| *Dgat1* | *TCTCTTAAAGCTGGCGGTCC* | *CCACTTGTGCACGGGGATA* |
| *Hes1* | *CAAACCAAAGACGGCCTCT* | *GTCACCTCGTTCATGCACTC* |
| *Hopx* | *TCAACAAGGTCAACAAGCACC* | *CCGTAACAGATCTGCATTCCG* |
| *Insr* | *CAGCAACTTGATGTGCACCC* | *CATAGGAGCGGCGGATCTTT* |
| *Klf4* | *GGGAAGGGAGAAGACACTGC* | *GGTGGGTTAGCGAGTTGGA* |
| *Ldha* | *TTGCCCTTGTTGACGTCATG* | *GCCACTGATTTTCCAAGCCA* |
| *Lgr5* | *CGGCAACAGTGTGGACGACCT* | *GCGAGCACTGCACCGAGTGA* |
| *Lpl* | *TTCAGAGTTTGACCGCCTTC* | *AAGGTCTTGCTGCTGTGGTT* |
| *Lpo* | *CCCCTGCTCCAACAATGACT* | *GGGAACATGATGGGGAAGCA* |
| *Lyz* | *GCCAAGGTCTACAATCGTTGTGAGTTG* | *CAGTCAGCCAGCTTGACACCACG* |
| *Mki67* | *CCTGCCCGACCCTACAAAAT* | *TTGCTCACACTCGATGCAGT* |
| *Mlh1* | *TTCACCCAGACCTTGCTTCC* | *AGCTGTCATTTCCTTCCCGG* |
| *Msh2* | *ATGGGGAAACTGAGGCAGG* | *TTGAGGGCTCTAACTGCTGC* |
| *Msi1* | *GAGCAGTTCGGAAAGGTGGA* | *ACATCACCTCCTTTGGCTGG* |
| *Pdh* | *GCTGCTGAAGAGGCGTTTT* | *TCAGAGATGGGCTTTGCTG* |
| *Pkm* | *TGAAGAAGGGAGCCACTCTG* | *CCAACATCCATGGCCAAG* |
| *Pms2* | *TGTGTTTGGCCAGAAGCAGT* | *AGTGGCTGCTGGTTGACATT* |
| *Reg4* | *CCCGAAGTCCTGAGCGATAT* | *CCACACAGGCAGGTTTCTCT* |
| *Rarres2* | *GCCTTCCAAGAGATCGGTGT* | *ATTTCCGCCTTCTCCCGTTT* |
| *Rn18S* | *GCAAATTACCCACTCCCGAC* | *CCGCTCCCAAGATCCAACTA* |
| *Spdef* | *GGAGAAGGCAGCATCAGGA* | *CCAGGGTCTGCTGTGATGT* |
| *Tert* | *ACTCCGTTGTCATCGAGCAG* | *GTAAAAGCAACCCATCCCGC* |

**Supplementary Table 8.** Applied primary and secondary antibodies.

| **Primary antibodies** | **Species** | **Company** | **Working concentration** |
| --- | --- | --- | --- |
| Anti-α-TUBULIN (#2125) | rabbit | Cell Signaling Technology, Danvers, Massachusetts, USA | 1:1000 (WB)^a^ |
| Anti-PROCASPASE-1 +p10 +p12 (#ab179515) | rabbit | Abcam, Cambridge, United Kingdom | 1:1000 (WB)^a^ |
| Anti-KI67 (D3B5) (#12202) | rabbit | Cell Signaling Technology, Danvers, Massachusetts, USA | 1:400 (IHC)^a^ |
| Anti-MSH2 (#2017) | rabbit | Cell Signaling Technology, Danvers, Massachusetts, USA | 1 µg/ml (WB) |
| Anti-PHOSPHO-AKT (Ser473) (D9E) XP (#4025) | rabbit | Cell Signaling Technology, Danvers, Massachusetts, USA | 1:1000 (WB)^a^ |
| Anti-AKT (#9272) | rabbit | Cell Signaling Technology, Danvers, Massachusetts, USA | 1:1000 (WB)^a^ |
| Anti-p44/42 MAPK (Erk1/2) (137F5) (#4695) | rabbit | Cell Signaling Technology, Danvers, Massachusetts, USA | 1:1000 (WB)^a^ |
| Anti-PHOSPHO-p44/42 MAPK (Erk1/2) (Thr202/Tyr204) (D13.14.4E) XP (#4370) | rabbit | Cell Signaling Technology, Danvers, Massachusetts, USA | 1:1000 (WB)^a^ |
| Anti-AMPKα (#2532) | rabbit | Cell Signaling Technology, Danvers, Massachusetts, USA | 1:1000 (WB)^a^ |
| Anti-PHOSPHO-AMPKα (Thr172) (40H9) (#2535) | rabbit | Cell Signaling Technology, Danvers, Massachusetts, USA | 1:1000 (WB)^a^ |
| Anti-MUC2 (#GTX63560) | rabbit | GeneTex, California, USA | 1:1000 (WB)^a^ |
| Anti-INSR ß (E9L5V) (#23413) | rabbit | Cell Signaling Technology, Danvers, Massachusetts, USA | 1:1000 (WB)^a^ |
| Anti-15-LO (H-235) (#sc-32940) | rabbit | Santa Cruz Biotechnology, Inc., Dallas, Texas, USA | 2 µg/ml (WB) |
| **Secondary antibodies/**  **labelled polymers** |  | **Company** | **Working concentration** |
| Anti-rabbit IgG HRP |  | Cell Signaling Technology, Danvers, Massachusetts, USA | 1:4000 (WB)^a^ |
| EnVision anti-rabbit HRP system |  | Agilent, Santa Clara, California, USA | n.a. |
| EnVision anti-mouse HRP system |  | Agilent, Santa Clara, California, USA | n.a. |

**^a^** If stock concentration was not provided by the manufacturer, dilution from stock solution was listed.
n.a., not applicable/not available
